# Inside the battery box: a cross-technology assessment of PFAS uses, functions, and substitution potential

**DOI:** 10.1039/d6em00349d

**Published:** 2026-11-12

**Authors:** Amanda Rensmo, Anna J. Miller, Juliane Glüge, Martin Scheringer, Zhanyun Wang, Rainer Lohmann, Carla A. Ng, Xenia Trier, Ian T. Cousins

**Affiliations:** a Department of Environmental Science, Stockholm University SE-10691 Stockholm Sweden amanda.rensmo@aces.su.se ian.cousins@aces.su.se; b Institute for Biogeochemistry and Pollutant Dynamics, ETH Zurich 8092 Zurich Switzerland; c Department of Green Technology, Danish Institute for Advanced Study (DIAS), University of Southern Denmark 5230 Odense Denmark; d Empa – Swiss Federal Laboratories for Materials Science and Technology, Technology and Society Laboratory 9014 St. Gallen Switzerland; e NCCR Catalysis, ETH Zürich 8093 Zürich Switzerland; f Graduate School of Oceanography, University of Rhode Island Narragansett RI 02882 USA; g Department of Civil & Environmental Engineering and Environmental and Occupational Health, University of Pittsburgh Pittsburgh 15261 Pennsylvania USA; h Department of Plant and Environmental Sciences, Section for Environmental Chemistry and Physics, University of Copenhagen Thorvaldsensvej 40 DK-1871 Frederiksberg C Denmark

## Abstract

Understanding where and why per- and polyfluoroalkyl substances (PFASs) are used in batteries is essential for evaluating their substitution potential, yet detailed information across battery technologies is scarce. We analyzed more than 5600 submissions to the Annex XV public consultation on the proposed universal PFAS restriction in the European Union and extracted information reporting use, associated substance(s), and stated function(s) of PFASs in batteries. 341 records were compiled into a database using our structured Battery Use & Function Framework that incorporates the Functional Substitution Framework. Among these records, 78 contained sufficient information to qualify as a potential PFAS use and were organized into 36 potential PFAS uses, accounting for overlaps. Each potential PFAS use was evaluated using supporting evidence from previous use-mapping studies, input from battery stakeholders, teardown-analysis studies, and other targeted academic literature. Across all lines of evidence, two PFAS uses in batteries were contradicted, indicating already large-scale availability of PFAS-free alternatives, while 16 PFAS uses were confirmed, primarily in cathode components across several battery technologies and chemistries. PFAS-free alternatives were identified for 13 PFAS uses across battery applications, with an additional five uses showing PFAS-free options for certain applications within the broader use category. The findings indicate that few technical barriers remain for substituting PFASs in most battery components. Our mapping provides a detailed foundation for future alternatives assessments, supports essential-use evaluations, and highlights opportunities and challenges relevant to monitoring and recycling.

## Abstract 2

Reports on PFAS uses in batteries were collected and weighed against supporting evidence. Feasible alternatives were assessed using market deployment as a proxy for technical viability, illustrating a new cross-sector assessment framework.

## Graphical abstract

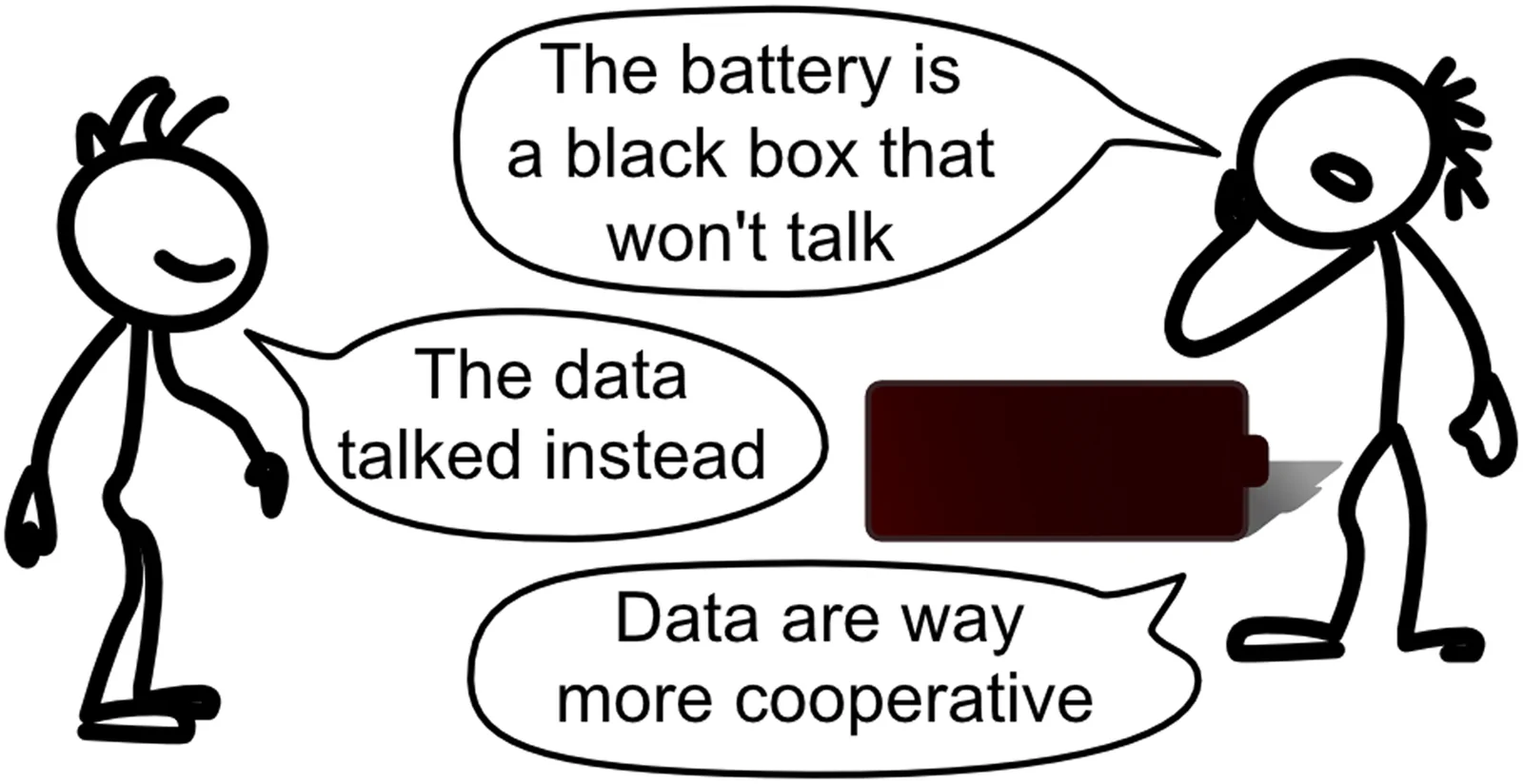

**Environmental significance**

The ongoing green energy transition involves a shift from fossil fuels to renewables and has resulted in a large expansion in the battery market. However, environmental impacts extend beyond mitigating the greenhouse effect. For example, the planetary boundary for novel entities (including substances such as PFASs) is being transgressed at increasing risk. This work lists PFAS uses in batteries to a larger extent than previously documented, using a weight-of-evidence approach to assess the likelihood of PFAS use in commercial batteries. In addition, potential alternatives are identified based on available data. Publicly confirmed information on PFAS uses, substances and functions are valuable across many contexts; these findings offer insights for alternatives assessments and mitigation of PFAS emissions from the battery life cycle.

## 1. Introduction

Per- and polyfluoroalkyl substances (PFASs), which with only a few exceptions are substances containing a –CF_2_– and/or –CF_3_ moiety,^1^ are currently proposed for restriction under the European Union's (EU's) chemicals legislation Registration, Evaluation, Authorisation and Restriction of Chemicals (REACH). The aim of this regulatory action is to address the risks these highly persistent chemicals pose to the environment and human health. In 2023, the universal PFAS restriction proposal (Annex XV report) was submitted to the European Chemicals Agency (ECHA), and in 2025, it was updated as the so-called Background Document to include information from the Annex XV public consultation. Under the proposal, all PFAS uses, except those explicitly exempted or regulated elsewhere would be regulated. Specifically, the manufacture, placing on the market and use of the substances on their own and the placing on the market of mixtures and articles would be regulated.^2,3^ The proposal was assessed by the expert committees of ECHA (Risk Assessment Committee (RAC) and Socioeconomic Analysis Committee (SEAC)). In March 2026, RAC published their final opinion on volumes, emissions and risk of alternatives for the listed PFAS uses. In parallel, SEAC published a draft opinion on the availability of alternatives, both technical and economic, among other things, which is to be finalized before the end of 2026.^4,5^

In general, owing to the high chemical strength of fully fluorinated carbon chains and versatile functional groups, PFASs have been used in many applications and sectors.^6^ Prior work has catalogued PFAS uses and/or PFAS-free alternatives. For example, Glüge *et al.* (2020) provided a first overview with more than 200 use categories and sub-categories.^6^ Furthermore, Figuière *et al.* (2025) identified 530 PFAS-free alternatives to a total of 325 PFAS uses.^7^ However, many of these previous use-mapping studies lack detailed information about the functions that PFASs provide in many uses, particularly in complex articles. In this work, we map both PFAS uses and their functionalities in greater detail for the case of batteries.

A battery converts chemical energy into electrical energy. A simple battery cell is made up of three main components: two electrodes (anode and cathode) and an electrolyte. The battery performance is highly dependent on the choice of electroactive material in the cathode (also referred to as the battery chemistry) as the potential between the electrodes drives the spontaneous transfer of electrons. Electrical energy is obtained when electrons flow from the anode to the cathode through the external circuit, while charge balance is maintained by ion transport through the electrolyte (Fig. 1a). For rechargeable batteries, the flow of electrons and ions can be reversed, converting electrical energy into chemical energy stored for future discharge. A rechargeable battery is also referred to as a secondary battery, while a non-rechargeable battery is referred to as a primary battery.^8,9^

**Fig. 1:**
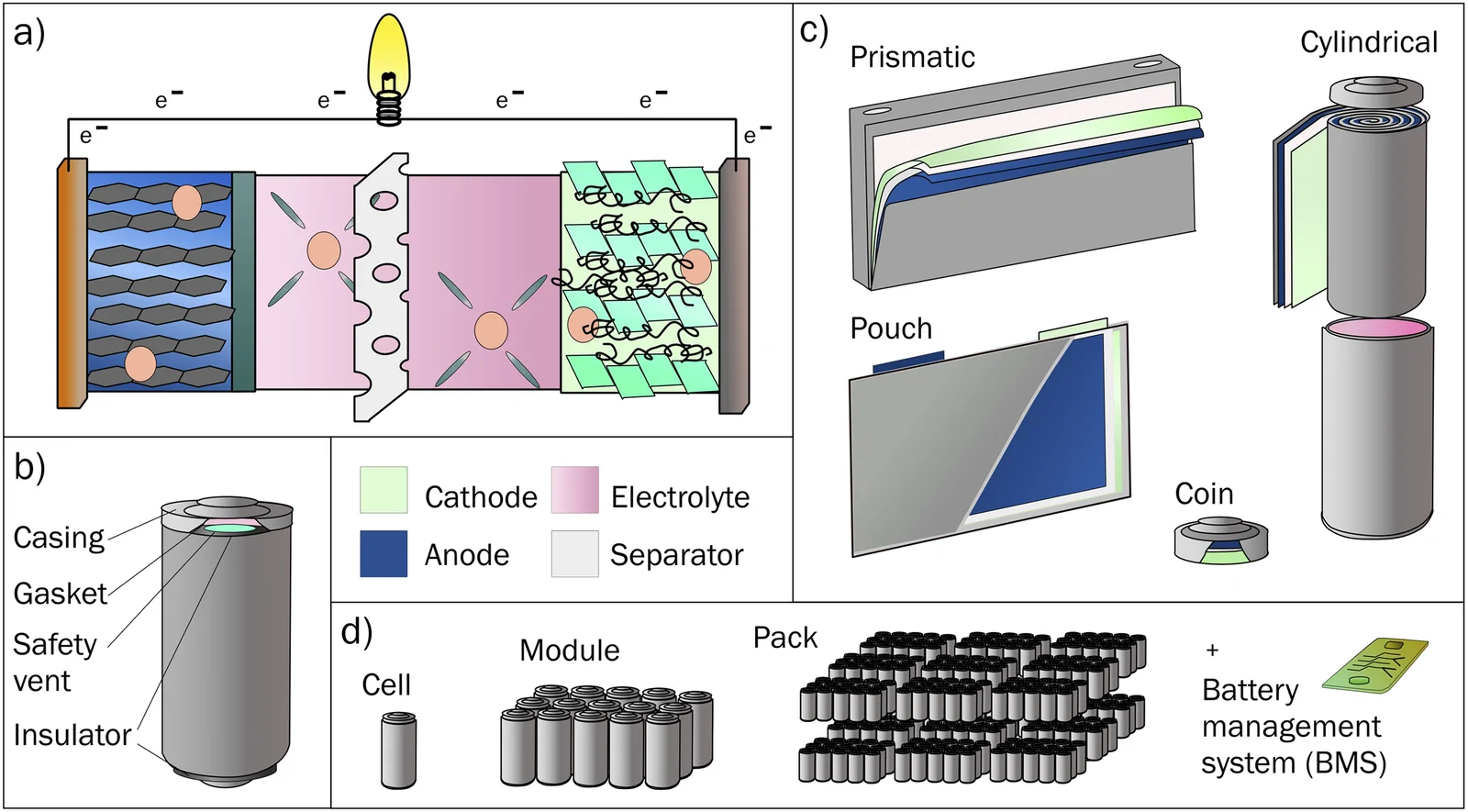
(a) Schematic of a (Li-ion) battery including a cathode, anode with a solid electrolyte interface, liquid electrolyte with coordinated Li-ions, porous separator and electric circuit connected to the current collectors; (b) schematic of components of the outer shell of a battery; (c) illustration of common battery configurations, where the electrolyte is only distinguishable in the cylindrical configuration; and (d) units of a battery, extending a cell into a module, pack, and battery management system (BMS, where the electronic system is illustrated with a printed circuit board).

Apart from the electrodes and the electrolyte, additional components are required for a functioning battery. A separator is often placed in the electrolyte between the electrodes, preventing short-circuit. Additionally, current collectors are attached to the anode and cathode to facilitate electron transport to the external circuit (Fig. 1a). Lastly, auxiliary components such as casing and gaskets ensure containment and mechanical stability, supporting safe and reliable handling (Fig. 1b). The cell format can vary, where four configurations are most common: cylindrical, prismatic, coin, and pouch (Fig. 1c). Commercial systems may include several individual cells connected in series and/or parallel to form a battery module, which in turn can be assembled into a pack and controlled by a battery management system (BMS) (Fig. 1d).^8,9^

The overall battery performance is linked to a wide range of technical characteristics, including capacity and voltage, energy density, power density, lifetime, cycle life, self-discharge rate, operational temperature, and safety, among others.^8,9^ Battery manufacturers tailor their product according to the market needs, *i.e.*, downstream users' preferences. Examples include large-scale storage (prioritizing cost over energy density), electric vehicles (EVs, where energy density and charging rate are both important), medical implants (requiring long lifetime) and miscellaneous consumer products (varying depending on the specific application but often requiring low cost).^8^ The requirements for these applications dictate which battery technology is (most) suitable. For example, the 9 V batteries used in household smoke detectors are typically primary alkaline batteries, valued for their low cost, while applications requiring high energy density such as EVs often use Li-ion batteries.^9^

Even for a specific battery technology, some battery chemistries are preferred over others in specific applications. As battery technologies change rapidly over time, preferences may change. For example, the lithium nickel manganese cobalt oxide (LiNi_1−*x*−*y*_Mn_*x*_Co_*y*_O_2_, NMC) battery was said to be the most suitable battery chemistry in EV for a long time; however, today many manufacturers adopt lithium iron phosphate (LiFePO_4_, LFP) chemistry because of its lower cost, among other aspects.^10^

An initial survey of PFASs in batteries was conducted by Rensmo *et al.* (2023), the study only identified fluorinated substances in Li-ion batteries as described in the academic literature and did not systematically map PFAS uses across all battery technologies.^11^ Savvidou *et al.* (2024) followed with an investigation of PFAS-free alternatives in Li-ion batteries, but that provided only an initial, high-level assessment.^12^ Here, we extended the knowledge base by mapping PFAS uses and associated PFAS-free alternatives across a broader range of battery technologies, chemistries, and components.

First, we compiled relevant information from the more than 5600 comments to the Annex XV public consultation to the universal PFAS restriction proposal to create a database of reported PFASs in batteries, as described, and mapped the stakeholder information according to a Battery Use & Function Framework. We developed the framework linking use information to substance *via* the function. Uses were categorized into three levels (application, technology, and component), in which the technology level was divided in sub-levels (type, chemistry, and unit). Second, potential PFAS uses were qualified and evaluated using supporting evidence. Within all supporting evidence, we focused on information regarding use, substance, and function, particularly uses with high market volumes (*i.e.*, not laboratory scale).

To enable identification of potential alternatives, information on the function of PFASs in each use in the Battery Use & Function Framework was categorized according to the Functional Substitution Framework.^13^ Previous searches for alternatives often investigated drop-in replacements that have the same or similar chemical function, which can lead to so-called “regrettable substitution” in which the replacement substance exhibits intrinsic properties (*e.g.*, persistence, physical–chemical behaviors or hazards) similar to those of the predecessors.^14^ Thus, Tickner *et al.* (2015) defined three levels of functional substitution: technical (equivalent to chemical), end-use, and service functions.^13^ Substitutions based on the end-use or service function provide a broader perspective, enabling a wider range of alternative solutions that may reduce the likelihood of regrettable substitution. Detailed use-level information is also crucial for evaluation under the essential-use concept.^15,16^ As a result, we defined a potential alternative as a PFAS-free substance, material, design, process and technology on the market.

Finally, we incorporated all lines of evidence to determine the likelihood of PFAS uses and associated PFAS-free alternatives in batteries on the market. As a result we extended single-company perspectives to the broader sectoral context. A complete alternatives assessment is considered beyond the scope of this work. Rather, we aim to provide information on PFAS uses and functions with a sufficient level of detail to support future, more comprehensive alternatives assessments, and by extension, for evaluating PFAS uses in batteries under the essential-use concept.

## 2. Methods

The study consisted of two parts (Fig. 2). Data consisted of Annex XV consultation comments and supporting evidence, the latter collected through exchanges with identified battery stakeholders as well as from peer-reviewed articles, reports, and other types of studies. As such, supporting evidence was gathered from industry including trade organizations, regulatory agencies, non-governmental organizations, and academia. Industry was represented across the battery value chain, including material producers, battery component manufacturers, battery manufacturers, and downstream users. The SI contains an in-depth description of the method, see SI1, Section 2.1. through 2.2. and SI2, Section 2.1. through 2.4. The results are presented in SI1, Section 3.1. through 3.5., SI2, Section 3.1. through 3.3., and SI3. A thorough analysis of the results, providing the basis for our conclusions, is provided in SI2, Section 4.

**Fig. 2:**
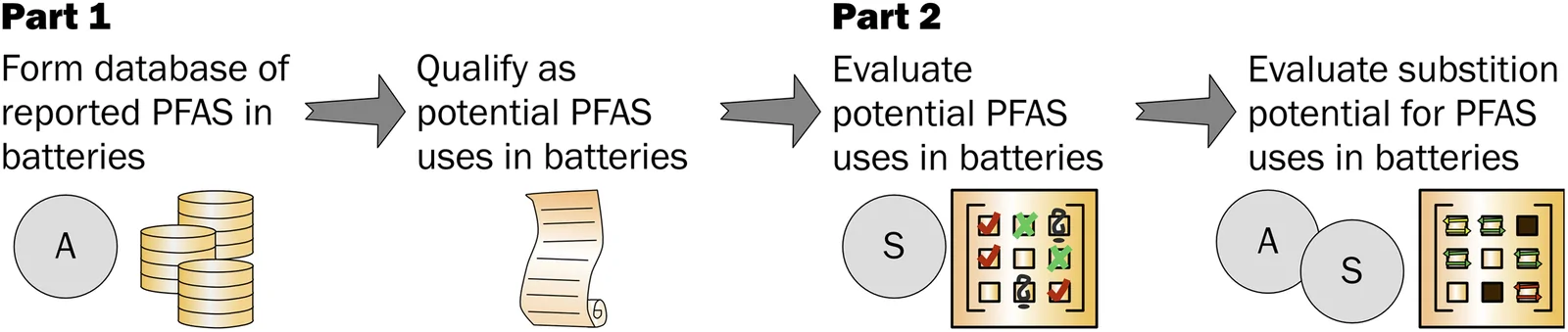
Methodology of study, divided into two parts. The first part applies the Battery Use & Function Framework and assesses data availability using a decision-making model. The second part adopts a weight-of-evidence approach to determine the likelihood of PFAS use in batteries and their substitution potential. The study includes two main types of data: A – Annex XV consultation comments and S – supporting evidence.

### 2.1. Qualifying a potential PFAS use in batteries

#### 2.1.1. Collection of information

In 2023, ECHA conducted a six-month public consultation (the Annex XV public consultation) on the universal PFAS restriction proposal. Stakeholders were asked to provide information according to ten questions on topics including missing uses, (non)availability of alternatives, economic and technical feasibility of alternatives, information on emissions, analytical methods, and impacts on the recycling industry. All non-confidential submissions (here referred to as Annex XV consultation comments) were then published by ECHA, which included the general comments submitted according to the survey outline (here referred to as survey responses) as well as attachments.^17^

We searched both survey responses and attachments using the search string “batter*” (to include both ‘battery’ and ‘batteries’), which resulted in the identification of 341 survey comments and 1140 attachments (including duplicates). An adjusted search string was formulated for the attachments only as “batter* AND function,” which resulted in the identification of 401 attachments (including duplicates). The addition of the term “function” was justified by the large number of identified records for “batter*” alone among the attachments and by the study's aim of identifying the specific functions of PFASs in batteries. Redox flow batteries, electric capacitors and similar were considered outside the scope of this study as their energy storage mechanisms are different from those of traditional batteries. The identified records were thereafter screened according to a set of eligibility criteria, *e.g.*, they were excluded if they only referred to proposed derogations or grouped batteries together with other uses in the energy sector without additional details (SI1, 2.1.1.). A total of 75 survey comments and 67 attachments from a total of 131 stakeholders were included in the database.

#### 2.1.2. Development of the Battery Use & Function Framework

As described in the Introduction, we developed a Battery Use & Function Framework to link use information to substance(s) and the function(s) provided. The framework defines three Battery Use levels, with three additional sub-levels nested within Battery Use level 2, as outlined in Table 1 (SI1, 2.1.2.).

**Table 1:** Battery Use levels as defined in this work

Battery Use level		Description
1	Component	Refers to the simple article in the battery. Examples include the cathode, anode, separator, electrolyte, gasket, and lubricant, among others
2	Battery technology	Describes a set of dimensions of a battery, including the electrochemical system; examples include the Li-ion battery, Na-ion battery, solid-state battery, alkaline battery, *etc.*
2.1	Battery type	Denotes rechargeability of battery technology; only two available options including primary or secondary
2.2	Battery chemistry or composition of relevant component	Denotes the cathode composition or information about the anode and/or electrolyte if relevant. Examples include LFP or NMC (battery chemistry), hard carbon or graphite (anode), and liquid or ceramic (electrolyte)
2.3	Battery unit	Denotes the unit in a battery; only four available options including the cell, module, pack, and battery management system (BMS)
3	Application	Describes the area of use of the specific battery technology; options not constrained

In addition to our Battery Use levels, the function (of the identified substance in the specific use) is also listed for each entry into the database. Here, the Functional Substitution Framework, as developed by Tickner *et al.* (2015) was applied to provide three levels of functions, namely technical, end-use, and service function.^13^ In our work, the Functional Substitution Framework is not only a categorization tool but also a diagnostic lens: it allows us to distinguish between uses where PFASs provide a unique technical function and uses where PFASs are simply one way of achieving an end-use or service function that can also be delivered by non-PFAS materials.

The Functional Substitution Framework was adapted to recognize the complexity of batteries (Table 2). The technical function is linked directly to the Organisation for Economic Co-operation and Development's (OECD) 124 functional use categories, which are relevant for simple articles (in our case, the battery components).^18^ However, we note that the two other function levels (end-use and service function) in the Functional Substitution Framework are not directly transferable to the case of batteries. Thus, we consider that the end-use function reflects that the component is part of a complex article (the battery technology) and considers functions in a broader sense than listed by the OECD. For example, for the technical function “binder” of a substance in the cathode, the end-use function could be “improving conductivity” as this binder would allow the electroactive material to stick to the current collector. Third, since all batteries provide the service function “storage of energy,” this would not bring additional information to our work. Instead, we recognized that energy storage performance requirements could vary amongst different applications. Therefore, we decided to not only list “energy storage” as the service function but extend the description including available information related to *e.g.*, extended lifecycle, higher safety, and enhanced charging performance, such as higher charge rates or greater charge acceptance. Lastly, stakeholders may have provided information about technical requirements, which describe a performance level that must be fulfilled for a certain application. Thus, this level, going beyond the Functional Substitution Framework, describes a parameter that can be quantified, providing insights into the acceptable level of performance. Together, the Battery Use levels and the adapted Functional Substitution Framework levels make up the Battery Use & Function Framework.

**Table 2:** Adaptation of the Functional Substitution Framework in the Battery Use & Function Framework

Function level	Addresses the following criteria or question
Substance	Substance described by a stakeholder, as a group or individual substance, including polymers. Examples include “PFAS”, “fluoropolymers”, TFSI, and R1234yf, among others
Properties	What are the properties inherent to the substance on its own? Examples include chemical resistance, durability, and mechanical stability, among others
Technical function	What chemical function is provided to the component by the substance? Defined according to the OECD functional use categories denoting “the specific function that a chemical provides when used in the formulation of a product or article, or when used within an industrial process”.^18^ Examples include a binder, sealant (barrier), flame retardant, and adhesion/cohesion promoter, among others
End-use function	What does the substance in the component bring as a function to the component? Examples could include limiting risk of short circuit (separator), improving oxidation resistance (electrolyte), correcting for overpressure (membrane), and improving conductivity (electrolyte), among others
Service function	With the given substance(s), what does the component do for the overall performance of the battery? Examples could include longer life, energy efficiency, safety, and reliable operation, among others
Technical requirement	What technical requirements (quantitative performance criteria or given condition) are set for the given component, battery technology, and/or application to fulfill an acceptable level of performance? Examples could include “withstand a high oxidation atmosphere (4.2 to 4.5 V Li/Li^+^)”, “lifetime of 20 years”, pass “flame-retardant test *e.g.*, UL94 V0”, and [possess] “chemical stability to thionyl chloride”, among others

#### 2.1.3. Recording information in the database

The eligible reports were read in their entirety, and all relevant information was included in the database, identifying, screening, and including Annex XV consultation comments, according to an adapted version of the PRISMA 2020 flowchart^45^ (SI1, 2.1.3.). Eligible information was included in our database (SI2, 3.1.).

The database, exemplified in Table 3, consists of entries following the outlines in Table 1 (uses) and Table 2 (functions). In addition, the database includes information on substances and Annex XV stakeholder identities (in our work defined as reference ID; SI2, 2.1.).

**Table 3:** Examples of using the Battery Use & Function Framework to record reports of PFASs in batteries. Data shown here do not correspond directly to records in our database. Asterisk (“*”) means that this information was inferred from other entries within the same record (*e.g.*, a cathode is part of a cell) and semi-colon (“;”) denotes inseparable information into one entry (*e.g.*, a source noting that “PVDF and PTFE are used as cathode binders in LCO and NMC batteries”, as depicted in record 3)

	Battery Use level 1: battery component	Battery Use level 2: battery technology	Battery Use level 2.1.: battery type	Battery Use level 2.2.: battery chemistry	Battery Use level 2.3.: battery unit	Battery Use level 3: application	Substance	Property	Technical function	End-use function	Service function	Technical requirement	Reference
Record 1	No information	Li-ion battery	Secondary*	No information	Cell*	Cathode	PVDF	No information	Binder	No information	No information	No information	Ref. 1 and Ref. 2
Record 2	Electric vehicle	Battery	No information	No information	Cell*	Cathode	PVDF	No information	Binder	No information	No information	No information	Ref. 3
Record 3	No information	Li-ion battery	Secondary	LCO; NMC	Cell	Cathode	PVDF; PTFE	Chemical stability; thermal stability; electrochemical stability; dispersive capability	Binder; adhesion/cohesion promoter	Mechanical integrity of the electrode	Performance (long cycle life)	Potential > 3 V	Ref. 4
Record 4	No information	Lithium battery (CR, cylindrical)	Primary	No information	Cell*	Electrolyte	LiTFS	Solvent affinity; heat resistance; electrochemical stability	Conductive agent	Low heat generation	Long term reliability	No information	Ref. 4
Record 5	Power tools	Li-ion battery	Secondary	LCO	Cell	Cathode	PVDF	No information	No information	No information	No information	No information	Ref. 5

Each row in the database is denoted as a record of PFAS use in batteries. That is, even if several records note the use of PFASs as a binder in the cathode, they could differ in the technology, such as “Li-ion battery” compared to the unspecified “Battery,” and these would therefore be considered as separate records (see Table 3, records 1 and 2). Only when all entries for a record were identical did we treat information from multiple submissions as a single entry (see Table 3, record 1). This approach acknowledges that some Annex XV consultation comments were duplicates and therefore we limit the potential for skewed results.

Entries were filled in *verbatim* from stakeholders (including potential errors and spelling mistakes) with a few exceptions. First, some information was altered to fit the nomenclature as provided in Table 1. Conformity was obtained by using one naming convention for each battery technology. For example, Annex XV consultation comments could state “LIB”, “Li-ion battery”, and “lithium-ion battery”, which were all translated to “Li-ion battery” battery. However, “Lithium battery” was not translated to a “Li-ion battery” battery, as this could refer to many battery technologies, of both secondary and primary type. Second, sometimes information for a use level was not explicitly given but could be inferred from other entries within the same record (*e.g.*, a Li-ion battery is always secondary, or a cathode is always part of a cell). In cases noting “LFP battery” or other chemistries relevant to Li-ion batteries, this was also translated to “Li-ion battery” battery for Battery Use level 2. Third, information provided by the stakeholders related to the technical function was converted to the set categories of the OECD functional use categories. Finally, if no information was available for a specific level for each record, this was noted with “no information.”

#### 2.1.4. Qualifying records as a potential PFAS use

Nearly all of the consultation comments lacked a description of their scope, methodology, and analysis, as well as raw data for conclusions concerning the PFAS use and the (non-)availability of alternatives. Therefore, we consider each Annex XV consultation comment with equal reliability, as we do not have any additional information on how to weight each.

After categorizing and adding all information in the database, the availability of information was assessed for each record with a decision-making model (Fig. 3) and if the record had sufficient information on uses, we qualified the record as a “potential PFAS use.”

**Fig. 3:**
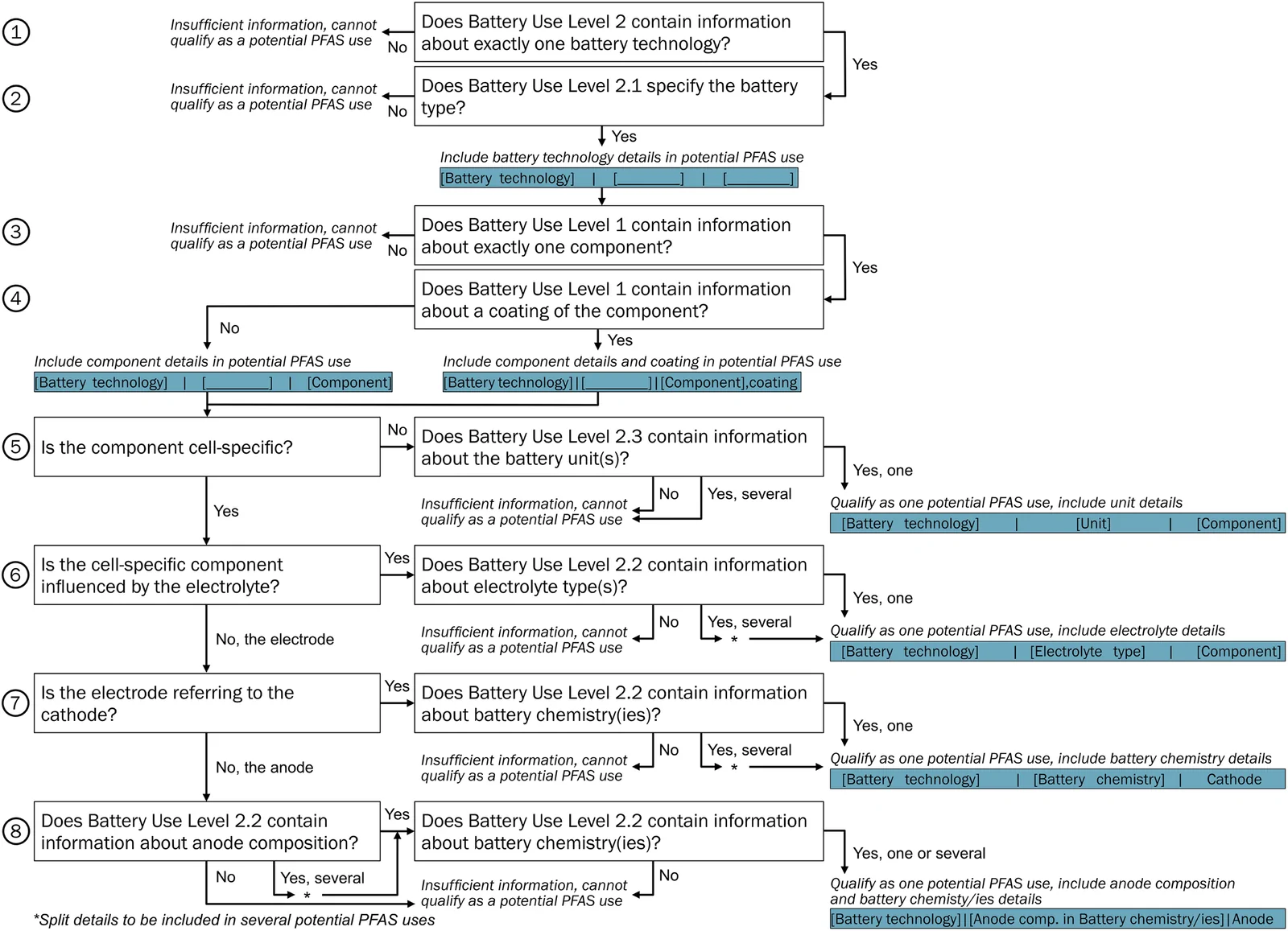
Decision-making model used to assess whether sufficient information is available to qualify a record as a “potential PFAS use”, incorporating information from Battery Use levels 1 and 2 (including sub-levels). Light blue boxes indicate inclusion of information into each Battery Use level. Examples include: Li-ion/Cell/Seal gasket (19, routed through step 5); Li-ion/Liquid electrolyte/Separator, coating (17) and Na-ion/Liquid electrolyte/Electrolyte (25, both routed through step 6); Li-ion/LFP chemistry/Cathode (6) and Li-ion/Metal oxide chemistries/Cathode, coating (11, both routed through step 7); and Na-ion/Hard carbon in PBA chemistry/Anode (24) and Li-ion/Graphite in LFP, LMO, NMC, and NCA chemistries/Anode (12, both routed through step 8).

The decision-making model for evaluating whether each record contains sufficient information is structured in several steps. It starts with three questions, steps 1 through 3, focusing on assessing whether entries in Battery Use levels 1 and 2 included sufficient and consistent data, accounting for stakeholders often listing multiple battery technologies or components (as exemplified in Table 3, record 3). The fourth question, step 4, clarifies whether the relevant part of the component is a coating or the main component.

After listing the battery technology and component, we provide several steps to assess the information on the relevant sub-use level, which differs depending on the component. Thus, step 5 recognizes that a battery is more than just the cell, including the module, pack, or battery management system. We further acknowledge that even within a single battery technology, there is considerable variation. To manage this complexity without demanding excessive detail, the scheme adds three additional steps incorporating Battery Use level 2.2. First, in step 6, the decision-making model distinguishes between components affected by the electrolyte (such as the separator or the electrolyte) and those related to the electrodes (the cathode and the anode). Since a battery technology is defined by its chemistry, we consider it necessary for a potential PFAS use describing the electrode to include information on the battery chemistry, as outlined in step 7. Lastly, step 8 addresses that the anode additionally requires information on the anode composition. Overall, this decision-making model helps bridge the information gap between battery technology and battery component, ensuring that the data collected are consistent with what is needed for valid analysis.

If several records resulted in an identical potential PFAS use, they were consolidated into one. Our approach therefore reduces the risk of overestimating the number of PFAS uses in batteries. For example, of the records listed in Table 3, only record 3 and record 5 contain sufficient information for listing as potential PFAS uses (*i.e.*, Li-ion/LCO chemistry/Cathode and Li-ion/NMC chemistry/Cathode). In steps 6 through 8, we allow details for Battery Use level 2.2. (battery chemistry, anode or electrolyte composition) to be assigned to multiple uses because the remaining characteristics of these details are generally very similar. This approach ensures that we avoid excluding an excessive number of records. However, in Step 5, details for Battery Use level 2.3. (battery unit) cannot be split across multiple potential PFAS uses because the characteristics of the different units vary significantly.

### 2.2. Integrating supporting evidence

To assess whether the records of PFAS uses collected from the Annex XV consultation comments are commercially relevant and needed (rather than only claimed as potentially useful), we sought out supporting evidence. Here, we also recognized that the information contained in the Annex XV consultation comments represents a snapshot in time and more recent developments since 2023 are not reflected in the comments assembled during the consultation.

The supporting evidence was collected from five source types, described below in Sections 2.2.1.–2.2.5. below. First, we surveyed previous use-mapping studies. Data were also gathered through engagement with industry experts. In addition, use in articles on the market was surveyed through teardown-analysis studies. Three cases were selected for further data collection from the academic literature. In parallel, we also noted information on alternatives presented in both the Annex XV consultation comments and the supporting evidence. As a result, the supporting evidence considered in our assessment primarily reflected PFASs in articles on the market rather than PFASs used in laboratory-scale research. Although quantitative data on volumes could not be identified, we consider the presence of PFASs in marketed batteries to be implicitly confirmed, for example through teardown-analysis studies of batteries in common electric vehicles and through the overall assessments provided by individual stakeholders.

#### 2.2.1. Previous use-mapping studies

Previous use-mapping studies were identified *via* expert knowledge and included peer-reviewed literature, regulatory reports, and studies by NGOs (SI1, 2.2.1.). References in the identified literature were also included to further expand the search, for example, Kissa (2001) and Ameduri (2018) were included as sources in Glüge *et al.* (2020).^6,19,20^ In our work, we consider the Annex XV report and Background Document as previous use-mapping studies.^2,3^ A total of 20 (not counting newer editions) previous use-mapping studies were included.^2,3,6,7,11,12,19–32^ All reports were surveyed for information related to use information of PFASs in batteries (SI1, 3.1.).

#### 2.2.2. Input from battery stakeholders

In addition, we initiated direct contact with battery stakeholders, including trade organizations or companies that are involved in the battery supply chain such as raw materials providers, battery manufacturers and downstream users. A list of these stakeholders was assembled using large language model platforms (ChatGPT and Microsoft Copilot) in parallel with expert knowledge from the authors (SI1, 2.2.2. and SI2, 2.2.). A total of 164 stakeholders were identified and of these, 62 were contacted representing a wide range of the value chain with questions regarding their knowledge about PFASs in their products or supply chain, availability of alternatives in these uses, and functions provided by both PFASs and substances other than PFASs. The e-mail campaign was conducted between November and December 2025, and a reminder was sent in January 2026.

A total of 17 responses were obtained, with semi-structured interviews conducted with representatives from 12 non-confidential stakeholders between November 2025 and March 2026. A summary of each interview was forwarded to the respondents, with the possibility to confirm or correct the content. We also obtained written statements and summarized interviews with respondents requesting to remain anonymous (SI1, 3.2.).

#### 2.2.3. Detection of PFASs by teardown-analysis studies

In teardown-analysis studies, researchers acquire commercial-scale batteries and tear them down into pieces, to analyze the batteries on the market and assess if laboratory-scale research is relevant for large-scale production. These studies provide insights into PFAS use in batteries on the market today.

In our work, we searched three databases (Web of Science, Scopus, and Scifinder) between December 2025 and February 2026 for relevant studies using the search string “teardown OR disassembly (Topic) and batter* (All Fields) and pouch OR cylindrical OR coin OR prismatic (All Fields)”, or similar, obtaining a total of 12 studies included in our work.^33–44^ (SI1, 2.2.3. and SI2, 2.3.). The results were presented in a summary table (SI1, 3.3.).

#### 2.2.4. Selected case studies: survey of the academic literature

To complement teardown studies, the academic literature was examined for information on PFAS use in batteries. It was beyond the scope of this work to perform this exercise for all qualified potential PFAS uses. Therefore, three cases were selected. Still, due to the large amount of literature studies, only review articles were included in our search as they were considered to provide a general assessment of the given field in a succinct manner.

The three selected cases were (1) cathode (of technical function: “binder”) in a Li-ion battery of LFP chemistry, (2) separator coating of Li-ion batteries with liquid electrolytes, and (3) lithium thionyl chloride (Li-SOCl_2_) battery cathode. For each selected case, the three above-mentioned databases were searched with a suitable search string, and the identified records were screened for relevance according to an adapted version of the PRISMA 2020 flowchart^45^ (SI1, 2.2.4. and SI2, 2.4.). For the Li-SOCl_2_ case, for which peer-reviewed literature was scarce, Google Scholar was also surveyed, also providing non-peer-reviewed reports. In total 25, 23, and 18 records, respectively, were screened as relevant, of which 21,^46–66^ 15,^67–81^ and 14 ^82–95^ records, respectively, were assessed to be eligible and included in our study (SI1, 3.4.).

#### 2.2.5. Potential alternatives as reported by Annex XV consultation comments

As noted above, the Annex XV public consultation not only requested information about uses of PFASs but also contained questions related to availability of alternatives. Thus, we collected any information related to alternatives from the Annex XV public consultation comments (SI1, 3.5.), and in the supporting evidence (SI1, 3.1.–3.4.).

#### 2.2.6. Integration of lines of evidence

In this work, we consider that information from different source types possesses equal weight, recognizing that they all have merits and shortcomings. For example, while previous use-mapping studies have aimed to identify a broad range of uses and alternatives, they are secondary sources and include all information found without a probability assessment or detailed performance assessment. In contrast, industry actors have primary information about substances, materials, designs, processes, and technology on the market, but this is mostly relevant for their own processes and limited with respect to that of others. In addition, they have an economic incentive to bring forward the information that would support conclusions that benefit their own products or, in the case of trade organizations, the interests of their members. The “real life” use of a substance, material, design, process, and technology is investigated in teardown-analysis studies, which provide up-to-date information about batteries in large-scale production. However, they only assess a small fraction of batteries on the market with a set of analytical limitations. Lastly, the academic literature could provide additional information about the function. Although the academic literature often focuses on novel materials that are not yet commercialized, the records also include some information on common uses (albeit at unspecified market volumes). Taken together, these source types complement each other in the evaluation of potential PFAS uses.

As a result, we collated all available information into lines of evidence for each of the listed potential PFAS uses (SI1, 4.). Based on the lines of evidence, we evaluated the likelihood of PFAS use on a 6-point scale: “1: very unlikely PFAS use”, “2: unlikely PFAS use”, “3: likely PFAS use, only for certain application(s) of use”, “4: likely PFAS use”, “5: very likely PFAS use”, and “6: lack of supporting evidence”. Rating 5 is only provided for cases where high market volumes can be (implicitly) confirmed. In our assessment, we consider that use rating 1 contradicts a PFAS use, while ratings 3–5 confirm a PFAS use. To account for potential uncertainties, we only consider strong evidence (*i.e.*, use rating 1) as a contradicted PFAS use even though use rating 2 could also indicate that PFASs are not used. Thus, both ratings 2 and 6 refer to potential PFAS uses that could not be confirmed or contradicted to a PFAS use with the available information. Overall, the evaluation is based on the weight of the evidence, where either increasing availability of information or information available from multiple source types further strengthens the evidence (either towards ratings 1 or 5).

We also assessed information about potential alternatives obtained both from the Annex XV consultation comments and the supporting evidence. Whereas a comprehensive alternatives assessment was beyond the scope of this work (in which the safety and sustainability of alternatives would also be assessed), we use the commercial availability of PFAS-free alternatives (*i.e.*, substances, materials, designs, processes and more) as a practical proxy for technical feasibility. The fact that it exists on the market means that it has achieved an acceptable level of performance for this given use. However, certain trade-offs may still be present limiting application in all uses, including changes in cycle life, fast-charging capability, low-temperature performance, electrochemical stability, or long-term durability. We assign an alternatives rating depending on the availability of potential alternatives: “1: potential alternatives available in large scale”, “2: several potential alternatives identified”, “3: potential alternatives identified”, “4: potential alternatives identified, only for certain application(s) of use” to “5: no potential alternatives identified”. Uses that were given an alternatives rating of “1: very unlikely PFAS use”, *i.e.*, a contradicted use in the use assessment, were provided the rating 1 in the assessment of potential alternatives. For uses where evidence was insufficient to confirm or contradict PFAS use, alternatives were not assessed and were assigned rating 6: availability of alternatives not investigated”. Through our approach, potential alternatives are identified, but their adoption on the market would require an evaluation of whether they are safer and more sustainable.

## 3. Results

### 3.1. Qualified potential PFAS uses in batteries

A total of 341 records were included in our database of PFASs in batteries as reported by Annex XV consultation comments (SI2, 3.1.). Fig. 4 illustrates these patterns by grouping records into higher-level categories. For example, nickel cadmium (NiCd) and nickel metal hydride (NiMH) battery technologies are combined as Ni-based batteries, while *e.g.*, magnesium, zinc-oxide, and lithium-air technologies are grouped under “Other battery technologies” (SI2, 3.2.). Because Battery Use level 2.2. (battery chemistry) was unknown for most records (247 records) and Battery Use level 2.3. (battery unit) overwhelmingly referred to the cell, these two dimensions are omitted from the flow diagram to maintain clarity. As it was not possible to find a grouping approach for end-use function and service function, these were excluded from the figure.

**Fig. 4:**
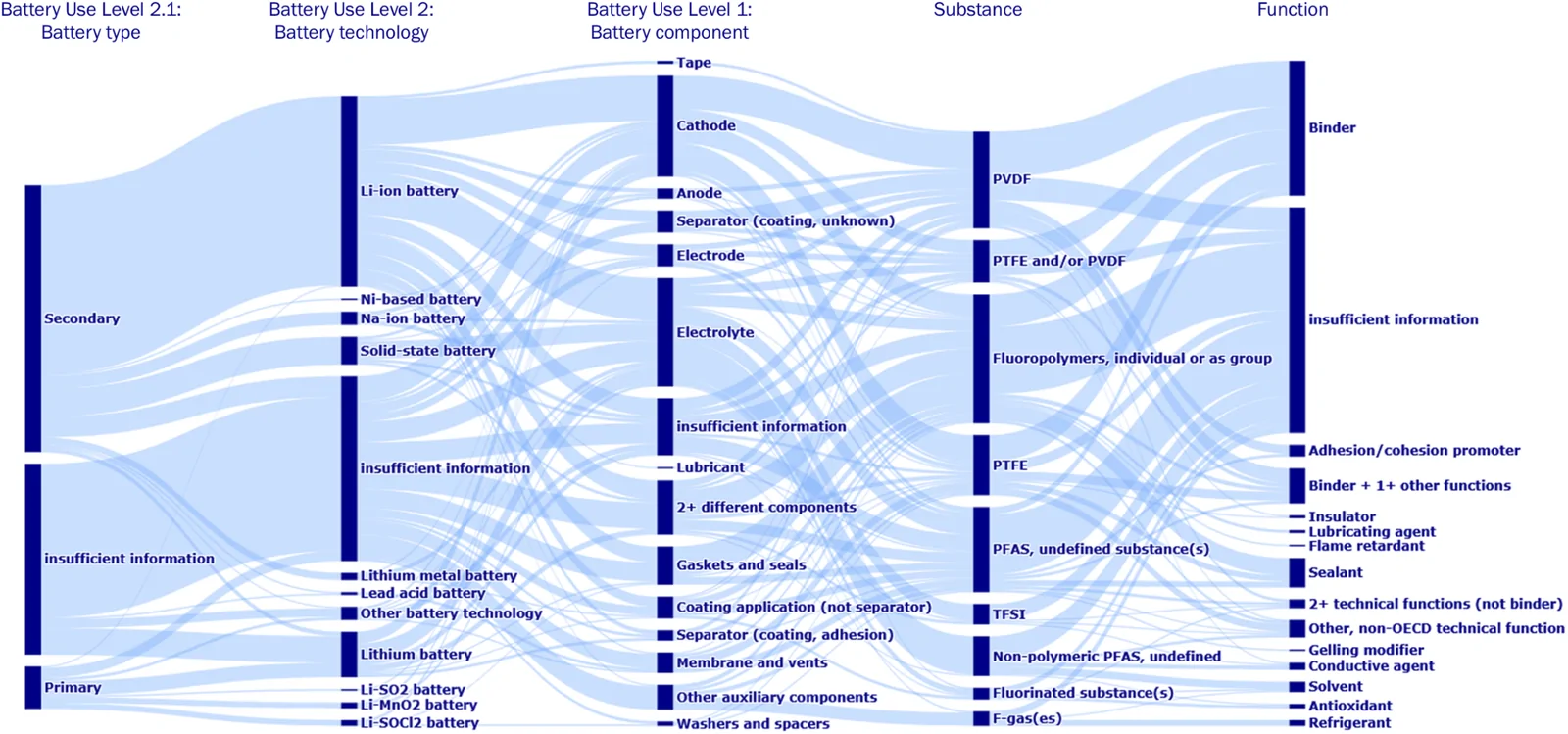
Flow diagram illustrating data from all records in the Battery Use & Function Framework database including selected levels. Order of Battery Use level 2.1. and 2 is switched for clarity of the figure. Note that the order of categories top-to-bottom has no meaning. An interactive diagram, showing the numbers of each category, is available in the .html file (SI3). The grouping of certain categories is detailed in SI2, 3.3.

Fig. 4 shows that most stakeholder-reported PFAS uses in batteries falls within secondary batteries (146 records), with Li-ion batteries dominating this group (112 records). At the component level (Battery Use level 1), the cathode is the most frequently mentioned component (70 records). Within these cathode-related entries, “binder” is the predominant technical function (63 records).

Fig. 4 also illustrates that reported PFAS uses in batteries cluster around several components in Li-ion batteries. While the potential PFAS uses identified in this study represent the cases with minimum information requirement fulfilled, their apparent frequency in the dataset is influenced by the level of detail provided in the Annex XV consultation comments. For example, “sealant” functions and the associated components “gaskets and seals” appear regularly in the records, but the information provided was too limited to qualify all but one of these entries to specific, well-defined potential PFAS uses that could be assessed further.

A total of 38 individual PFASs (including (co-)polymers) were included in the database (SI2, 3.3.). These included PVDF, entered as homopolymer in 118 records, and polytetrafluoroethylene (PTFE), included in 103 records, as well as several individual substances used mainly in the electrolyte such as salts of TFSI (16 as Li-salt, 1 as Na-salt, 2 as undefined), triflate (6 as Li-salt, 2 as undefined), and BETI (3 as Li-salt). Among the groups of substances, PVDF co-polymers or “fluoropolymers” were most often noted, apart from the undefined “PFAS”. Other examples of groups included fluororesins, fluoroelastomers, fluorinated gases or more specified such as “perfluorinated tertiary alcohols and their aluminium derivatives”, “ionic fluoro fluids (*e.g.*, fluorohydrogenate ionic liquids)” and “hexafluoroisopropoxy esters (*e.g.*, hexafluoroisopropanol HFIP).” Fluorinated substances other than PFASs that were brought forward by the stakeholders as PFASs included LiPF_6_, LiFSI and PVF.

Only a subset of records included information on function. Of the total 341 records, 187 had information about the technical function. Most records listed one technical function (155, of which binder was included in 92). Two technical functions were noted in 18 cases (with binder listed in 10 records), while binder was part of every record with 3 technical functions (10 records) as well as 4 technical functions (4 records). When more than one technical function was listed, it could refer both to one substance (*e.g.*, “PVDF”) or several (*e.g.*, “PTFE; ETFE; FEP; PVDF”, “fluororesin” or “PFAS”). Other technical functions included, *e.g.*, sealing (barrier), dispersing agent, and refrigerant. Some functions were linked to the manufacturing of battery cells (denoted with “(in processing)” in the database), such as PVDF being a dispersing agent during wet processing of Li-ion cathode production. A total of 67 and 71 records included information on the end-use function and service function, respectively, while only 33 records included details of technical requirements.

Each record was assessed for the sufficiency of information to be qualified as a potential PFAS use according to the decision-making model (Fig. 3). 78 records were compiled into a total of 36 individual potential PFAS uses in batteries (Table 4). Thus, of the 341 records extracted from the Annex XV consultation comments, only 23% consisted of sufficient information to qualify as potential PFAS uses. The remaining records either contained several entries (*e.g.*, information about more than one battery technology and/or component) or lacked essential information on Battery Use sub-levels 2.1. through 2.3. As it is only Battery Use levels 1 and 2 (including sub-levels) that are assessed for sufficient information, other information did not affect qualification as a potential PFAS use and several of the records which contained similar information, *e.g.*, related to Battery Use level 3, substance(s) or function(s), were aggregated.

**Table 4:** List of potential PFAS uses in batteries. Potential PFAS uses no. 1–5 refer to primary batteries, while the remaining refer to secondary batteries

Potential PFAS use no.	Battery Use level 2: battery technology	Relevantsub-use level(s)^a^	Battery Use level 1: battery component	Databaserecord no.^b^
1	Li-MnO_2_ battery	Li-MnO_2_ chemistry	Cathode	22, 169
2	Li-MnO_2_ battery	Liquid electrolyte	Electrolyte	39, 40, 94
3	Li-SOCl_2_ battery	Li-SOCl_2_ chemistry	Cathode	20, 93
4	Li-SO_2_ battery	Li-SO_2_ chemistry	Cathode	21
5	AgO_2_ battery	AgO_2_ chemistry	Cathode	25
6	Li-ion battery	LFP chemistry	Cathode	106, 120, 182, 190, 234
7	Li-ion battery	LCO chemistry	Cathode	114, 190
8	Li-ion battery	LMO chemistry	Cathode	182, 190
9	Li-ion battery	NMC chemistry	Cathode	120, 182, 190, 234
10	Li-ion battery	NCA chemistry	Cathode	182, 190
11	Li-ion battery	Metal oxide chemistries	Cathode, coating	340
12	Li-ion battery	Graphite anode in LFP, LMO, NMC, and NCA chemistries	Anode	183
13	Li-ion battery	NTO anode in LTO chemistry	Anode	12
14	Li-ion battery	Liquid electrolyte	Electrolyte	28, 29, 30, 31, 32, 88, 119, 137, 159, 179, 209, 213, 221, 228, 240, 271, 278, 294, 301, 314, 316, 339, 341
15	Li-ion battery	Polymer electrolyte	Electrolyte	33, 222, 223
16	Li-ion battery	Liquid electrolyte	Separator	86, 185, 204, 216, 230, 255, 256, 266, 255
17	Li-ion battery	Liquid electrolyte	Separator, coating	50, 55, 116, 121, 136, 202, 295
18	Li-ion battery	Cell, sub-assembly	Lubricant	164
19	Li-ion battery	Cell	Seal gasket	197, 203
20	Li-ion battery	Pack, outer packaging	Vent	132
21	Na-ion battery	Layered metal oxide chemistries	Cathode	15, 83
22	Na-ion battery	PBA chemistry	Cathode	15
23	Na-ion battery	Phosphate-based chemistry	Cathode	15
24	Na-ion battery	Hard carbon anode in PBA chemistry	Anode	16
25	Na-ion battery	Liquid electrolyte	Electrolyte	34
26	Na-ion battery	Liquid electrolyte	Separator	218
27	Lithium metal battery	LFP chemistry	Cathode	26
28	Lithium metal battery	LCO chemistry	Cathode	26
29	Lithium metal battery	LMO chemistry	Cathode	26
30	Lithium metal battery	NMC chemistry	Cathode	26
31	Lithium metal battery	NCA chemistry	Cathode	26
32	Lithium metal battery	Polymer electrolyte	Electrolyte	325
33	Solid-state battery	Solid electrolyte	Electrolyte	2, 155, 323
34	Solid-state battery	Gel electrolyte	Electrolyte	3, 155
35	Solid-state battery	Polymer electrolyte	Electrolyte	124, 125, 237
36	Lead acid battery	BMS, catalytic device	Vent	131

a The use was defined according to the decision-making model outlined in Fig. 3. List of abbreviations: lithium manganese oxide (Li-MnO_2_), lithium sulfur dioxide (Li-SO_2_), lithium thionyl chloride (Li-SOCl_2_), silver oxide (AgO_2_), lithium iron phosphate (LFP), lithium cobalt oxide (LCO), lithium manganese oxide (LMO), lithium nickel manganese cobalt (NMC), lithium nickel manganese aluminum oxide (NCA), niobium titanate oxide (NTO), lithium titanate oxide (LTO), Prussian blue analogue (PBA), and battery management system (BMS).

b Database records are outlined in SI2, 3.1.

### 3.2. Cross-technology assessment

A total of 16 PFAS uses could be confirmed with supporting evidence (use rating 3–5), including two cases where PFAS use appears likely in some applications (use rating 3) (Fig. 5a), *e.g.*, Li-ion/LFP chemistry/Cathode and Li-ion/Liquid electrolyte/Electrolyte, respectively. On the other hand, two uses were contradicted (Li-ion/Graphite anode in LFP, LMO, NMC, and NCA chemistries/Anode and Li-ion/Liquid electrolyte/Separator). For some potential PFAS uses, the low availability of relevant information resulted in the need for extrapolation. For example, while PFAS use in Li-ion/NMC chemistry/Cathode is considered “very likely,” the extrapolation led to a conservative use rating of “likely” rather than “very likely” for Lithium metal/NMC chemistry/Cathode. For the remaining 18 uses, supporting evidence was not available to confirm or contradict the potential PFAS use, including one potential PFAS use (Na-ion/Liquid electrolyte/Separator) with use rating 2. This group includes most of the potential PFAS uses in a primary battery as well as all potential PFAS uses in a solid-state battery.

**Fig. 5:**
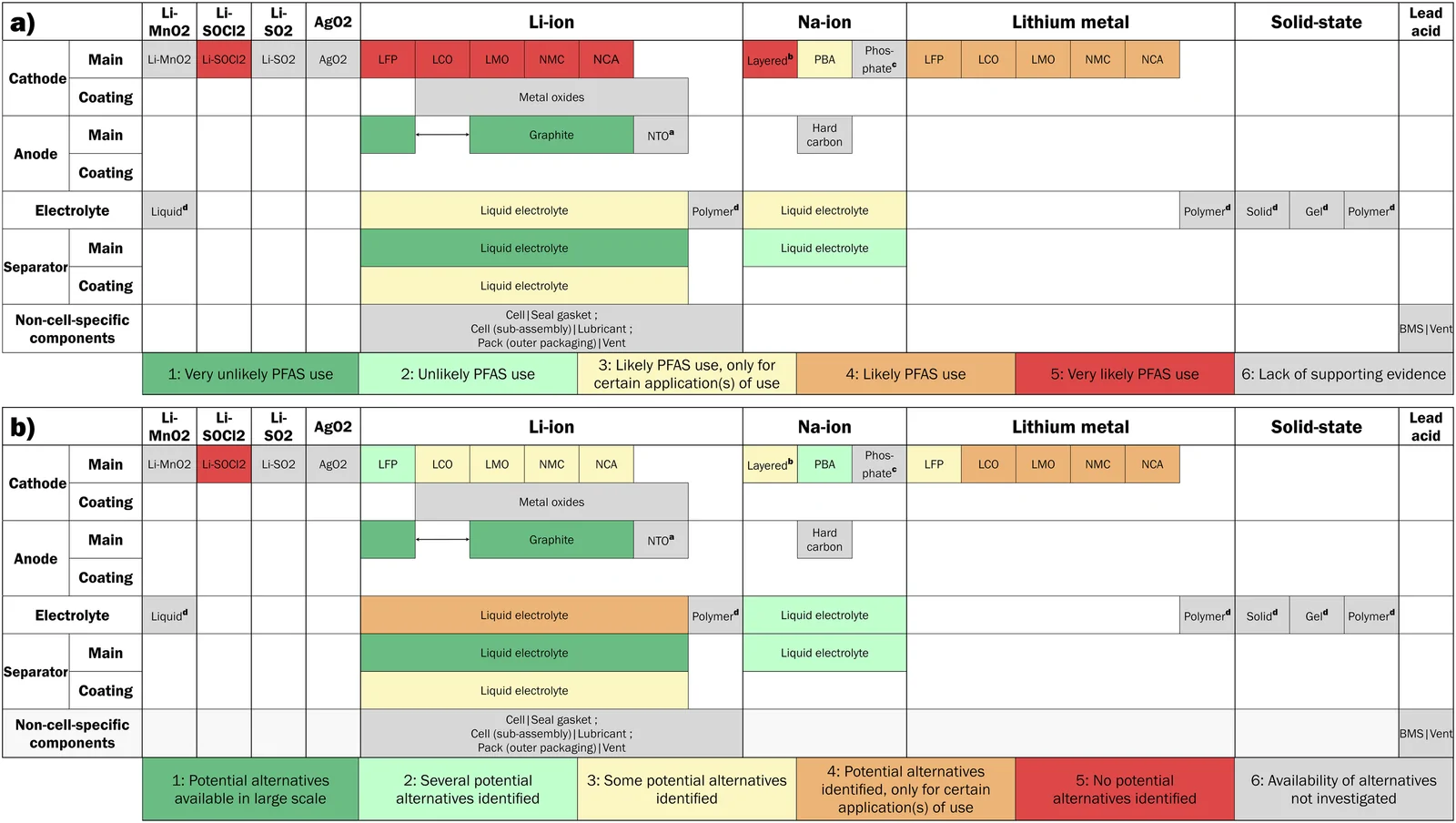
(a) Evaluation of the likelihood of PFAS presence in the 36 qualified potential PFAS uses on the market; and (b) evaluation of availability of potential alternatives (PFAS-free substance, material, design, process and technology on the market) in listed potential PFAS uses. The color scale follows the traffic-light convention and indicates (a) the likelihood of PFAS use, with dark red denoting very likely use and dark green denoting very unlikely use, and (b) availability of alternatives with dark red denoting no potential alternatives identified and dark green denoting potential alternatives available on a large scale. Both gray and white indicate lack of data, where the latter would relate to lack of supporting evidence. Primary batteries include lithium manganese oxide (Li-MnO_2_), lithium sulfur dioxide (Li-SO_2_), lithium thionyl chloride (Li-SOCl_2_) and silver oxide (AgO_2_). Li-ion and lithium metal cathode chemistry abbreviations are as follows: lithium iron phosphate (LiFePO_4_, LFP), lithium cobalt oxide (LiCoO_2_, LCO), lithium manganese oxide (LiMnO_2_, LMO), lithium nickel manganese cobalt oxide (LiNi_1−*x*−*y*_Mn_*x*_Co_*y*_O_2_, NMC), and lithium nickel cobalt aluminum (LiNi_1−*x*−*y*_Co_*x*_Al_*y*_O_2_, NCA). Na-ion battery chemistry includes Prussian Blue Analogues (PBAs). Non-cell-specific components are grouped and represent a total of 4 potential PFAS uses. Asterisk included for abbreviations as follows: ^a^NTO in LTO chemistry, ^b^layered metal oxide chemistry, ^c^phosphate-based chemistries, and ^d^referring to electrolytes in the liquid, polymer or solid phase.

In the evaluation of availability of potential alternatives (Fig. 5b), most supporting evidence was available on potential PFAS uses related to Li-ion batteries and Na-ion batteries. Meanwhile, the limited evidence available for Li-SOCl_2_ batteries did not provide any information regarding the availability of alternatives, thus this was provided an alternatives rating of “No potential alternatives identified”.

#### 3.2.1. Primary batteries (potential PFAS uses no. 1–5)

Listed potential PFAS uses in primary batteries include uses in lithium manganese oxide (Li-MnO_2_), lithium sulfur dioxide (Li-SO_2_), lithium thionyl chloride (Li-SOCl_2_), and silver oxide (AgO_2_) battery technologies (SI1, 4.1.–4.4.). Overall, there was a lack of supporting evidence to confirm or contradict these potential PFAS uses, but when selecting one of them in the case studies (Li-SOCl_2_), we could identify that the cathode most often contains PTFE as a binder (SI1, 4.2.). For this confirmed PFAS use, there was no available information about potential alternatives. It was not possible to extrapolate results concerning one use to another for primary batteries.

#### 3.2.2. Li-ion batteries (potential PFAS uses no. 6–20)

A total of 15 potential PFAS uses were listed for Li-ion batteries (SI1, 4.5.). More than half of the potential PFAS uses were either confirmed (7) or contradicted (2) through evaluation of supporting evidence. We conclude that it is very likely that the cathode is a component across the PFAS uses involving Li-ion battery chemistry (technical function: “binder”; PVDF or its copolymers).

We identified several available alternatives for the components in Li-ion batteries. First, we conclude there are more available alternatives for the potential PFAS use no. 6 (LFP chemistry) compared to the other confirmed cathode chemistries (SI1, 4.5.1.). The survey of the academic literature and interviews with battery stakeholders indicate that the LFP chemistry is not sensitive to water (as is NMC and other layered metal oxides), and water-based processing is possible, indicating a large range of potential alternatives fulfilling the technical function “binder”. The teardown-analysis studies and input from battery stakeholders showcased that there are several potential market-ready alternatives with binding functionality (*i.e.*, avoiding PVDF) in LFP batteries. However, for potential PFAS uses no. 7–10 (NMC chemistry and other metal oxides), the chemistry limits the direct adoption of water-based processes. Nevertheless, non-water-based PFAS-free alternatives have been showcased on the market, such as by Ateios, Leclanché and GRST, indicating several potential alternatives for these PFAS uses also.^96–98^

Second, we conclude that it is very likely that the anode binder is of a material other than PFASs on the market today, contradicting the potential PFAS use no. 12 (SI1, 4.5.2.). Although previous use-mapping studies mentioned the use of a PFAS-based binder in Li-ion battery anodes, evidence from teardown-analysis studies, other academic literature, and the lack of information from battery stakeholders, such as downstream users all suggest that this could be considered as a confirmed “non-PFAS use”.^99,100^ Thus, alternatives (*i.e.* what is already the primary choice in commercial applications) include mainly a mix of styrene-butadiene rubber (SBR) and carboxymethyl cellulose (CMC) with material providers including Ashland and Eneos Materials.^101,102^

Third, we conclude based on the supporting evidence that it is very likely that the liquid electrolyte in Li-ion batteries on the market contains a fluorine-based lithium salt (LiPF_6_) as the main salt (SI1, 4.5.3.). Additives are included in electrolyte formulations to influence its function(s), but the compositions are proprietary information. Therefore, it is not possible to confirm the potential PFAS use no. 14 (Li-ion/Liquid electrolyte/Electrolyte) with high confidence. Still, based on available information from E-Lyte and RECHARGE, LiPF_6_ would often be sufficient to provide the necessary function(s) of the electrolyte in cost-driven battery applications, while they report that in high-performance applications, particularly with requirements of thermal stability, PFAS additives are adopted.^103,104^ Thus, we provide the use rating 3 (“Likely PFAS use, only for certain application(s) of use”) for potential PFAS use no. 14.

Lastly, the supporting evidence provided extensive information related to potential PFAS uses no. 16 and 17 (the Li-ion battery separator and its coating) (SI1, 4.5.4.). We conclude that it is very likely that the separator is based on a PE and/or PP material, based on available information from the academic literature (teardown-analysis studies and literature reviews) and industry representatives. While the academic literature included as supporting evidence discusses the use of polymer electrolytes, possibly PFAS-based, as a separator type, potential PFAS uses no. 16 and 17 refer to liquid electrolytes specifically. The common separator materials (*i.e.*, PE and/or PP) provide sufficient mechanical strength and electrolyte compatibility at a low cost. As a result, the potential PFAS use no. 16 was contradicted, yielding another case of confirmed “non-PFAS use”.

However, for high-performance batteries, the thermal stability provided by PE and PP is reported to not be sufficient, which may be addressed by coating the base film (most often these microporous membranes with ceramic particle coating). The trade-offs of higher economic costs and certain performance disadvantages are in these cases reportedly weighed against higher thermal stability. PFASs in the separator coating were described with either “binder” or “adhesion/cohesion promoter” function, where the former refers to binding of the ceramic particles to the base film and the latter a layer between the separator and the cathode. Whereas ceramic-coated separators are reported in the teardown-analysis studies and identified by other academic literature, they lack information on the function provided by the material (“binder” or “adhesion/cohesion promoter”).

There are several potential alternatives to separator coating with “binder” function described in the supporting evidence. Ashland and KURZ provide PFAS-free binders for this use,^101,105^ either based on polyvinylpyrrolidone (PVP) or poly(methyl methacrylate) PMMA, respectively. Furthermore, several alternatives providing the “binder” function in separator coatings are reported in the academic literature. For example, Luiso and Fedkiw (2020) noted that “it is reported that the Sumitomo separator used by Panasonic and Tesla Motors involves a coating with ceramic particles and an aromatic polyamide to increase the penetration strength”.^75^

On the other hand, the “adhesion/cohesion promoter” function between the cathode and separator is applied during battery production. In certain applications, this is obtained *via* a layer between the cathode and separator. While Leclanché reports the use of PVDF in their processes, it is not clear to which extent PVDF is used as a separator coating.^98^ KURZ provides a potential alternative technology to PVDF in the adhesion coating. In addition, they state that in cylindrical cells or in lamination processes, PVDF might not be required.^105^ We note that quantitative technical requirements were not provided by battery stakeholders or in any of the supporting evidence, and that the assessment by battery stakeholders resulting in the conclusion of a material being “required” is an assessment by single companies. Our assessment of alternatives would not exclude that PFAS-based separator(s) (coatings) may be used on the market; instead, we point to the availability of alternatives fulfilling the technical function of interest.

In sum, we conclude that several potential alternatives are identified for the potential PFAS use no. 17 providing the function “binder”, while it is unclear to what extent alternatives fulfill the function “adhesion/cohesion promoter”. Thus, this use is concluded to have alternatives rating 3 (“Some potential alternatives identified”).

#### 3.2.3. Na-ion batteries (potential PFAS uses no. 21–26)

A total of six potential PFAS uses were listed for Na-ion batteries, of which four could be confirmed (SI1, 4.6.).

Using the limited evidence for Na-ion batteries together with extrapolation of the results from Li-ion batteries, we conclude that it is very likely that the cathode of Na-ion batteries with layered metal oxide chemistry (potential PFAS use no. 21) constitutes a PFAS use (use rating 5) with few potential alternatives identified (alternatives rating 3) fulfilling the technical function “binder” (SI1, 4.6.1.). A use rating 3 was concluded for the PBA chemistry (potential PFAS use no. 22) and, because it is not sensitive to water, several potential alternatives were identified for this use (alternatives rating 2). Thus, an alternative technology to a Na-ion battery with layered metal oxide chemistry could be a Na-ion battery with PBA chemistry, considering the similarities on Battery Use level 3. This may entail adjustments to battery production lines, but no technical barriers for this change were identified in our study.

We also conclude that it is likely that the main components of the electrolyte (potential PFAS use no. 25) are substances other than PFASs, such as NaPF_6_ and non-fluorinated solvents (SI1, 4.6.3.). However, one Annex XV consultation comment reported the use of NaTFSI. As with the corresponding use in Li-ion batteries (potential PFAS use no. 14), it is likely that the TFSI-salt is not included in electrolytes when cost is considered higher priority than technical performance by battery manufacturers. This effect should be even more prevalent for Na-ion batteries because they are not used in high-performance applications to the same extent as Li-ion batteries. As a result, this potential PFAS use was provided with use rating 3 and alternatives rating 2.

We found contradicting evidence of the possibility of extrapolating the conclusions for Li-ion batteries (potential PFAS use no. 16) to the corresponding use of separators in Na-ion batteries (potential PFAS use no. 26) (SI1, 4.6.4.). There was no information about PFAS-based separators in Na-ion batteries in the supporting evidence. Thus, we conclude that it is unlikely that the separator is a PFAS use (use rating 2) and, conversely, that several potential alternatives were identified for this use (alternatives rating 2).

#### 3.2.4. Lithium metal and solid-state batteries (potential PFAS uses no. 27–35)

A total of nine potential PFAS uses were connected either to the lithium metal and solid-state batteries (SI, 4.7). As these uses refer to a specific choice of anode (lithium metal) (potential PFAS uses no. 27–31) or (solid-state) electrolyte (potential PFAS use no. 33–35), there also exists an overlap where one potential PFAS use was linked to both, *i.e.*, the polymer electrolyte in a lithium metal battery (potential PFAS use no. 32).

There was limited information on lithium metal batteries (SI1, 4.7.1.). However, the battery chemistries are similar to those of Li-ion (LFP, LCO, LMO, NMC and NCA), and thus, we considered it possible to extrapolate the conclusions from Li-ion battery cathodes (potential PFAS uses no. 6–10) to these five potential uses in lithium metal batteries (potential PFAS uses no. 27–31). As a result, we consider it likely that these are PFAS uses (use rating 4) with available alternatives. As with Li-ion batteries, the LFP chemistry is not water sensitive and has a higher rating of availability of alternatives (alternatives rating 3) compared to the other chemistries (alternatives rating 4).

Overall, for the remaining potential PFAS uses in lithium metal and solid-state batteries, there was limited information, not sufficient to confirm or contradict the remaining listed potential PFAS uses (use rating 6).

#### 3.2.5. Lead acid batteries (potential PFAS use no. 36)

For lead acid batteries there was one potential PFAS use listed, namely that of the BMS catalytic device vent (potential PFAS use no. 36) (SI1, 4.8.). Although many stakeholders in the Annex XV public consultation reported negative results for use of PFASs in lead acid batteries (*i.e.* that they do not contain PFASs), there was a lack of data related to Battery Use level 2.3. (battery unit). Overall, there was a lack of information on this potential PFAS use, and we conclude that we can neither confirm nor contradict (use rating 6) this potential PFAS use.

### 3.3. PFAS function and potential alternatives in confirmed uses

Based on the above evaluation, we confirmed 16 and contradicted two PFAS uses in batteries. We summarize this in Table 5 together with the associated substances and technical functions as reported by the Annex XV consultation comments. We also include information on potential alternatives, separated into substance(s) and material(s) or design(s), and process(es) already available on the market. Alternative technologies compared by grouping several uses together are not included in the table. However, we note that collectively, all potential PFAS uses in Na-ion batteries have available alternatives (*i.e.* all Na-ion battery components can be produced without PFASs). Therefore, as Na-ion batteries can provide the same service function (*i.e.* are used in similar battery applications (Battery Use level 3), a potential alternative to Li-ion batteries may be Na-ion batteries for some applications where Na-ion batteries are comparable to Li-ion batteries. However, the commercial feasibility of substitution on the technology level would need further investigation.

**Table 5:** All potential PFAS uses as described by Annex XV consultation comments which we confirmed (use rating 3–5; 16 uses) or contradicted (use rating 1; 2 uses)

Potential PFAS use no.	Use rating	Battery Use level 2: battery technology	Relevant sub-use level^a^	Battery Use level 1: component	Potential substances (within PFAS def.)^b^	Potential technical function(s)^b^	Potential alternative substance(s)/material(s)^c^	Potential alternative design(s)/process(es)^c^
3	5	Li-SOCl_2_ battery	Li-SOCl_2_	Cathode	PTFE	Binder, lubricating agent (in processing), and other (“low water absorption during mixing”)	No potential alternatives identified	
6	5	Li-ion battery	LFP	Cathode	PVDF and PTFE	Binder, dispersion agent (in processing), corrosion inhibitor, viscosity modifier (in processing), and adhesion/cohesion promoter	Water-based (*e.g.*, CMC and PVA)	Ateios, GRST, and other water-based manufacturing
7	5	Li-ion battery	LCO	Cathode	PVDF	Binder, adhesion/cohesion promoter, dispersive agent (during processing), corrosion inhibitor, and viscosity modifier (in processing)	Acrylic acid-based	Ateios and GRST
8	5	Li-ion battery	LMO	Cathode	PVDF and PTFE	Binder, dispersion agent (in processing), corrosion inhibitor, and viscosity modifier (in processing)	Acrylic acid-based	Not identified
9	5	Li-ion battery	NMC	Cathode	PVDF and PTFE	Binder, dispersion agent (in processing), corrosion inhibitor, and viscosity modifier (in processing)	Acrylic acid-based	Ateios, GRST (water-based: up to 60% Ni, NMP-based: up to 80% Ni), and Leclanché (up to 80% Ni)
10	5	Li-ion battery	NCA	Cathode	PVDF and PTFE	Binder, dispersion agent (in processing), corrosion inhibitor, and viscosity modifier (in processing)	Acrylic acid-based	GRST
12	1	Li-ion battery	Graphite anode in LFP, LMO, NMC, and NCA chemistry	Anode	PTFE	Binder	Use contradicted, often combinations of CMC/SBR, provided *e.g.*, by Ashland and Eneos Materials	
14	3	Li-ion battery	Liquid electrolyte	Electrolyte	LiTFSI, PTFE; FEP, PFAS (low molecular), hydrofluoroether, 1,1,2,2-tetrafluoroethyl 2,2,3,3-tetrafluoropropyl ether (D2), 1,3-dioxolan-2-one, 4-(2,2,3,3,3-pentafluoropropyl), 2,2,2-trifluoroethyl methyl carbonate, 1,1,2,2-tetrafluoroethyl 2,2,3,3-tetrafluoropropyl ether, hexafluoroisopropoxy ester *e.g.* hexafluoroisopropanol HFIP, LiCTFSI, lithium 4,4,5,5-tetrafluoro-1,3,2-dithiazolidine-1,1,3,3-tetraoxide, fluorine based, LiBETI, tris(2,2,2-trifluoroethyl)borate, trifluorotoluene, perfluorinated tertiary alcohols and their aluminum derivatives, F-EPE	Solvent, antioxidant, solvent, other (allow exchange of ions between electrodes), antioxidant, corrosion inhibitor, and other (“suppress hf generation”), conductive agent	Fluorinated substances (not within PFAS definition) used as main salts in electrolyte and fulfill desired functions unless in high-temperature applications	E-Lyte formulations
16	1	Li-ion battery	Liquid electrolyte	Separator	PVDF, PTFE; VdF/HFP; VT475; VW770, and fluoroelastomer	Adhesion/cohesion promoter	Use contradicted, combinations often PP/PE combinations, provided *e.g.*, by Celanese	
17	3	Li-ion battery	Liquid electrolyte	Separator, coating	PVDF, PTFE; FEP, and PVDF (Kynar® FSF®)	Binder, adhesion/cohesion promoter, insulator, and other (allow exchange of ions between electrodes)	Binder: poly(acrylate)-based, aromatic polyamide	KURZ, Ashland, Panasonic batteries
							Adhesion/cohesion promoter: PMMA	Tesla Motors batteries, cylindrical batteries, and lamination processes
21	5	Na-ion battery	Layered metal oxides	Cathode	PVDF	Binder, dispersion agent, corrosion inhibitor, and viscosity modifier	(Extrapolated from Li-ion/LCO, LMO, NMC or NCA chemistries/Cathode)	
22	3	Na-ion battery	PBA	Cathode	PVDF	Binder, dispersion agent, corrosion inhibitor, and viscosity modifier	Water-based (*e.g.*, CMC, SBR, and carbon)	Not identified
25	3	Na-ion battery	Liquid electrolyte	Electrolyte	NaTFSI	No information	(Extrapolated from Li-ion/Liquid electrolyte/Electrolyte)	
27	4	Lithium metal battery	LFP	Cathode	PVDF, PTFE	Binder	(Extrapolated from Li-ion/LFP chemistry/Cathode)	
28	4	Lithium metal battery	LCO	Cathode	PVDF, PTFE	Binder	(Extrapolated from Li-ion/LCO chemistry/Cathode)	
29	4	Lithium metal battery	LMO	Cathode	PVDF, PTFE	Binder	(Extrapolated from Li-ion/LMO chemistry/Cathode)	
30	4	Lithium metal battery	NMC	Cathode	PVDF, PTFE	Binder	(Extrapolated from Li-ion/NMC chemistry/Cathode)	
31	4	Lithium metal battery	NCA	Cathode	PVDF, PTFE	Binder	(Extrapolated from Li-ion/NCA chemistry/Cathode)	

a The use was defined according to the decision-making model outlined in Fig. 3.

b Substance identity and technical functions were not assessed with supporting evidence but obtained directly from Annex XV consultation comments.

c We also list potential alternatives, described according to the conclusions in this study. Potential alternatives are divided into either as substance(s)/material(s) or design(s)/process(es). Potential alternatives are listed as the former (second to last column), the latter (last column), or as both (spanning both second to last and last column). List of abbreviations: lithium thionyl chloride (Li-SOCl_2_), lithium iron phosphate (LFP), lithium cobalt oxide (LCO), lithium manganese oxide (LMO), lithium nickel manganese cobalt (NMC), lithium nickel cobalt aluminum oxide (NCA), Prussian blue analogue (PBA), styrene-butadiene rubber (SBR), carboxymethyl cellulose (CMC), poly(vinyl alcohol) (PVA), poly(methyl methacrylate) (PMMA), polyethylene (PE), polypropylene (PP) and *n*-methyl-2-pyrrolidone (NMP). A list of potential substances is provided in SI2, 3.3.

Alternatives listed in Table 5 include only those that can be related to specific uses. More general information about potential alternatives can be found in the previous use-mapping studies from ChemSec, Danish Environment Protection Agency, and Savvidou *et al.* (2025)^12,25,29^ (SI1, 3.1.) as well as by battery stakeholders (SI1, 3.2.). Identified potential alternatives were also listed in the Background Document^2^ and reported by Annex XV consultation comments (see *e.g.*, Table S9 in SI1, 3.5.).

## 4. Discussion

We combined the Annex XV consultation comments with supporting evidence allowing us to move from single-company claims to a broader market-level evaluation of potential PFAS uses in batteries (Fig. 5a). Whereas a full alternatives assessment was outside the scope of this study, the availability of potential PFAS-free alternatives (substances, materials, designs, processes, and technologies) on the market was considered as a practical proxy for technical feasibility across battery components and technologies (Fig. 5b). We did not evaluate whether these alternatives are less harmful than PFASs; such considerations fall within a full alternatives assessment. This cross-technology perspective shows that technical feasibility can already be achieved with potential alternatives for all but one confirmed use (potential PFAS use 3, see Table 5). Four potential PFAS uses have several potential alternatives (alternatives rating 2) already on the market, while alternatives were identified only in certain applications for five confirmed uses (alternatives rating 4). Examples of the latter include Li-ion batteries with liquid electrolytes with PFAS-based salts such as LiTFSI in high temperature or high-voltage applications. In addition, two potential uses were contradicted in the weight-of-evidence assessment (use rating 1).

The distribution of use ratings and alternative ratings indicates that near-future PFAS use in batteries is limited only to a small subset of applications, while most qualified PFAS uses already have viable PFAS-free alternatives (based on technical feasibility). Our objective was not to identify battery technologies that are already entirely PFAS-free. Rather, we adopted a cross-technology, component-level approach, examining whether PFAS-free alternatives exist for individual battery components. This enabled us to demonstrate that several battery technologies could potentially be made PFAS-free by combining alternative components that are already commercially available. The full commercial feasibility of such combinations, however, remains to be demonstrated.

In short, our methodology excludes comments stating “there are no alternatives” when they lack details on (1) which uses were assessed, (2) which function(s) is/are provided, and (3) which PFAS(s) and alternative(s) were assessed. For example, several comments stated that PVDF is a binder in electrodes, without further detail about which electrode and the description of “lithium batteries” could refer either to primary or secondary batteries. In addition to detailed use and function level information (which we conceptualized with the Battery Use & Function Framework), substance-level information is critical. For example, two stakeholders listed the salt LiPF_6_ together with LiTFSI, implying that the former is a PFAS. Similarly, some F-gases used in battery cooling systems may not be PFASs. When stakeholders list an undefined PFAS, it is not possible to critically assess whether they refer to a fluorinated substance within or outside the PFAS definition.

While some battery manufacturers may have investigated alternatives and not found technically and economically feasible options within their product lines, we note that this conclusion reflects internal R&D capacity at a specific point in time. Battery manufacturers have many options to improve performance, selecting between different materials (related to both electroactive and inactive components), design (of each component, and the configuration or integration into the end-use product), and software controls (*e.g.*, programming the BMS), among other aspects. However, such adjustments are often neither quick nor straightforward to implement, making it difficult for manufacturers to respond rapidly to shifts in market conditions. A company's inability to identify alternatives does not imply that alternatives are non-existent, but rather reflects the constraints of its own designs, investments, and development timelines. Interviews conducted for this work indicated that alternatives already on the market could be scaled up within 1–8 years^97,98,104^ (SI1, 3.2.). A detailed component- and chemistry-specific cross-technology assessment should be presented by those claiming that alternatives do not exist because technical functions vary, and companies may reach different conclusions, leading to contradictory public statements about transition timelines.

In this study we have adopted “availability on the market” as a proxy for technical feasibility, recognizing that a full economic assessment was outside the scope of this work. Briefly, several factors can contribute to lock-ins that delay the uptake of alternatives even when technically feasible options exist. These include long-term capital investments in manufacturing infrastructure (for example, plants designed for wet-cathode processing using PVDF and *n*-methyl-2-pyrrolidone (NMP)), certification of materials for certain uses, supply-chain stability as well as legal or contractual constraints such as supplier agreements or standards. Therefore, scaling up until the market is fully transitioned to PFAS-free alternatives involves more than just technical feasibility; it requires addressing the systemic factors that maintain existing lock-ins and while economic factors are not permanent they reflect current reality. Technical feasibility and the practical ability to transition are therefore closely connected and change over time. Although this work does not directly assess economic feasibility, we recognize that companies already marketing PFAS-free battery materials demonstrate that substitution is technically and economically possible for at least these given uses.

### 4.1. Our added contribution

The present study contributes to the broader context of PFAS use in batteries. Previous literature has often listed PFAS uses in batteries without distinguishing between confirmed uses, historical uses, and uses limited to academic research. This has led to repeated claims that are not always reflecting market reality. In our work, PFAS uses in batteries refer primarily to articles that are already on the market, provided by several battery manufacturers, mirroring our definition of a PFAS-free alternative, which requires the alternative to be available on the market. By applying a structured framework and a weight-of-evidence approach, our results clarify where PFASs are actually used (“confirmed”; use ratings 3–5), where they are not (“contradicted”; use rating 1), and where the evidence remains uncertain (“lack of evidence”; use rating 2 and 6). This provides a more accurate basis for discussions about essential use, substitution potential, and regulatory design. In several cases, our findings diverge from existing literature. These differences highlight the significance of the present work in correcting misconceptions and providing a more reliable foundation for future assessments.

Of the 20 previous use-mapping studies we identified, several briefly reported on PFAS uses in batteries.^19–22,26,30,31^ These studies provide substantial background information, but our work goes beyond reporting PFASs in batteries. For example, we conclude that the Li-ion battery anode is not a PFAS use, consistent with the US DOE report describing PTFE as a historical anode binder, but contradicting Glüge *et al.* (2020) and Ebnesajjad (2021).^6,22,24^ Rensmo *et al.* (2023) described many fluorinated substances used in Li-ion electrolytes, but comparison with Annex XV consultation comments suggests that many are limited to academic research and not relevant for the market.^11^ Figuière *et al.* (2024) described lead-acid batteries or solid-state batteries as alternatives to Li-ion batteries, but the former are not comparable due to use in different applications, and the latter likely make up several PFAS uses.^7^

These above examples illustrate how incorporating multiple lines of evidence ensures data quality. In this work, instead of grouping uses, functions, and substances as described by stakeholders in the Annex XV public consultation, we separate potential PFAS uses based on information availability and then assess their likelihood. That only 78 of 341 records contained sufficient information to be considered potential PFAS uses demonstrates that consultation submissions often lacked necessary detail for complex use cases such as batteries. Second, we incorporate extensive supporting evidence, including input from the battery stakeholders and the academic literature, presented transparently in SI, Section 3. We show that some potential PFAS uses can be contradicted by other evidence, indicating that previous use-mapping studies may have overstated PFAS use in batteries.

Only the Annex XV report and the Background Document together with SEAC's draft opinion (all part of the universal PFAS restriction proposal process in the EU) have conducted weight-of-evidence assessments on PFAS uses and alternatives in batteries.^2,3,5^ The Background Document summarizes the available information on the availability and feasibility of alternatives in a table for broadly defined uses. It concludes that for binders in primary and secondary batteries “feedback from many stakeholders shows that alternatives are not available at present, but that binders for some applications are in development and likely to be feasible in the future.”^2^ Our study identified several alternatives to binders in batteries already on the market, see *e.g.*, Table 5. Furthermore, the Background Document concludes that alternatives in Li-ion battery electrolytes and separator films/coatings are not feasible, either for a specific application (*e.g.* electric vehicles) or require more time for development. SEAC agrees with the assessment provided in the Background Document, but notes uncertainties (negligible or significant, depending on use).^5^ Our study provides additional information that reduces these uncertainties and show that technically feasible alternatives are available.

Cathode chemistry strongly influences battery performance and the technical requirements placed on each component. Therefore, our separation of these into individual uses allowed for a granular assessment, in which certain alternatives provide the required functions in some uses but not others. For example, LFP battery cathodes can be processed with water-based binders, enabling a wide range of alternatives. While the metal oxide chemistries (LCO, NMC, NCA, LMO and LTO) are sensitive to water, some alternative technologies exist on the market for these chemistries as well.

We have shown in our work that certain uses (*e.g.* Li-ion/Liquid electrolyte/Separator and Li-ion/Layered metal oxides/Anode) already on the market adopt PFAS-free alternatives, even though they have been overlooked in previous use-mapping studies or by individual stakeholders.

We also show that substitution with substances other than PFASs would not automatically result in a less-functioning battery as the acceptable level of performance may still be achieved. Instead, PFAS-free alternatives could provide additional benefits. For instance, water-based binders do not require the NMP solvent and therefore eliminate the need for the energy-intensive solvent recovery step.

Our assessment provides the evidence that several (LFP chemistry) or some (LCO, NMC, NCA and LMO chemistries) technically feasible cathode alternatives with the technical function “binder” are already on the market. By categorizing statements such as “PVDF is thermally stable” as properties rather than technical functions, we separate intrinsic material properties from required functions and identify potential alternatives based on required functions, including alternative designs, processes, or technologies. For example, separator coatings may require either binder or adhesion/cohesion promoter function, which is critical information for alternatives assessment. Fig. 5b demonstrates that PFAS-free Li-ion battery cells can be manufactured through material selection (*e.g.*, LFP cathode, graphite anode, non-PFAS electrolyte additives and separators without PFAS coatings such as coatings based on PMMA) for certain applications. PFAS-free Na-ion batteries may be designed for all applications (*e.g.* with a PBA cathode).

### 4.2. Addressing limitations

This work contains several limitations and uncertainties. First, only about one-fourth of the Annex XV survey attachments mentioning batteries were assessed, and flow batteries or other battery storage technologies were excluded. The discrepancy between the flow diagram (Fig. 4, *e.g.*, showing sealing as a significant use) which lists all records in the database and the list of potential uses (with only one sealing use) which only lists potential PFAS uses based on availability of information highlights limitations of the framework when data availability is low. The main uncertainty in our approach arises not from the logical framework itself but from the completeness and quality of the Annex XV consultation comments. In the fast-developing battery field, new technologies (*e.g.*, lithium–sulfur batteries) or processes introduced after September 2023 may have been overlooked and therefore not qualified as potential PFAS uses. In addition, interpretation of stakeholder comments increases uncertainties. The initial evidence base was illustrated to be uneven and may result in two directions: toward overestimation, when vague or repeated claims are taken as indicative of real uses, or toward underestimation, when stakeholders with relevant information did not provide detailed submissions. A second source of uncertainty lies in the supporting evidence, which is extensive but may not be comprehensive. Here, non-response bias might arise when requesting information from battery stakeholders because stakeholders without PFAS-free alternatives may have been less willing to provide information. Market volumes at the level of individual uses are generally not publicly available, which presents an additional limitation. Finally, uncertainty is not uniform across battery types: Li-ion batteries have extensive supporting data, whereas primary batteries and emerging technologies have sparser evidence, which we explicitly acknowledge. For cases with limited data, we can show that targeted case studies are needed also in future work, exemplified with Li-SOCl_2_, for which we could confirm the PFAS use only based on the academic literature.

Despite these limitations, several features of the approach increase confidence in the results. We aim to include any information, regardless of the amount of details. Overall, our approach of combining multiple methods may help address several of the above-mentioned limitations simultaneously. We describe this in detail in the first paragraph of Section 2.2.6. “Integration of lines of evidence.” In addition, individual limitations are addressed through specific methodological choices. First, we do not take stakeholder claims at face value but apply a transparent weight-of-evidence assessment, incorporating teardown-analysis studies, interviews, and the academic literature to confirm or contradict each potential use. This approach allowed us to identify cases where previous studies have overstated PFAS use in batteries and to correct repeated claims that had become “urban legends,” and instead consider them as “non-PFAS uses”. Second, the requirement for substance-level information reduces misclassification risk, particularly where stakeholders listed undefined PFASs or misidentified substances such as LiPF_6_. Third, by assessing the technical function and not the inherent property of PFASs, *e.g.*, “binder” instead of “thermal stability”, we go beyond only listing current substances in use. Finally, the use of market availability as a proxy for technical feasibility provides a conservative and observable benchmark that does not depend on internal R&D capacity. Together, these considerations suggest that while some uncertainties remain, primarily due to incomplete Annex XV public consultation data, the overall conclusions are effective where evidence is strongest (*e.g.*, Li-ion batteries) and appropriately cautious where evidence is limited.

### 4.3. Future research directions

Overall, our database and supporting evidence provide large amounts of information in a structured and transparent manner. As each record is linked to a consultation comment, interested parties can identify reported PFASs in batteries associated with specific processes and configurations to inform their own assessments. Records also include information on battery applications, end-use function, service function, and technical requirements. Although not further assessed in this work, we set the basis for systematic alternatives assessment and investigation under the essential-use concept, where technical, end-use, and service functions are evaluated in relation to the application.

Publicly available detailed and confirmed information on uses, substances and functions is valuable across many contexts. The results of this work can support environmental monitoring through improved suspect lists, help downstream users identify where substances of concern may appear in supply chains, and inform battery recycling, where knowledge of composition is increasingly important. It can also support regulatory processes related to classification, restriction, and extended producer responsibility, among others.

Beyond the immediate uses described above, we encourage further research and regulatory action on PFAS uses in batteries to adopt our framework. The present version focuses on potential and confirmed PFAS uses, but the structure allows expansion to include quantitative performance data, information on production volumes, and links to patents or supply chain actors. Integrating these elements would enable more detailed alternatives assessments and support priority setting for substitution and innovation.

Our framework can also be used when assessing other substances of concern in batteries. When requesting additional information, the Battery Use & Function Framework (outlined in Tables 1 and 2 and procedure described in Fig. 2) could be used to set minimum data requirements for stakeholders, reducing uncertainty by avoiding interpretive steps. This could be implemented by substitution and innovation centers, regulators, industry, academia, and others.

Finally, the methodology can be extrapolated to assess PFAS uses, functions, and substitution potential in other (complex) PFAS uses, including green energy technologies such as solar cells, electrolyzers, and heat pumps. This work demonstrates the need to incorporate incomplete data into a suitable framework which clearly defines potential PFAS uses and functions, particularly for complex cases, from which multiple lines of evidence may confirm or contradict these uses. Future research could increase efficiency by applying AI-based search and text-mining tools in selected methodological steps, such as source collection or extraction of information into a defined framework. Such tools could systematically screen technical submissions, high-quality and relevant scientific literature, reliable internet sources, and commercially relevant patent databases for PFAS-related uses and functions and incorporate quantitative performance data to support more detailed alternatives assessments with suitable human-in-the-loop approaches for accuracy and quality control.

## Author contributions

A. R.: conceptualization, methodology, investigation, data curation, formal analysis, visualization, writing – original draft, writing – review & editing. A. J. M.: methodology, data curation, visualization, writing – review & editing. J. G.: methodology, writing – review & editing. M. S.: methodology, writing – review & editing. Z. W.: methodology, writing – review & editing. R. L.: writing – review & editing. C. A. N.: writing – review & editing. X. T.: writing – review & editing. I. T. C.: conceptualization, methodology, writing – review & editing, funding acquisition, supervision.

## Conflicts of interest

There are no conflicts to declare.

## Supplementary Material

EM-OLF-D6EM00349D-s001

EM-OLF-D6EM00349D-s002

EM-OLF-D6EM00349D-s003

## Data Availability

All available data presented in the study are available in the supplementary information (SI). Supplementary information: SI1 (.doc) contains additional details on the methods and results including details on the previous use-mapping studies, input from the battery stakeholders, teardown-analysis studies, and literature review. SI2 (.xlsx) tabulates those results and contains the Battery Use & Function Framework database of records of PFAS uses along with references. SI3 (.html) is a downloadable file that can be opened in a web browser for the interactive version of the flow diagram (Fig. 4) showing the quantities of each flow. All references cited in the SI are listed in the article's reference list, except for excluded studies and input from battery stakeholders unless specifically cited in the article. The use of large language models was in accordance with the method described in SI, Section 2.2.2. In addition, Microsoft Copilot was used for limited text editing, maximum one paragraph at a time but not more than 2.5% of the full paper. This model was also used to brainstorm the title and table of content graphic. However, large language models were not used for screening Annex XV consultation comments and supporting evidence or extracting information from these. They were also not used in the development of the framework or production of the database. See DOI: https://doi.org/10.1039/d6em00349d.
