# Supplementary material for: Inside the battery box: a cross-technology assessment of PFAS uses, functions, and substitution potential
Source: Environ Sci Process Impacts. 2026 Nov 12. Online ahead of print. doi: 10.1039/d6em00349d (PMC13755903; doi:10.1039/d6em00349d)
Supplement: Supplementary file 1 [file EM-OLF-D6EM00349D-s001.pdf]

Supplementary Information (SI) for Environmental Science: Processes & Impacts.
This journal is © The Royal Society of Chemistry 2026

Electronic Supplemental Information to

Inside the Battery Box: A Cross-Technology Assessment of PFAS Uses, Functions, and Substitution Potential

Amanda Rensmo[1]†, Anna J. Miller[2], Juliane Glüge[2]‡, Martin Scheringer[2], Zhanyun Wang[3,4,5], Rainer Lohmann[6], Carla A. Ng[7], Xenia Trier[8], and Ian T. Cousins[1]†

[1] Department of Environmental Science, Stockholm University, SE-10691 Stockholm, Sweden
[2] Institute for Biogeochemistry and Pollutant Dynamics, ETH Zurich, 8092 Zurich, Switzerland
[3] University of Southern Denmark, Department of Green Technology & Danish Institute for Advanced Study (DIAS), 5230 Odense, Denmark
[4] Empa -- Swiss Federal Laboratories for Materials Science and Technology, Technology and Society Laboratory, 9014 St. Gallen, Switzerland
[5] NCCR Catalysis, ETH Zürich, 8093 Zürich, Switzerland
[6] Graduate School of Oceanography, University of Rhode Island, Narragansett, RI 02882, USA
[7] Department of Civil & Environmental Engineering and Environmental and Occupational Health, University of Pittsburgh, Pittsburgh 15261, Pennsylvania, United States
[8] Department of Plant and Environmental Sciences, Section for Environmental Chemistry and Physics, University of Copenhagen, Thorvaldsensvej 40, DK-1871 Frederiksberg C, Denmark

† Corresponding authors: A.R. amanda.rensmo@aces.su.se, I.T.C. ian.cousins@aces.su.se
‡ Present address: Section Industrial Chemicals, Swiss Federal Office for the Environment, Bern 3011, Switzerland

Table of Contents

1. LISTS OF ACRONYMS, ABBREVIATIONS AND CHEMICALS 3

1.1. Acronyms and abbreviations 3

1.2. Chemicals 3

2. METHOD 6

2.1. Qualifying a Potential PFAS Use in Batteries 6

2.2. Integrating Supporting Evidence 10

3. SUPPORTING EVIDENCE 19

3.1. Previous use-mapping studies 19

3.2. Input from battery stakeholders 35

3.3. Detection of PFAS by teardown-analysis studies 46

3.4. Selected case studies: Survey of the academic literature 53

3.5. Potential alternatives as stated in Annex XV consultation comments 60

4. INTEGRATION OF LINES OF EVIDENCE 63

4.1. Li-MnO2 battery 63

4.2. Li-SOCl2 battery 63

4.3. Li-SO2 battery 64

4.4. AgO2 battery 64

4.5. Li-ion battery 64

4.6. Na-ion battery 70

4.7. Lithium metal and solid-state batteries 72

4.8. Lead acid battery 74

REFERENCES 75

1. LISTS OF ACRONYMS, ABBREVIATIONS AND CHEMICALS

1.1. Acronyms and abbreviations

AgO2	Silver oxide, battery chemistry
BAJ	Battery Association of Japan
BMS	Battery management system
CEI	cathode electrolyte interphase
Danish EPA	Danish Environmental Protection Agency, Miljøstyrelsen
DTA	Differential thermal analysis, material characterization method
ECHA	European Chemicals Agency
EDS	Energy-dispersive X-ray spectroscopy, material characterization method
EDX	see EDS
EV	Electric vehicle
EU	European union
FTIR	Fourier transform infrared spectroscopy, material characterization method
ICP-OES	Inductively coupled plasma optical emission spectroscopy, material characterization method
Layered metal oxide	Several battery chemistries such as LMO, LCO, NMC, NCA, but not LFP
LCO	Lithium cobalt oxide, battery chemistry
LFP	Lithium iron phosphate, battery chemistry
Li-ion	Lithium-ion, battery technology
Li-MnO2	Lithium manganese oxide, battery technology
Li-SO2	Lithium sulfur dioxide, battery technology
Li-SOCl2	Lithium thionyl chloride, battery technology
LMO	Lithium manganese oxide, battery chemistry
LNMO	Lithium nickel manganese oxide, battery chemistry
LTO	Lithium titanate oxide, battery chemistry
Na-ion	Sodium-ion, battery technology
NCA	Lithium nickel manganese aluminum oxide, battery chemistry
NiCd	Nickel cadmium, battery technology
NiMH	Nickel metal hydride, battery technology
NMC	Lithium nickel manganese cobalt, battery chemistry
NGO	Non-governmental organization
NTO	Niobium Titanate Oxide, anode composition
OECD	Organisation for Economic Co-operation and Development
OEM	Original Equipment Manufacturer
PBA	Prussian Blue Analogues, battery chemistry
RAC	Risk Assessment Committee
REACH	Registration, Evaluation, Authorisation and Restriction of Chemicals, EU:s chemical legislation
SEAC	Socioeconomic Assessment Committee
SEI	Solid electrolyte interphase
SEM	Scanning Electron Microscopy, material characterization method
TRL	Technology readiness level
TGA	Thermogravimetric analysis, material characterization method
US DOE	The United States Department of Energy
XPS	X-ray photoelectron spectroscopy, material characterization method
XRD	X-ray diffraction, material characterization method

1.2. Chemicals

Al2O3	Aluminum dioxide

AlOOH	Aluminum oxyhydroxide
AMAC	[listed in Background document but not further defined]
AMMA	[listed in Background document but not further defined]
BETI	bis(perfluoroethanesulfonyl)-imide
BOB	bis(oxalato)borate
CMC	Carboxymethyl cellulose
ETFE	Ethylene tetrafluoroethylene
FEP	Fluorinated ethylene propylene
FKM	*Fluorkautschukmaterial*, FKMs; copolymers with vinylidene fluoride (VDF)
FP	Fluoropolymer
HF	Hydrogen fluoride
HFIP	Hexafluoroisopropanol
LiBETI	see BETI
LiBOB	see BOB
LiCMC	see CMC
LiFAP	Lithium tri(pentafluoroethyl)triphosphate
LiFSI	Lithium bis(fluorosulfonyl) imide
LiFTFSI	Lithium fluorosulfonyl-trifluorosulfonyl imide
LiPF6	Lithium hexafluorophosphate
LiTFSI	see TFSI
NaBOB	see BOB
NaTFSI	see TFSI
NMP	n-methyl-2-pyrrolidone
PAA	Polyacrylic acid
PAN	Polyacrylonitrile
PBT	[listed by RECHARGE (#8680) but not further defined]
PE	Polyethylene
PEEK	Polyether ether ketone
PEI	Polyethylenimine
PEO	Polyethylene oxide
PFA	Perfluoroalkoxy
PFASs	Per- and polyfluoroalkyl substances
PFCAs	Perfluoroalkyl carboxylic acids
PFPE	Perfluoropolyether
PI	Polyimide
PMA	[listed in Background document but not further defined]
PMMA	Poly(methyl methacrylate)
PP	Polypropylene
PTFE	Poly(tetrafluoroethylene)
PVC	Poly(vinyl chloride)
PVDF	Poly(vinylidene fluoride)
PVDF-HFP	Poly(vinylidene fluoride-co-hexafluoropropylene)
PVDF-TrFE	Poly(vinylidene fluoride-co-trifluoroethylene)
PVF	Poly(vinyl fluoride)
PVP	Poly(vinylpyrrolidone)
R1234yf	2,3,3,3-Tetrafluoropropene
S	Sulfur
SA	Sodium alginate
SBR	Styrene butadiene rubber
SCFP	Side-chain fluoropolymer
SiO2	Silicon oxide
SO2	Sulfur dioxide

Teflon	Trademark name for PTFE
TFSI	bis(trifluoromethanesulfonyl)imide
THV	TFE-HFP-VF2, as defined in OECD 2025
TiO2	Titanium dioxide
ZrO2	Zirconium dioxide

2. METHOD

2.1. Qualifying a Potential PFAS Use in Batteries

2.1.1. Collection of information

The Annex XV public consultation of the universal PFAS restriction proposal, through the REACH regulation in EU, resulted in more than 5 600 survey comments (although this also includes duplicates, such as several submissions from the same company located in several (European) countries, multiply cited documents or peer-reviewed articles, or simply duplicate submissions from one stakeholder).

2.1.1.1. Records identified

We downloaded all survey comments from European Chemicals Agency's (ECHA's) Registry of restriction intentions until outcome[1] and all attachments from a Google Drive set up by Thomas Goorden. All reports were imported and indexed in document-handling software DocFetcher.[2] A total of 3 269 individual files were imported as attachments (including duplicates) (only ~0.1% were not imported due to file errors but were checked individually to not contain any relevant information), while all survey comments were successfully imported.

2.1.1.2. Records screened

As stated in the main text, the material was searched for "batter*" (to include both 'battery' and 'batteries'), The addition of the term "function" for the attachments was justified by the large number of hits for "batter*" alone and the project's aim of identifying the specific functions of PFASs in batteries.

To validate this adjustment, the results from two search strings were compared. The DocFetcher software includes a sorting function "Score (%)",a calculation of the percentage of searched words per total words.[3] For the "batter*" search, the first 100 reports were compared to all reports identified in the "batter* AND function," for which 74 were included in both searched. Thus, the overlap was considered sufficient.

2.1.1.3. Reports sought for retrieval

In DocFetcher, we could search all survey comments and/or attachments simultaneously. The program allowed both reading the text directly, so that fast survey was possible, and the possibility to open the parent folder to survey the text in original style (pdf, Excel or word files). As such, a screening was performed directly in the program and in this retrieval, records were only considered further if they mentioned battery more than twice but only if function was related to battery. Information was excluded if it was concluded to not add new information, e.g. attachments of full studies not processed by submitter, such as the Glüge et al (2020).[4]

2.1.1.4. Reports assessed for eligibility

When assessing the retrieved reports, eligibility criteria was as follows:

- Were targeted at this call, e.g. not an attachment of a whole book on batteries
- Contained new information, i.e., not when restating the information provided in the Annex XV report
- Referred specifically to an individual (or several individual) use(s) of PFASs in batteries, e.g. not only to battery in general, such as "Fluoropolymers and Fluoroelastomers in products for material handling / intralogistics industry (e.g., non-road mobile machinery, large-scale fixed installations), including batteries, accessories, spare parts for the repair, and manufacturing equipment." (#7997) hindering further categorization)
- Only if "battery" was referring to battery technology, e.g., not "a comprehensive battery of biocompatibility tests" (#6208)

The report was only taken forward if all four criteria were fulfilled.

2.1.2. Development of the Battery Use & Function Framework

Previous use-mapping studies have categorized batteries in various hierarchies. In Figuière et al. (2025), the uses were defined from a top-down approach, with division into uses and sub-uses based on expert judgement for each sector. As such, there is little consistency between the categorizations between the sectors. For example, (Li-ion) batteries are found within the sub-use category in electronics but are as its own use category in the Energy sector. In Glüge et al. (2020), an overview of different batteries is provided in the Industry branch "Energy sector", which is reported similarly in the Annex XV report and Background document. The latter includes a third use level (application) in which general uses of batteries are categorized into binders, electrolytes and separator coatings. Furthermore, the electrodes often include not only the electroactive material but may also consist of a conductive additive and binder material. The liquid electrolyte is a formulation of salts and solvents while the solid-state battery technology refers to (semi)-solid electrolyte.[5]

In the guidance describing the information requirements under REACH, uses[a] of batteries are reported as "Electrolytes for batteries" (PC42) and "Electrical batteries and accumulators" (AC3). Overall, the function of each substance in a specific battery use is not systematically reported.

However, batteries make up a wide variety of technologies, where the cathode (also referred to as the battery chemistry) is central. The first rechargeable battery (lead acid) was invented in 1859. A century later, batteries based on lithium came to the market. In 1980, the lithium cobalt oxide ($LiCoO_2$, LCO) cathode and the graphite anode were discovered, commercialized by Sony in 1991.[6] The history of battery development illustrates how different battery chemistries have emerged and coexisted over time, including lithium manganese oxide ($LiMnO_2$, LMO), lithium nickel manganese cobalt oxide ($LiNi_{1-x-y}Mn_xCo_yO_2$, NMC), lithium nickel cobalt aluminum ($LiNi_{1-x-y}Co_xAl_yO_2$, NCA) and lithium iron phosphate ($LiFePO_4$, LFP). Lead acid batteries, first commercialized in the 19th century, remain widely used today e.g., in back-up power systems, while lithium-ion batteries now dominate the market in a wide variety of applications. Continued growth in energy-storage applications has also driven the development of additional battery technologies, further expanding the diversity of chemistries and designs on the market.[5,7]

In this work, the aim is to provide information that could inform future work of direct comparisons between current uses of PFAS and future alternative materials in the same use. As such, we strive to link uses to functions via the substance. Compared to simple articles[b] like an O-ring or a processing aid, the battery can be considered a complex article.[1] Therefore, three main use levels are needed to reflect the complexity of a specific battery technology in which the Battery Use Level 2 is further expanded with three sub-use levels to distinguish different battery technologies from each other. This is described in the main text as well as in Table 1. In the conceptualized bottom-up approach adopted in this work, we have denoted the

---

[a] REACH, Article 3 (24) defines a use as "any processing, formulation, consumption, storage, keeping, treatment, filling into containers, transfer from one container to another, mixing, production of an article or any other utilisation" and use descriptors are explained further in the ECHA's "Guidance on Information Requirements and Chemical Safety Assessment Chapter R.12: Use description", including e.g. sector of use (SU) related to market, product category (PC) related to consumers and article category (AC) related to service life.

[b] REACH, Article 3(3) defines an article as "an object which during production is given a special shape, surface or design which determines its function to a greater degree than does its chemical composition" which distinguish it from a substance or a mixture.

level closely related to sector or end-use application as “Battery Use Level 3: Application”. This contrasts with previous use-mapping studies in which “application” may be considered the level with most simple article.

We adopt the Functional Substitution Framework categories to link the substance to the defined use.[1] We further conceptualize this approach by categorizing the first level using the functional use categories according to the functional use categories defined in *Internationally Harmonized Functional, Product and Article Use Categories* defined by the OECD (2025).[1] In their work, 124 functional use categories were defined “based on the function that the chemical substance serves when used”, following a consolidation from 200 unique functional use categories from US EPA, Consumer Specialty Product Association and ECHA, among others. Examples of technical functions are Binder, Catalyst, Dye, Hardener, Lubricating agent, and an “Other” category.

2.1.3. Recording of information into the database

The Annex XV consultation comments were read in their entirety and surveyed for a link between PFAS and a use in battery technology. If possible, information was incorporated *verbatim* (i.e. as written directly in the Annex XV survey comments or attachments), apart from a few exceptions. To simplify assessment, conformity was obtained by using the same naming convention, see examples provided in the main text. In addition, a “rechargeable battery” was recorded as “secondary” in the Battery Use Level 2b: Battery type.

Additional information that did not relate directly to each use level but still could provide information about the battery as a whole was included in parenthesis. This includes e.g., battery configuration or format, manufacturing process (related to Battery Use Level 2: Battery Technology) or anode or electrolyte composition (related to Battery Use Level 2.2: Battery chemistry). For Technical Function, information was adjusted slightly translating stakeholder information to fit the 124 functional use categories. If necessary for the translation, the *verbatim* text is added to the entry within quotation marks in a parenthesis after categorization, e.g. ‘Cell* (“Pack”)’ if other evidence indicated strongly that it should be altered (e.g., here the case where the stakeholder refers to a cathode, which is part of the cell and not the pack). As described in the main text, the “*” denotes an entry which is not directly mentioned by the stakeholder but could be inferred from other entries, such as the cathode is always part of the cell.

Figure S1. PRISMA flowchart of identifying Annex XV consultation comments.

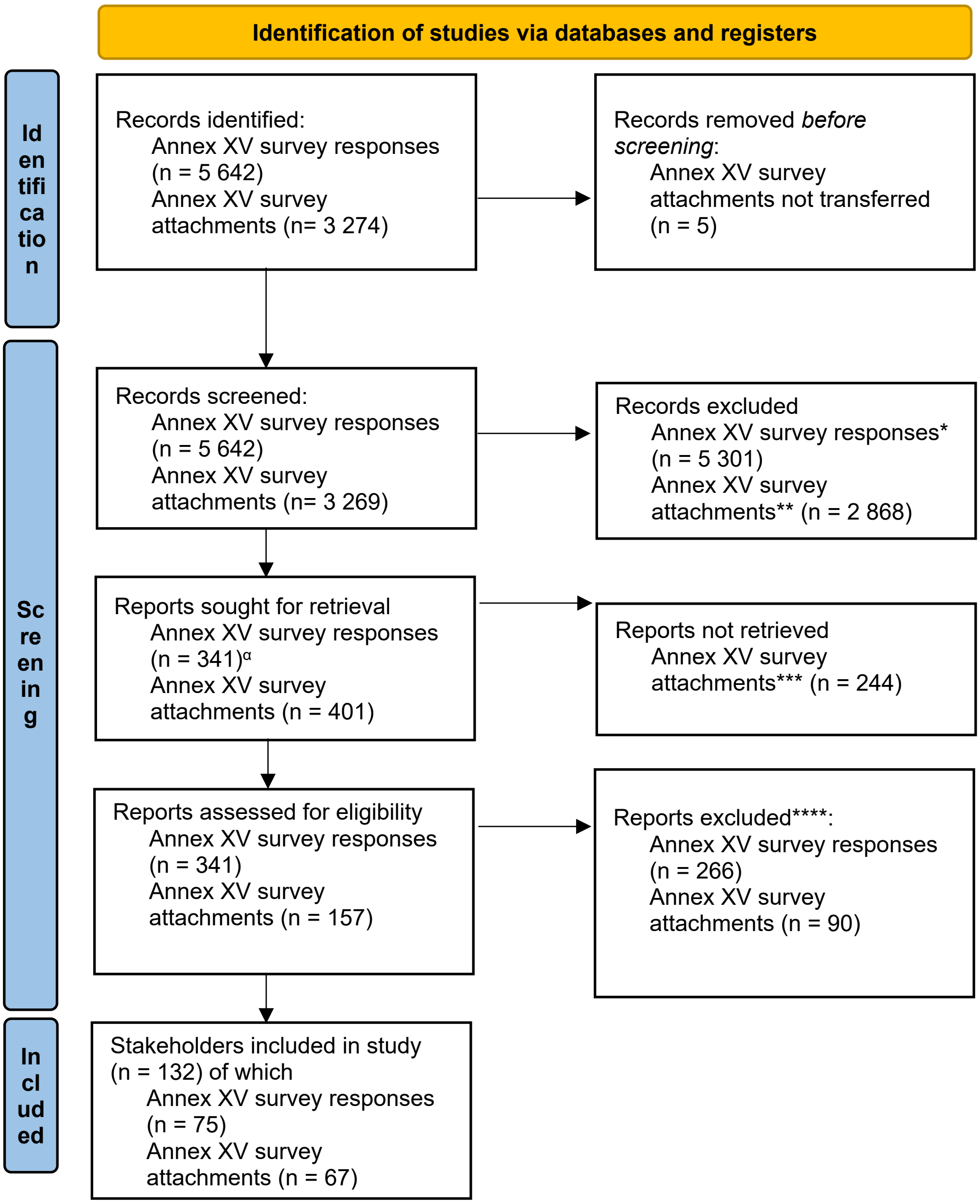

Adapted from: Page MJ, et al. BMJ 2021;372:n71. doi: 10.1136/bmj.n71.[8]

* Not including "batter*"

** Not including "batter* AND function"

*** See: Reports sought for retrieval above

**** See: Reports assessed for eligibility above

[α] Note that 341 is also the number of records in database by coincidence; no relationship exists between the two.

2.2. Integrating Supporting Evidence

2.2.1. Previous use-mapping studies

Based on expert judgment, a collection of previous use-mapping studies was compiled. To be eligible in our work, a use-mapping study is a report, peer-reviewed article or book investigating the uses of PFAS. These include e.g., Glüge et al. (2020), Gaines (2023), the Annex XV report, the Background Document and Figuière et al. (2025), which investigate the PFAS use and alternatives across sectors.[4,9–12] We identified several referenced studies which were also relevant to our aim, such as Kissa (2001), Ebnesajjad (2013) and Ameduri (2018).[13–15] We also identified several reports which were related specifically to batteries, e.g. Rensmo et al. (2023) and Savvidou et al. (2024),[16,17] or to sectors related to batteries, such as green energy or electronics.[18,19] Reports covering PFASs or PFAS sub-groups were identified, including reports on fluoropolymers.[20,21]

However, we do not consider academic literature in the battery field as previous use-mapping studies, as it rather describes functions and new substances. They would also result in a large set of peer-reviewed articles with unclear application on the market. In addition, we did not include monitoring studies, as they cannot link the detected PFASs to a PFAS specific.

2.2.2. Input from battery stakeholders

A list of actors along the battery value chain, i.e., battery stakeholders, was assembled using large-language model platforms. Microsoft Co-Pilot and ChatGPT were prompted to map the value chain of batteries, with a focus on the European market. These were also specifically requested for additional information about companies providing water-based binders, relevant for the assessment of PFAS-free binders in cathodes. Then expert knowledge of the authors was adopted to expand the list. The list was curated afterwards to streamline the categorization across sectors, into the categories of Battery Manufacturer, Battery Materials and Components, Battery Recycling, Battery Systems, Integration & OEM, Downstream User, Equipment, Testing, & Support, Raw Materials, Research Institute or Consultancy, and Trade bodies, Alliances & Investor networks. Details acquired from the large-language models were noted in a separate column, including e.g. specifications regarding product category. The full list can be found in SI2, 2.2.

Considering the length of the list, and the time-extensive task of contacting organizations without previous contact, a selection (38 %, percent of total) of organizations was performed including battery materials and components (68%, percent within category), battery manufacturers (68%), battery systems, integration & OEM (12%), downstream users (46%), research institute or consultancy (33%), and trade bodies, alliances & investor networks (17%). No organization representing raw materials, equipment, testing and support or battery recycling was contacted and not all organizations stemming from expert judgement were contacted.

Contact was enabled via e-mail (available through expert knowledge, via large-language models or on the organization's website) or, if no e-mail was available, through a contact form on the website.

The first contact was initiated with a short description of the research project and concluded with some information on the rationale of participation and response considerations. Following a description of initial results and specific requests in this inquiry, the e-mail or website request read as follows:

“

/…/ In short, PFAS are any compound that contain a -CF2- or -CF3 moiety, for example fluoropolymers (such as PVDF and PTFE) and smaller molecules such as TFSI/HNTf2/bis-FMeSI and other bis-FASI homologues, but not e.g. PF6. My sources include reports and statements in various technical contexts, mainly submitted in the open consultation for the universal PFAS restriction proposal – the process in which EU is assessing a potential full ban on PFAS. Here, I have noticed that stakeholders cite the use of PFAS in batteries including PTFE, PVDF and other fluoropolymers in the cathode binder and possibly separator coatings and anode binder. In addition, the use of PFAS is mentioned for the liquid electrolyte with TFSI and other various non-polymeric substances, either as additives or main component, as well as uses of several PFAS types in solid state electrolytes. Some general uses included in the comments are specific fluoropolymers for gaskets, seals, vents etc.

Furthermore, RECHARGE, Europe’s industry association for advanced rechargeable and lithium batteries, submitted several comments on PFAS uses in batteries, stating among other things: “The PFAS (Per and polyfluoroalkyl substances) REACH restriction proposal will have a major impact on the battery industry. /…/ For specific applications where PFAS are used in batteries, RECHARGE is requesting derogations and additional transition times to provide sufficient time for the battery industry to identify and implement alternative non-PFAS solutions.” (Executive summary, first submission with reference number 3925)

Here I would like to dive a bit deeper and learn about the situation of individual actors along the value chain, specifically [insert location in the value chain]. I am interested in the status of the development of alternatives, that is: What is already market-ready? Or is more time needed to develop alternatives? As a [location in the value chain], I am now interested to hear your (company’s) perspective. Some of my questions are as follows, but if we open up a dialogue, we can go into more details as well.

1. What technical requirements are set for the batteries used in your products? Specific power or energy properties, safety aspects, long cycle life, fast charging, etc.? Are any of these linked to the use of PFAS?
2. Are you aware of any fluoropolymers such as PVDF or PTFE, or any other PFAS, that are used in your products and/or your suppliers’ products?
3. Are you aware of other uses of PFAS in your products and/or the manufacturing of your products that I have not mentioned?

I am reaching out to actors along the global battery value chain to gain a better understanding of the current market situation. This includes battery material providers, cell manufacturers, battery component developers, (automotive) OEMs, consumer electronic downstream users, energy solutions actors, among others. *My aim is to obtain a overview of the uses of PFAS on the real market, rather than a perspective on laboratory scale developments. /…/*

”

In the reminder sent in January, the questions were altered slightly.

"

1. /…/ What options are already market-ready?
2. In which cases is more time needed to develop alternatives? How long do you expect for this development to take?
3. What do you think of a derogation for battery components for 13,5 years in the universal PFAS restriction proposal? And what if they would give a time-unlimited derogation?/…/

"

For certain requests, such as organizations with limited space in their website form or if a personal contact had already been established, the message was adopted slightly, but still contained the main concept. The three questions in seen in the request above were used as questions for the semi-structured interviews.

2.2.3. Detection of PFAS in teardown-analysis studies

The search string for each website as well as the date of search are described in Table S1. The exclusion of reports is described in Figure S2. To evaluate eligible reports, the full set of 29 studies were read in their entirety, and only reports that mentioned the material in the battery cell were included.

Table S1. Search strings for database search of teardown-analysis studies.

Date	Database	Search string
2025-12-03	WoS	teardown OR disassembly (Topic) and batter* (All Fields) and pouch OR cylindrical OR coin OR prismatic (All Fields)
2026-02-25	Scopus	(TITLE-ABS-KEY (teardown OR disassembly) AND TITLE-ABS-KEY (pouch OR cylindrical OR coin OR prismatic) AND TITLE-ABS-KEY (batter*))
2026-02-25	SciFinder	Abstract/Keywords: (teardown OR disassembly) AND (batter*) AND pouch OR coin OR cylindrical OR prismatic)

Figure S2. PRISMA flowchart of identifying teardown-analysis studies.

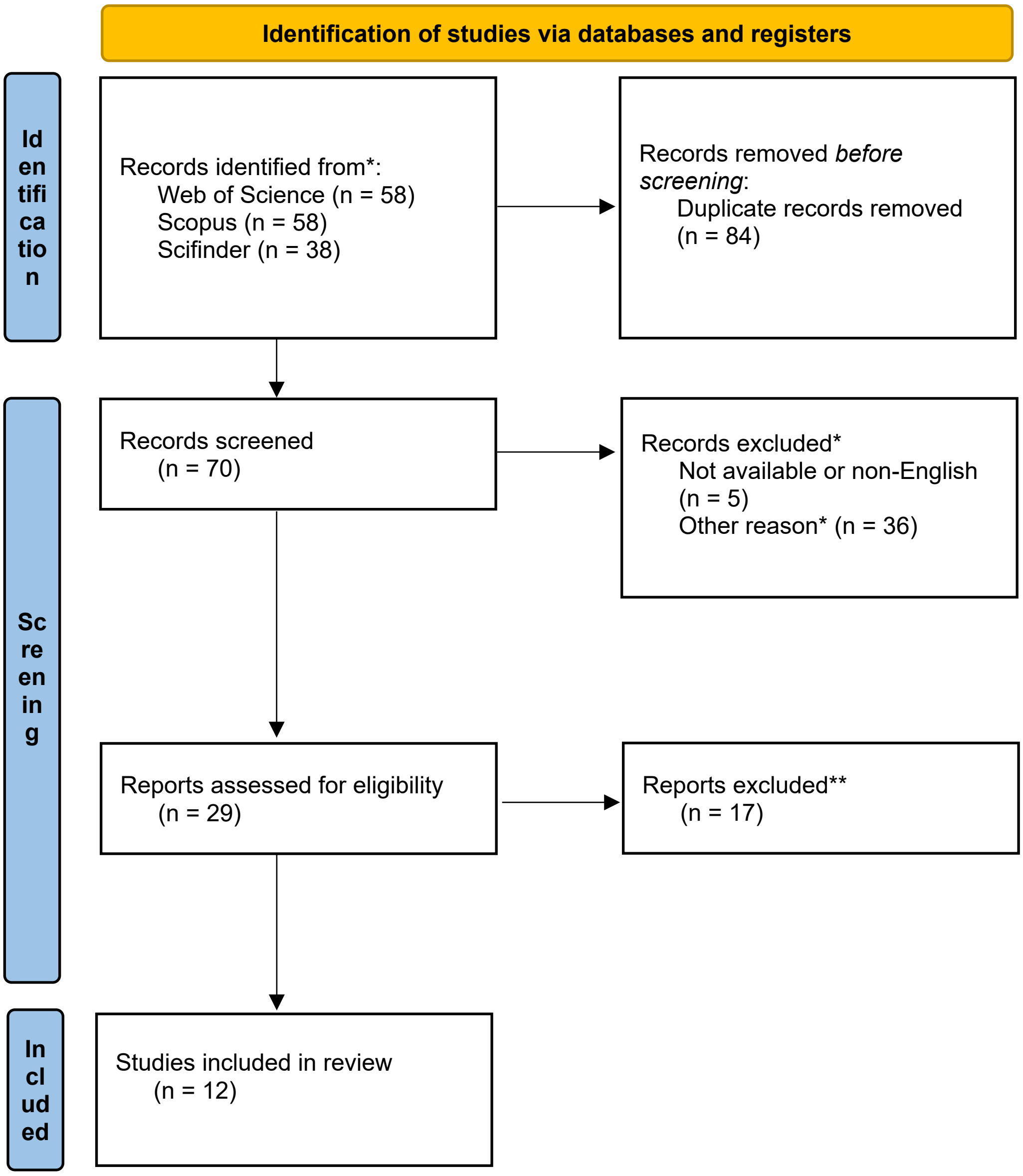

Adapted from: Page MJ, et al. BMJ 2021;372:n71. doi: 10.1136/bmj.n71. [8]

* Title or abstract does not describe material analysis or commercial batteries.

** No information available about material in battery cell, either PFAS or non-PFAS.

2.2.4. Selected case studies: Survey of the academic literature

To complement the other source types, we selected three case studies to be surveyed in the academic literature. The aim of the selection was to cover a broad range of battery technologies. Thus, a pre-screening was conducted to identify suitable scope for three cases as described below. We searched in three or four databases per case study with only slightly adjusted search strings to allow for discrepancies in the database organization (Table S2). Any information relevant to PFAS in the selected case studies was collected, particularly if it included reference to commercial use or stated functions provided by PFAS or technical requirements. In addition, information related to other battery technologies, chemistry or component was briefly noted, see sub-sections titled “Notes on /…/” in section 4.

Table S2. Search strings for database search of selected case studies.

Case	Date	Database	Search string
1	2025-10-29	WoS	"lithium-ion battery" OR "lithium ion battery" OR "lithium-ion batteries" OR "lithium ion batteries" OR LIB OR LIBs (Topic) and "iron phosphate" OR LFP OR LiFePO4 OR FePO4 (Topic) and binder OR binders (Topic) and cathode (Topic) and Review Article (Document Types)
	2025-10-29	Scopus	(TITLE-ABS-KEY ("lithium-ion battery" OR "lithium ion battery" OR "lithium-ion batteries" OR "lithium ion batteries" OR LIB OR LIBs) AND TITLE-ABS-KEY ("iron phosphate" OR LFP OR LiFePO4 OR FePO4) AND TITLE-ABS-KEY (binder OR binders) AND TITLE-ABS-KEY (cathode)) AND (LIMIT-TO (DOCTYPE , "re"))
	2025-10-29	Scifinder	Abstract/Keywords: ("iron phosphate" OR LFP OR LiFePO4 OR FePO4) AND (binder OR binders) AND (cathode) AND (batteries OR battery); Document type: Review
2	2025-12-08	WoS	TS=(separator* NEAR/25 coat*) AND TI=separator* AND TS=batter* and Review Article (Document Types)
	2025-12-08	Scopus	(TITLE-ABS-KEY (separator* W/25 coat*) AND TITLE (separator*) AND TITLE-ABS-KEY (batter*)) AND (LIMIT-TO (DOCTYPE , "re"))
	2026-02-23	Scifinder	Abstract/Keywords: ("separator") AND (batter*) AND ("coating*"); Document type: Review
3	2025-10-21	Web of Science	"Lithium thionyl chloride" OR "Li-SOCl2" OR (Li AND SOCl2) (Topic) and battery OR batteries (All Fields) and Review Article (Document Types)
	2025-10-21	Scopus	(TITLE-ABS-KEY (("Lithium thionyl chloride" OR "Li-SOCl2" OR (Li AND SOCl2)) AND TITLE-ABS-KEY (battery OR batteries)) AND (LIMIT-TO (DOCTYPE , "re"))
	2026-02-20	SciFinder	Abstract/Keywords: ("Lithium thionyl chloride" OR "SOCl2") AND (batter*) AND (primary OR (non AND rechargeable); Document type: Review
	2025-10-21	Google Scholar	(Lithium thionyl chloride" OR "Li-SOCl2) AND (batteries OR battery); Review Articles

2.2.4.1. *Case 1: Li-ion/LFP chemistry/Cathode (potential PFAS use no. 6; technical function: "binder")*

In the first case study, we sought to obtain information about the cathode as PVDF with "binder" reported as technical function was the most cited PFAS in batteries in Annex XV consultation comments. However, in the pre-screening, this resulted in a large amount of reports (>3 000 results in Web of Science and >1 800 in Scopus), and similar results were seen also when restricting the search to Li-ion batteries. Thus, we narrowed our search to only one battery chemistry, namely LFP. The identification, screening and inclusion of studies is outlined in Figure S3 and a list of identified records is found in SI2, 2.4.1

Figure S3. PRISMA flowchart of identifying Case 1 (Li-ion/LFP chemistry/Cathode (function: binder)) studies in survey of academic literature.

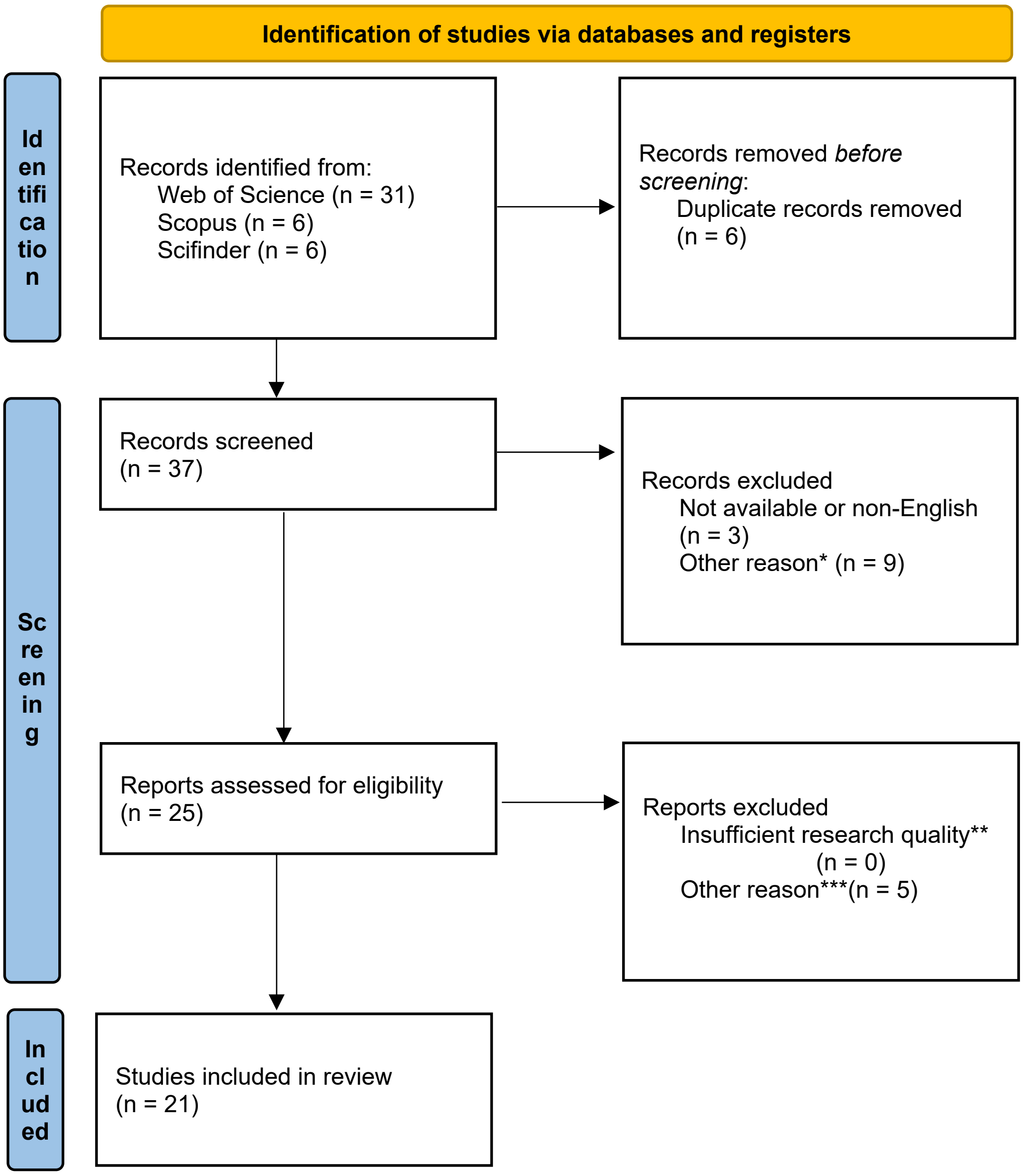

Adapted from: Page MJ, et al. BMJ 2021;372:n71. doi: 10.1136/bmj.n71. [8]

* Reports that focused on recycling processes or other battery technologies (e.g., Lithium-Selenium) were excluded in the screening of titles and abstracts.

** Reports which were deemed not trustworthy, either based on suspicion of large reliance on AI or with concerns of plagiarism or insufficient referencing were excluded in the screening of full texts.

*** Reports without information about material (PFAS or non-PFAS) were excluded in the screening of full texts.

2.2.4.2. *Case 2: Li-ion/Liquid electrolyte/Separator, coating (potential PFAS use no. 17)*

Another common use cited by the Annex XV stakeholders was that of separators or separator coatings, where the latter qualified as potential PFAS use no. 17 in Li-ion batteries (Table 4). In the pre-screening, we found only a limited number of literature reviews describing this use. Thus, instead of formulating a search string only identifying records of Li-ion batteries in title or abstract, we set a widely-defined search string. In the next step, we then excluded studies that exclusively focused on other battery technologies, such as Li-S, aqueous zinc-ion, lithium metal or solid-state batteries. The identification, screening and inclusion of studies is outlined in Figure S4 and a list of identified records is found in SI2, 2.4.2.

Figure S4. PRISMA flowchart of identifying Case 2 (Li-ion/Liquid electrolyte/Separator, coating (potential PFAS use no. 17) studies in survey of academic literature.

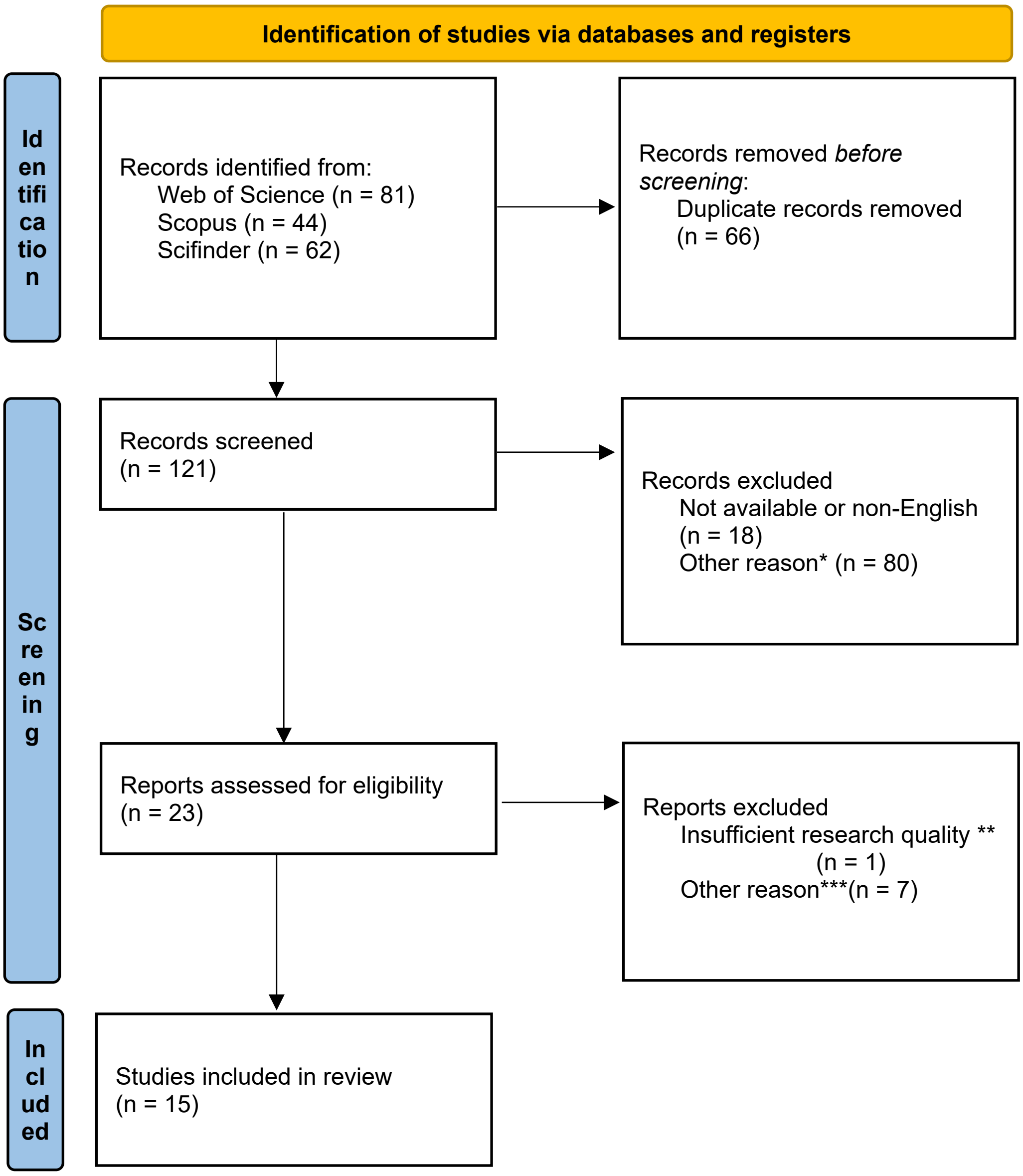

Adapted from: Page MJ, et al. BMJ 2021;372:n71. doi: 10.1136/bmj.n71.[8]

* Reports that focused on novel materials (e.g., metal organic frameworks or functional materials) or other battery technologies (e.g., Li-S or Mg-S) were excluded in the screening of titles and abstracts.

** Reports which were deemed not trustworthy, either based on suspicion of large reliance on AI or

with concerns of plagiarism or insufficient referencing were excluded in the screening of full texts.

*** Reports without information about material (PFAS or non-PFAS) were excluded in the screening of full texts.

2.2.4.3. *Case 3: Li-SOCl2/Li-SOCl2 chemistry/Cathode (potential PFAS use no. 3)*

The third case selected represented one example of a primary battery. Here, we selected the cathode of the lithium thionyl chloride battery was taken forward, which was listed as potential PFAS use no. 3 (Table 4). In the pre-screening, few records were identified. In this case, additional records were obtained by searching a fourth database, Google Scholar to reach a critical mass. Thus, in the screening, we not only included peer-reviewed articles, but also non-peer-reviewed reports as these were noted to contain relevant information about PFAS use. The identification, screening and inclusion of studies is outlined in Figure S5 and a list of identified records is found in SI2, 2.4.3.

Figure S5. PRISMA flowchart of identifying Case 3 studies in survey of academic literature.

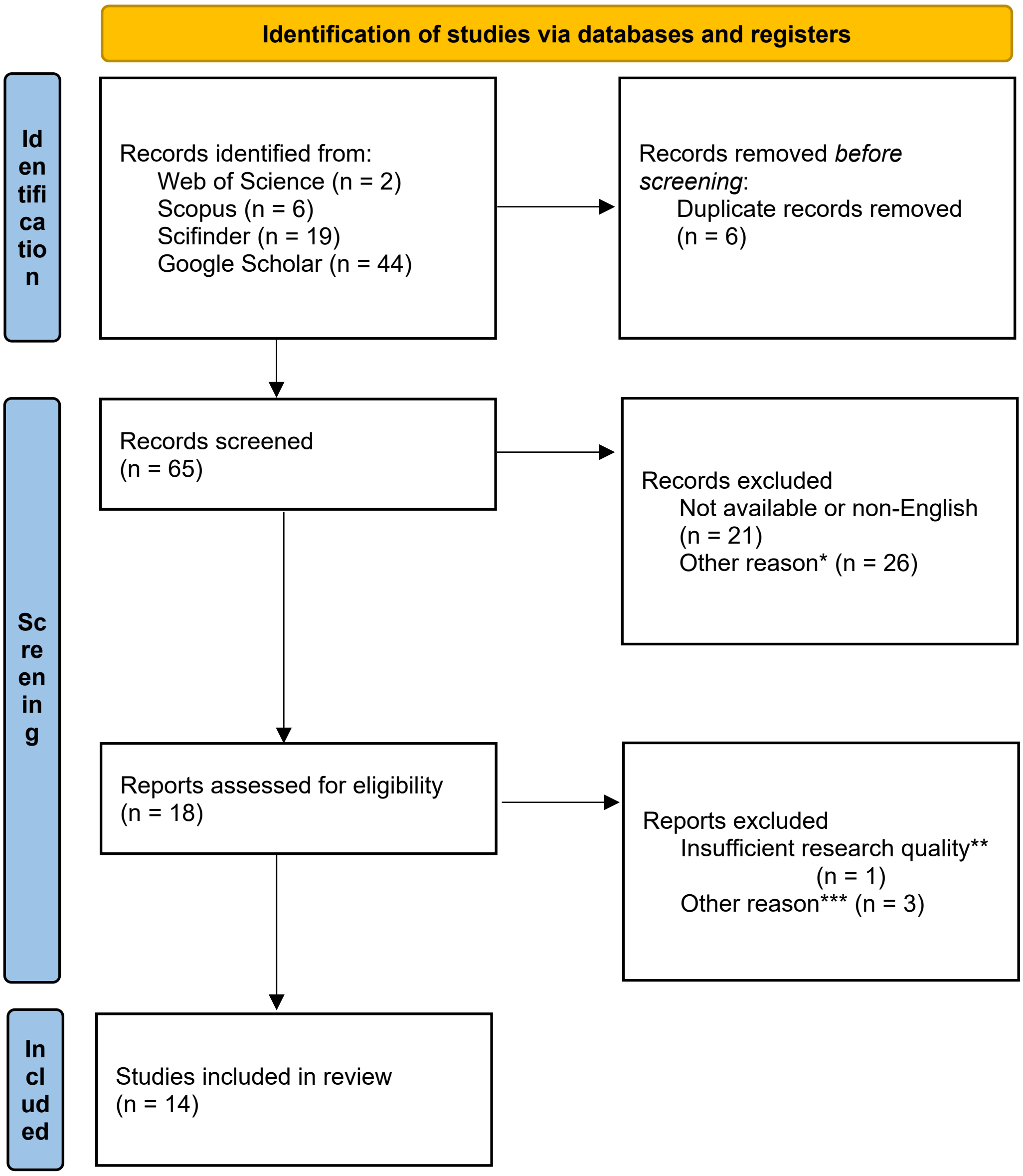

Adapted from: Page MJ, et al. BMJ 2021;372:n71. doi: 10.1136/bmj.n71.

* Reports that focused on battery applications (e.g., remote wireless sensors) or other battery technologies (e.g., alkaline or lithium metal) were excluded in the screening of titles and abstracts.

** Reports which were not trustworthy, either based on suspicion of large reliance on AI or with

concerns of plagiarism or insufficient referencing were excluded in the screening of full texts.

*** Reports without information about material (PFAS or non-PFAS) were excluded in the screening of full texts.

3. SUPPORTING EVIDENCE

In the second part of the study, we collected supporting evidence from multiple source types. The sections below outline each source type and present the corresponding results, which are subsequently integrated into lines of evidence.

3.1. Previous use-mapping studies

In this section, we summarize the results from previous use-mapping studies that relate to PFAS in batteries, including peer-reviewed literature, governmental reports and non-governmental organizations (NGOs) studies. Because of their varying scope, the presentation of each source is adjusted to its content. The studies also reference each other; wherever possible, the data from the original reference is presented. They are presented below in chronological order based on publication date. If several editions are available, we include the first edition publication date.

3.1.1. Kissa 2001

In "Fluorinate surfactants and repellents" Erik Kissa, describes the structure, synthesis and properties of these substances, among other aspects.[14] Kissa includes a chapter on applications of fluorinated surfactants, including a section on *Electronics*. Five specific battery cases are noted:

- zinc being "amalgamated" to prevent hydrogen evolution and electrode corrosion in alkaline and zinc-carbon batteries,
- previously mercury was used, but was already substituted with Forafac 1110,
- fluorinated surfactants added to zinc battery to suppress dendrite formation, also using Forafac 1110,
- a battery with Zn anode, nickel oxide cathode and KOH electrolyte containing Zonyl FSN fluorinated surfactant in the electrolyte, and
- the cathode of a MnO2 battery treated with fluorinated surfactant.

References for all examples included in Kissa (2001) are dated in 1990 or earlier.

3.1.2. Ebnesajjad 2013, Ebnesajjad 2021

In the first (2013)[15] and second (2021)[22] edition of the book "Introduction to Fluoropolymers", Sina Ebnesajjad discussed the history, technology and applications of fluoropolymers.

In the first edition, it is reported that fluorinated graphite (denoted as CFx) is used as an additive for lubrication and in lithium batteries, stating that "In primary lithium batteries, the high affinity of the graphite fluoride to lithium renders them desirable as cathode materials. The low equivalent weight of the carbon and fluorine elements allows achievement of a high-specific energy density." When discussing PVDF, Ebnesajjad reported that "PVDF homopolymers and copolymers are used in the battery industry as binders for cathodes and anodes in lithium-ion batteries, and as battery separators in lithium-ion polymer batteries."

This is further elaborated in the second edition, describing that PVDF is applied in cathode, anode, and separators. Ebnesajjad (2021) notes that "formulations using emulsion PVDF have higher surface areas, making dissolution into NMP or acetone faster."

3.1.3. Ameduri 2018, Ameduri 2020

In 2018, Ameduri published the article "Fluoropolymers: The Right Material for the Right Applications".[13] In general, Ameduri reports that fluoropolymers can be used in lithium, sodium, and fluoride batteries and as fluorinated additives for electrolytes. For example, it is stated that triethylene glycol methacrylate copolymerized with vinylidene fluoride has been synthesized for a gel polymer electrolyte in Li-ion batteries. Furthermore, it is reported that gel or solid polymer electrolytes as well as separators are applied in Li-ion batteries. In "The Promising Future of Fluoropolymers", published 2020, Ameduri repeats the same claim.[23]

3.1.4. Nordic Council of Ministers 2019

In 2019, Nordic Council of Ministers released a report on "Per- and polyfluoroalkylether substances: identity, production and use", which report two uses of PFAS in batteries. This included flow batteries (outside the scope of this paper) and a side-chain perfluoroether polymers used in mixtures (Fluorolink AD1700) used in "battery systems in automotive sector".[24]

3.1.5. Glüge et al. 2020

In 2020, Glüge et al. published an overview of a wide range of uses of PFASs, function, and volumes, where information was available.[4] A total of 200 use categories were identified. Among the many sources, Glüge et al. refers to Kissa (2001) and Ameduri (2018). Other sources included other peer-review articles, and patents, among others.

In the Appendix of Glüge et al. (2020), the uses of PFASs and properties employed are outlined(Table S3).

Table S3. Uses listed in Glüge et al. (2020) (Table 4. "Overview of the uses of PFAS, the function of the PFAS in the uses and the properties of the employed PFAS that make them valuable for this application") within the scope of our study.

Subcategory	Function of PFAS	Properties of the PFAS employed
Lithium batteries	Binder for electrodes	Almost no reactivity with the electrodes and electrolyte
Lithium batteries	Prevent thermal runaway reaction	Good heat absorption of first layer and good heat conductivity of second layer
Lithium batteries	Improve the oxygen transport of lithium–air batteries	Great capacity to dissolve gases
Lithium batteries	Electrolyte solvents for lithium–sulfur batteries	Bipolar character of some of the PFAS
Zinc batteries	Prevent formation of dendrites, hydrogen evolution and electrode corrosion due to adsorption onto the electrode surface	Low surface tension, non-reactive
Alkaline manganese batteries	MnO2 cathodes containing carbon black are treated with a fluorinated surfactant	?

In the electronic supplemental information ESI-1, Glüge et al. (2020) describes uses categorized within the Energy sector, in which the section 1.8.2. Energy storage is of interest to this work. They classify batteries into the subcategories: "Lithium-ion battery", "Zinc batteries" and "Alkaline manganese batteries". They also report on the use in vanadium redox batteries as well as electrolyzer/fuel cells, but these are not included in scope of this work.

First, Glüge et al. (2020) report that "PVDF and FEP are used as binders" for both negative and positive electrodes and the function is to "join the active material and conductive additives together without reacting with the electrodes and electrolyte" in Li-ion batteries. Other uses noted in Glüge et al. (2020) include e.g., the use of a fluorocarbon resin as heat absorbing layer in the inside of the case of the battery pack, a lithium battery from a Sony Ericsson cell phone analyzed to contain perfluorononanoic acid, 6:2 fluorotelomer sulfonic acid, n-methyl and n-

ethyl perfluorooctane sulfonamidoethanols, and "PFAS patented as electrolyte solvents" for lithium-sulfur batteries. In addition, Glüge mention the use of PFAS-based heat transfer fluids for immersion cooling and a PFAS additive to improve the oxygen transport of lithium-air batteries.. It is however unclear whether they specifically refer to uses in Li-ion batteries, as for example the use of fluorocarbon resins and fluoropolymers are reported for "lithium batteries", which could also be of primary type. Furthermore, solid-state batteries with e.g. a gel polymer electrolyte are described to consist of a polymer matrix made out of vinylidenfluorid-hexafluorpropylen copolymer.

Furthermore, Glüge et al. (2020) describe uses of PFAS in zinc batteries. Here, it is stated that fluorinated surfactants could be used in the electrolyte, including poly(oxy-1,2-ethanediyl), α-(3,3,4,4,5,5,6,6,7,7,8,8,8-tridecafluorooctyl)-ω-hydroxy- (Forafac 1110, CAS No. 52550-44-4), exemplified for alkaline and zinc-carbon batteries, citing Kissa 2001. In addition, PTFE and PVDF are reported as binders for the electrodes.

Lastly, Glüge et al. (2020), state that "Alkaline manganese batteries have been made with MnO2 cathodes containing carbon black treated with a fluorinated surfactant (e.g., potassium perfluoroalkylcarboxylate)", referencing Kissa (2001).

Most of the above-discussed substances are also listed in their electronic supplemental information 3 (ESI-3), which we list in Table S4.

Table S4. Reported uses of PFAS in the energy sector in Glüge et al. (2020) (ESI-3. "Energy Sector") within the scope of our study.

CAS No.	Name	Elemental composition	Use Type	PFAS Type
24937-79-9	Poly(vinylidene fluoride)	(CH2CF2)x	U	polymer
25067-11-2	Fluorinated ethylene propylene	(CF2CF2)x-(CF2CFCF3)y	U	polymer
9002-84-0	Polytetrafluoroethylene	(CF2CF2)x	U	polymer
375-95-1	Perfluorononanoic acid (PFNA)	C8F17COOH	D	non-polymer
27619-97-2	6:2 Fluorotelomer sulfonic acid (6:2 FTS)	C6F13CH2CH2SO3H	D	non-polymer
24448-09-7	N-Methyl perfluorooctane sulfonamidoethanol	C8F17SO2N(CH3) CH2CH2OH	D	non-polymer
1691-99-2	N-Ethyl perfluorooctane sulfonamidoethanol	C8F17SO2N(C2H5) CH2CH2OH	D	non-polymer
122193-68-4	Oxirane, 2-[[(3,3,4,4, 5,5,6,6,7,7,8,8,8-tridecafluorooctyl)oxy]methyl]-	C6F13CH2CH2O CH2C2OH3	U	non-polymer
97571-69-2	1,4,7,10,13-Pentaoxacyclopentadecane, 2,2,3,3,5,5,6,6,8,8, 9,9,11,11,12,12,14, 14,15,15-eicosafluoro-	(CF2CF2O)5	P	non-polymer
21297-65-4	1,3-Dioxolane, 2,2,4, 4,5,5-hexafluoro-	CF2OCF2CF2O	P	non-polymer
773-14-8	Furan, 2,2,3,3,4,4, 5,5-octafluorotetrahydro-	OCF2CF2CF2CF2	P	non-polymer
64028-	2,5,8,11,14-Penta	CF3O(CF2CF2O)4CF	P	non-

06-4	oxapentadecane, 1,1,1,3,3,4,4,6,6,7 ,7,9,9,10,10,12,12,13,13, 15,15,15-docosafluoro-	3		polymer
40891-99-4	Ethane, 1,1′-oxybis[1,1,2,2-tetrafluoro-2-(trifluoromethoxy)]-	CF3O(CF2CF2O)2CF3	P	non-polymer
52550-44-4	Poly(oxy-1,2-ethanediyl), α-(3,3,4,4,5,5,6,6, 7,7,8,8,8-tridecafluorooctyl)-ω-hydroxy-	C6F13CH2CH2O (CH2CH2O)mH m = 10 to 20	U	unclear

3.1.6. OECD Fact cards 2022

In the Series on Risk Management No. 68, OECD Environment, Health and Safety Publications included a set of 15 fact cards on major groups of per- and polyfluoroalkyl substances (PFASs), which were prepared by the OECD/UNEP Global PFC Group between June 2018 and June 2021.[25] In this report, OECD report that short-chain PFSAs are used as esterification catalysts, e.g. in the form of their lithium salts in batteries. In addition, they list Fluorolink AD1700 as an additive in battery systems, citing the Nordic Council.

3.1.7. Gaines 2022

In 2022, "Historical and current usage of per- and polyfluoroalkyl substances (PFAS): A literature review" by Linda G.T. Gaines was published.[9] It includes an overview of uses of PFASs and a total of 25 use categories are presented. The sub-use of batteries is included in electronics section and Gaines divides the results in "Lithium batteries" and "Zinc batteries" (Table S5).

Gaines states that the battery industry use PVDF as "binders for cathodes and anodes in lithium-ion batteries" and as "battery separators in lithium-ion polymer batteries" (citing Ebnesajjad (2013)). Gaines also reports that "Alkaline manganese batteries may have MnO2 cathodes treated with PFAS" (citing Kissa (2001)).

Table S5. Uses listed in Gaines (2022) within the scope of our study.

Use (category/ subcategory)	Description	Substance	Properties/Functions
Battery industry, lithium-ion batteries	Binders for cathodes and anodes	PVDF	
Battery industry, lithium-ion polymer batteries	battery separators		
Lithium batteries	binder on the positive and negative electrode current collector/ electrolyte solution	PVDF / specific perfluoro group-containing compound ("The additive has fluorine-substituted ethers, amides, esters, carbonates, phosphate esters, and phosphates")	allows for safety and high battery characteristics
Lithium battery	electrolyte additive	perfluoro nitrile compound	
Lithium battery (Li-air batteries;	electrolyte additive		can limit electrolyte loss and stabilize the

and other)			solid-electrolyte interphase
lithium-ion batteries	electrode binders	fluorinated polymers	
nonaqueous electrolyte battery	positive-electrode active material	fluorinated graphite	
storage batteries or batteries, such as lead storage batteries and Edison storage batteries.	additive to the electrolyte	PFSA surfactants	lowers the surface tension and allows for rapid and complete wetting-out and penetration by the electrodes' electrolytes.
Zinc battery	Electrolyte	PFAS	Mercury used to be used in zinc batteries to decrease the rate of hydrogen evolution, but fluorinated surfactants can be substituted for mercury.
Alkaline manganese batteries	MnO2 cathodes	treated with PFAS	
zinc battery		Zonyl FSN (DuPont brand)	scale inhibitor

3.1.8. Annex XV 2023, Background Document 2025

In February 2023, five European countries (dossier submitters) submitted an Annex XV report to ECHA proposing to restrict PFAS. After a public consultation, it was updated in 2025 and included as the Background Document to the expert committees.[10,11]

3.1.8.1. Uses

In Annex A, the dossier submitters describe uses of PFASs, in which batteries are found in the Energy sector. They list chemical properties, related to all of Energy industry: “Chemical/thermal resistance, ion transportation, high weatherability, high transparency, corrosion resistance, oleophobic, hydrophobic, low surface tension, stable, non-reactive, acid gas scrubber, heat absorption, conductivity, capacity to dissolve gases, bipolar, resistance to acids, and highly oxidizing species, wettability, heat conductivity, high dielectric strength, low global warming potential, forms no residue, dirt repellence, high vapour barrier, high transparency, particular and chemical filtration.”[26,27] In the Background Document, this is complemented with a description that “PFASs are used in the energy sector primarily due to their stability, durability, barrier properties, thermal and chemical resistance along with electric isolation.”[27]

A full list of PFAS uses relevant to our study is included below in Tables S6-S8.

Table S6. Uses of PFAS in batteries listed in the Annex XV report, Annex A (Table A.53. “PFASs uses in the energy industry – literature and publicly available sources, complemented by a stakeholder”) within the scope of our study.

**Energy facility/unit**	**Use as/for**

Lithium batteries	Binder for electrodes, prevent thermal runaway reaction, oxygen transport, electrolyte, sealing
Vanadium redox batteries	Ion exchange membrane
Zinc batteries	Prevent formation of dendrites, hydrogen evolution and electrode corrosion due to adsorption to electrode
Alkaline batteries	Surfactant
Battery systems	Cooling

Table S7. Uses and applications of PFAS in the Annex XV report, Annex A (Table A.54. "Identified uses and application of PFASs in the energy industry identified by stakeholders") within the scope of our study.

**Use category**	**Sub-use**	**Properties**	**Area of use/ application(s)**	**Examples of PFAS**
Lithium-ion batteries	Seals,<br>electrode binders,<br>separator films/coatings,<br>electrolyte additives,<br>thermal management pack/module	• Cooling<br>• Electric stability	Use to contain aggressive electrolytes	Fluoroelastomer, PVDF
Batteries	Battery fluid,<br>Compounds for separator films,<br>Binder		Rechargeable batteries	

Table S8. Uses of PFAS in batteries as reported in the Background Document, Annex A (Table A.57. "Identified uses and application of PFASs in the energy industry identified by stakeholders") within the scope of our study.

Use category	Sub-use	Properties	Area of use/application(s)	Examples of PFAS
Batteries (Primary64 / Secondary65)	Binders in active material masses	• Conductive properties<br>• Hydrophobic<br>• Adhesive<br>• Corrosive resistance<br>• Mechanical properties<br>• Thermal properties<br>• Dispersive capabilities<br>• Chemical and Electrochemical stability	PVDF use in binder: Used to disperse active material and conductive additives to enable homogeneous distribution of slurry, also modify viscosity. Adhesion active material, conductive additives and collector.<br>PTFE use in binder: Mechanical cohesion between positive electrode particles to enable electrode integrity, lubricant for electrode particles to give uniform electrode density for performance and longevity, lower water absorption during mixing.<br>Used in: Li-ion (wet and dry), Na-ion, Solid-state LMP, Ni-based rechargeable batteries, Primary- Li-SOCl2, LI-SO2, Li-MNO2, Zn-air, Silver oxide, lithium metal rechargeable.<br>PFAS is used in all positive electrode except for solid-state LMP, in which it is used in the electrolyte. PFAS is also used in negative electrode for Li-ion (wet and dry), Na-ion and Ni-based rechargeable batteries.<br>Rechargeable batteries: Lithium-ion, lead-acid, nickel-metal hybrid and nickel-cadmium.	Polymeric PFAS: PTFE and PVDF
	Electrolytes	• Stability<br>• Thermal operating range	Increase electrolyte stability; capturing water and avoid hydrogen fluoride emissions. Lithium-ion rechargeable, Lithium primary, Lithium metal rechargeable, and Sodium-ion rechargeable batteries<br>Lithium manganese dioxide (Primary) has a high use in coin cells and cylindrical cells for consumers and industrial application.	Non-polymeric PFAS: Lithium salts e.g. Li-Triflate, LiTFSI, LiBETI, LiFAP, LiTDI<br>Additives e.g. Tris(2,2,2-trifluoroethyl)borate, Trifluorotoluene, Sodium bis(trifluoromethyl-sulfonyl) imide

				1,1,2,2- Tetrafluoroethyl 2,2,3,3-tetrafluoropropyl ether
	Separator coatings	• Thermal stability<br>• Mechanical stability<br>• Adhesion	PVDF is used as a coating on a separator, which ensure separation of negative and positive electrode.	Polymeric PFAS: PVDF
Lithium-ion batteries (Prismatic, cylindrical and pouch) (secondary)	Electrode binders (cathode and anode), separator, films/coatings, Polymer electrolyte, electrolyte additives, thermal management pack/module	• Cooling<br>• Electric stability<br>• Electrical insulation and sealing from air and moisture.<br>• Dispersive capabilities<br>• Chemical stability<br>• high heat resistance<br>• Oxidation resistance<br>• Pressure<br>• Water- and oil repellency<br>• Mechanical properties<br>• Adhesive<br>• Chemical resistance	Rechargeable. Electric Vehicle (battery electric vehicles (BEV), plug-in hybrid electric vehicle (PHEV), hybrid electric vehicle (HEV), and fuel cell electric vehicle (FCEV)<br>Energy Storage System<br>Battery packs / portable power e.g. powerbanks, smartphone, PC,<br>Power Tool<br>Use to contain aggressive electrolytes and maintain strong connection between electrode and current collectors.	Polymeric PFAS: Fluoroelastomer, PVDF, PFA, PTFE<br>Copolymers for PVDF: VDF, TrFE, TFE, CTFE, HFP<br>Non-polymeric: LiTFSI, LiTFS
Primary lithium batteries / CR66 / BR67 / Coin / cylindrical	Positive electrode binder<br>Electrolyte	• Mechanical strength<br>• Heat resistance<br>• Oxidation resistance<br>• Chemical resistance<br>• Electrochemical stability	Non-rechargeable.<br>Smoke Alarm for Homes,<br>Vehicle Emergency Call, AED<br>Tyre Pressure Monitoring, Smart Key, Blood Glucose Meter, IoT	Polymeric PFAS: PTFE, FEP, PFA,<br>Non-polymeric PFAS: LiTFS, LiTFSI
Coin lithium rechargeable (secondary)	Positive electrode binder, gasket, electrolyte	• Mechanical strength<br>• Heat resistance<br>• Oxidation resistance<br>• Chemical resistance	Rechargeable.<br>Earphone, Watch, Hearing Aid, IoT	Polymeric PFAS: PTFE, ETFE, FEP, PVDF, PFA<br>Non-polymeric PFAS:

		• Electrochemical stability<br>• Water repellence		LiTFSI
Nickel metal hybrid battery/nickel cadmium battery (secondary)	Positive electrode binder<br>Negative electrode	• Oxidation potential<br>• Decrease internal pressure<br>• fibrillation properties<br>• Chemical stability<br>• hydrophobic	Rechargeable. Electric Vehicle,<br>Energy Storage System<br>Consumer Use; Alarm systems, Electronic devices, Emergency lights, Memory Back Up (MBU), GPS terminal, Smart home applications	Polymeric PFAS: PTFE, poly(propylene-co tetrafluoroethylene, FEP, PFA
Lithium thionyl chloride battery (ER) (primary)	Positive electrode binder	• Porous electrode<br>• Heat resistance<br>• Oxidation resistance<br>• Chemical stability<br>• Mechanical strength	Non-rechargeable.<br>Smart Meter, Military Use	Polymeric PFAS: PTFE
Alkaline button (LR) (primary)	Positive electrode binder	• Oxidation resistance<br>• Chemical resistance<br>• Electrochemical stability<br>• Binding properties	Non-rechargeable.<br>Toy, Thermometer, Game	Polymeric PFAS: PTFE
Solid-state batteries	Electrolyte, cathode, binder	• Conductivity<br>• Stability<br>• Mechanical strength	Lithium-metal-polymer, ceramic sulfide, ceramic oxides batteries.	Polymeric PFAS:<br>PVDF and PTFE<br>Non-polymeric PFAS:<br>LiTFSI, TFSI
Zinc-air batteries	Binder in active material	• Hydrophobic<br>• Air permeability<br>• fibrillation properties<br>• Chemical stability	Medical devices like hearing aids and hearing/cochlea implants	Polymeric PFAS: PTFE
Lithium iron phosphate	Electrode binder	Covered under lithium-ion batteries	Polymeric: PVDF	Lithium iron phosphate

Silver oxide (SR)	Binder in active material	• fibrillation properties<br>• Chemical stability<br>• hydrophobic	Blood sugar indicator, Insulin pump, Smart Capsules, Watch (digital & analog)	Polymeric PFAS: PTFE
H2-gas generation battery	Binder in active material	• Hydrophobic<br>• Air permeability<br>• fibrillation properties<br>• Chemical stability	H2-gas generation battery	Binder in active material

The Annex XV report stated that Li-ion batteries have sub-uses of “seals, electrode binders, separator films/coatings, electrolyte additives, thermal management pack/module” with fluoroelastomer and PVDF as examples. Similarly, in the Background Document additional substances, such as PFA, PTFE, copolymers of PVDF, LiTFSI and LiTFS were examples for these sub-uses (with a few additions) of Li-ion batteries.

. In the Annex XV report, “electrode binders” are mentioned among other sub-uses for Li-ion batteries, but it is not possible to distinguish which substance is used in which sub-use because of the grouping. This is updated in the Background Document to “electrode binders (cathode and anode)” with more examples of PFASs, but still not distinguishable between Battery Use Levels 2.1-2.3 The Background Document state that “the binders in active masses” in “Batteries (Primary / Secondary)” has properties such as adhesive and mechanical, thermal and corrosive properties are linked to example of PFASs of PTFE and PVDF. In “nickel metal hybrid battery/nickel cadmium battery (secondary)” the use of several fluoropolymers is linked to use in positive electrode binder and negative electrode.

Similarly, in the Background Document describing the use of “Batteries (primary / secondary)”, the sub-use of “separator coatings” is linked to the functions of thermal stability, mechanical stability and adhesion, stating that “PVDF is used as a coating on a separator which ensure separator of negative and positive electrode” claiming the use of PVDF. However, the use of PFASs in “separator films/coatings” in Li-ion batteries is reported together with a wide range of PFASs and sub-uses.

In the Annex XV report, primary batteries are not mentioned. In contrast, the Background Document states the use of “PTFE, FEP, PFA, LiTFS, and LiTFSI” as examples in “Primary lithium batteries / CR / BR / Coin / Cylindrical” for the positive electrode binder and electrolyte. The Background Document also reports that PTFE is used as binder active material/positive electrode in “silver oxide (SR)” battery, “Alkaline button (LR) (primary)” battery, “lithium thionyl chloride battery (ER) (primary)”, “zinc-air batteries”

No non-polymeric PFASs were mentioned in the Annex XV report, but several are stated for electrolytes in batteries in the Background Document. Grouped uses of polymeric and non-polymeric PFASs are listed for “coin lithium rechargeable (secondary)” batteries and “solid-state batteries”. Battery fluids were mentioned in the Annex XV report, but not in the Background Document.

3.1.8.2. Alternatives

In Annex E of the Annex XV report and the Background Document, the dossier submitters list available alternatives.[28,29] In this section, we focus on the Background Document as this also includes information from the Annex XV report consultation.[27] In general, they report that for most uses the comments provided no evidence that alternatives are available. They state that binders for batteries are not likely to be available within a short timeframe (strong evidence), electrolyte alternatives could be available for some Li-ion batteries (conflicting evidence) and that alternatives are available in development phase for separator coatings (uncertainty regarding to technical feasibility). The dossier submitters provide information in several tables and in subsections on alternatives for the use in batteries, which is summarized below in Table S9.

Table S9. Alternatives for PFAS use in batteries reported in the Background Document, Annex E (Table E.224. “Information on alternatives for the use in batteries”) within the scope of our study. Only information related to substance identity and conclusions is included here.

<table>
<tr><th>Use</th><th>Sub-use</th><th>Information on alternatives</th></tr>
<tr><td>Batteries (Primary, Secondary)</td><td>Binders</td><td>Anode (research stage): lithium, gelatin (Li-CMC), sodium alginate, xanthan gum, chitosan and derivate, agar-agar, carrageenan, guar gam, and many other polymers as PAA, PMA, PVA, AMAC, AMMA, polyimide, carboxymethyl cellulose (CMC), and styrene-butadiene rubber (SBR).
Unspecified (with limitations): SBR, PAA, cellulose, rubbers, polyolefins, HNBR, ACM, aliphatic hydrocarbon polymers, aliphatic hydrocarbon polymers, PVA) , polyolefins, CMC, cellulosic resins such as hydroxypropyl cellulose (HPC), polyacrylonitrile (PAN), polyimide (PI), Polynitriles nickel, NMP.
“In conclusion, feedback from many stakeholders shows that alternatives are not available at present, but that binders for some applications are in development and likely to be feasible in the future.”</td></tr>
<tr><td rowspan="3">Lithium-ion batteries</td><td>Electrolytes</td><td>General (with limitations): LIPF6, lithium hydroxide, lithium carbonate, lithium fluoride, lithium sulfate, lithium nitrate, lithium acetate, Lithium perchlorate Solid-state batteries (LMP) ceramic sulfide and ceramic oxides.
“In conclusion, there is sufficient evidence to conclude that alternatives are not feasible in this use, and further research is likely to be required. It is likely that alternatives are not feasible for use in electric vehicles, whereas for batteries with fewer requirements, alternatives might be feasible.“</td></tr>
<tr><td>Separator films/ coatings</td><td>General (with limitations): binder-free, think-film ceramic coated separators.
Li-S batteries: acrylate-based separators.
“In conclusion, stakeholder input suggests that alternatives are not yet available, but are being developed, and more time is still required before they can be implemented.”</td></tr>
<tr><td>Whole replacement of batteries</td><td>“In conclusion, there may be some applications where other battery types that are PFAS-free can replace lithium-ion batteries, but they are not feasible alternatives in all applications of lithium-ion batteries and in most cases the alternatives were also PFAS based.”</td></tr>
<tr><td rowspan="2">Primary lithium batteries / Coin / cylindrical</td><td>Binder</td><td>See Battery (primary, secondary).</td></tr>
<tr><td>Electrolyte</td><td>General (with limitations): lithium hydroxide, lithium carbonate, lithium fluoride, lithium sulfate, lithium nitrate, lithium acetate, lithium hexafluorophosphate, lithium perchlorate.
“In conclusion, the limited stakeholder input for this battery type suggests that alternatives are likely not feasible at the moment.”</td></tr>
<tr><td>Coin lithium rechargeable (secondary)</td><td>Binder</td><td>See Battery (primary, secondary).
“In conclusion, the limited stakeholder input for this battery type suggests that alternatives are likely not feasible at the moment.”</td></tr>
</table>

Nickel metal hybrid battery/Nickel cadmium battery (secondary)	Binder	See Battery (primary, secondary).
Li thionyl chloride battery (primary)	Binder	See Battery (primary, secondary).
Alkaline button (SR / LR) (primary)	Binder	See Battery (primary, secondary).
Solid-state batteries	General	"In conclusion, the available evidence suggests that further research is required for both the application itself, as well as PFAS-free alternatives for potential uses of PFAS within this application."
	Binder	See Battery (primary, secondary).
	Separator	See Lithium ion battery, separator coating.
Zinc-air batteries	Binder	See Battery (primary, secondary).
Nickel-metal hydride	Binder	See Battery (primary, secondary).
Silver oxide batteries	Binder	See Battery (primary, secondary).
H2-gas generation battery	Binder	See Battery (primary, secondary).

3.1.9. ChemSec April 2023

The report "Check Your Tech" was written by WSP for Chemsec in April 2023 and describes the uses of PFASs in electronics industry.[18]

Li-ion batteries are mentioned as one category of PFASs in electronic devices. Here, it is stated that PVDF is used as a binder and as a separator. Furthermore, they note that novel battery technologies could use fluoropolymers as electrolytes. In addition, perfluorocarbons are noted to be used in "applications requiring the separator of high voltage components" such as electric car batteries.

The report also contains a guide, which lists the uses of PFASs and alternatives. A total of four entries is linked to Li-ion batteries. They list PI, PAA, CMC and SBR as alternatives for the binder, but further note that no alternatives were identified for the cathode electrode binder material. $LiPF_6$ is reported as an alternative in the electrolyte, while no alternatives are identified for the battery separator material.

3.1.10. Rensmo et al. 2023

The literature review "Lithium-ion battery recycling: a source of per- and polyfluoroalkyl substances (PFAS) to the environment?" by Rensmo et al. (2023) aimed to summarize the uses

of fluorinated substances in Li-ion batteries, and the potential for PFASs to be released to the environment during Li-ion battery recycling.[16]

Fluorinated substances, including PFASs, were identified in electrodes and binder, electrolyte (main components and additives), and separator by Rensmo et al (2023). First, the review reports that PVDF could be used in binders of anodes, although more commonly SBR and CMC are used. Similarly, the separator could be of PVDF or PVDF-HFP, but PP or PE are more common. The cathode often contains a fluorinated binder, such as PVDF, PVDF-HFP, PVDF-TrFE, PTFE, FEP as well as a fluoroacrylate polymer under the trade name TRD202A.

Many fluorinated substances were reported as constituents in the electrolyte, of which Li-triflate, LiTFSI, LiBETI, LiFTFSI are falling within the PFAS definition. In addition, some novel or advanced substances, only reported for lab-scale research, included perfluoroalkyl carboxylates (PFCAs), perfluoroalkyl phosphinates and perfluoroalkyl disulfonates as well as perfluorinated analogues of PF_6 (e.g., lithium tri(pentafluoroethyl)triphosphate (LiFAP)), organoborates, organophosphates and organoaluminates. In addition to electrolyte salts, the study reports trifluoropropylene carbonate and tetrafluoroethyl tetrafluoropropylether as PFAS-based electrolyte solvents.

3.1.11. Danish Environmental Protection Agency 2024

In January 2024, the Danish Environmental Protection Agency (Danish EPA) published their report “PFASs and PFAS free alternatives in the Electronics and Energy sector: A summary”, edited by Royal Haskoning DHV.[19] They note that “data that was collected for the electronics, semiconductor, and energy sectors from literature, the call for evidence and the targeted consultation is presented.”

They estimated volumes of PFASs in batteries including polymers (2 600 – 3 000 tonnes/year) and non-polymers (250 tonnes per year), for which “volume of PVDF can be expected to increase from 1 500 tonnes in 2020 to 16 500 tonnes in 2030.”

Particularly, they list identified uses within batteries in two categories: Lithium-ion batteries (Seals, electrode binders, separator films/coatings, electrolyte additives, thermal management pack/module) and Batteries (Battery fluid; Compounds for separator films and Binder), the same categories as described by the Annex XV report. They report that hydrocarbon elastomers could be non-PFAS alternatives for seals, but report that stakeholders perceive these to lack of resistance and other properties that would lead to failure. In addition, they report that solid-state or lead acid batteries could be alternative technologies while acknowledging stakeholders who reported that these could have reduced energy efficiency and could not cover the expected growth of electric vehicles. In regard to the latter category (“Batteries”), they report that there are several non-PFAS alternatives for the anode binder, including “Carboxymethyl cellulose lithium, gelatin (Li-CMC), sodium alginate, xanthan gum, chitosan and derivate, agar-agar, carrageenan, guar gam, and many other polymers as PAA, PMA, PVA, AMAC, AMMA, polyimide, carboxymethyl”. They note that stakeholders report “Possibly better performing and more environmentally friendly alternatives, but largely at research stage.”

3.1.12. The United States Department of Energy 2024

In January 2024, the United States Department of Energy (US DOE) released a technical report titled “Assessment of Fluoropolymer Production and Use With Analysis of Alternative Replacement Materials”.[21] In general, they report that fluoropolymers have wide use in transport and energy sectors, such as in batteries. All uses relevant for our study are outlined in Table S10.

Table S10. Uses of PFAS in batteries referenced in the US DOE report within the scope of our study.

Use (category/ sub-category)	Description	Substance	Source in source
Lithium-ion battery	Binder in electrode	PVDF	(Zhong et al., 2021); (Lahiru Sandaruwan et al., 2022)
No information	Facing layer for battery management system	fluoropolymers	
Lithium-ion battery	Facing layer material in components and systems to manage thermal runaway issues in electric vehicle batteries	fluoropolymers	
Lithium-ion battery	Anode and cathode binder	PTFE*	(Lingappan et al., 2021).
Lithium-ion battery	Electrode binders	PVDF, PVDF-HFP,	(Lingappan et al., 2021). (Wang et al., 2018). (Stephan et al., 2006; Amin-Sanayei and He, 2015).
Lithium-ion battery	Membrane, battery pack	Unknown	
Lithium-ion battery	Separators	PVDF, PVDF-HFP, PVDF-CTFE, and PVDF-TrFE	(Costa et al., 2013).
Lithium-ion battery	Gel polymer electrolytes	PVDF, PVDF-HFP	(Zhang et al., 2014; Liang et al., 2018; Liu et al., 2021).
	Separator coatings	PVDF, PTFE	

* Historical use (e.g. 1980)

In the description of PVDF use as binder in Li-ion battery electrode, the report states that "providing mechanical strength, flexibility, and thermal stability features over its operational life". In other cases, no information was given related to the specific function of the substance, only either properties of the substance or the use requirements.

3.1.13. Savvidou et al. 2024

A perspective article titled "PFAS-Free Energy Storage: Investigating Alternatives for Lithium-Ion Batteries" was published by Savvidou et al. in 2024.[17] They outline alternatives tested in lab-scale as well as companies that offer or are developing PFAS-free materials for electrode binders and electrolytes, primarily salts. The authors report that PVDF and PTFE are commonly used as binders in Li-ion batteries and gel polymer electrolytes in solid state batteries. $LiPF_6$ is most commonly used in Li-ion battery electrolytes, with LiTFSI as an additive (<10%) to improve cycling and lifetime.

Lab-scale alternatives presented by Savvidou et al. (2024) included e.g. Lotader 5500 (polyethylene-co-ethyl acrylate-co-maleic anhydride thermoplastic elastomer) for coin cell batteries with LFP and LTO chemistries. PFAS-free conducting and bio-based binders have also been investigated. Furthermore, binders based on sodium or lithium phosphates and silicates have been reported. Liquid electrolytes containing LiBOB salt, biomass derived electrolytes or lithium perchlorate and solid electrolytes using PEO were also discussed.

Reported companies that offer PFAS-free binders included Leclanché (water-based binders), Nanoramic (nanocarbon binder), 24M (binder-free) and FREYR (binder-free) as well as the research institute ZSW (water-based). E-Lyte were stated to provide PFAS-free electrolytes, but with unknown composition. Altris, a battery manufacturer of Na-ion batteries, offers a technology based on Prussian white material with NaBOB as electrolyte salt.

3.1.14. OECD Synthesis reports 2022, 2024 and 2025

In the Series on Risk Management of Chemicals, the OECD published several synthesis reports outlining the lifecycle including production use and emissions, among other aspects of polymeric PFASs. These include side-chain fluorinated polymers (SCFP, 2022)[30], perfluoropolyethers (PFPE, 2024),[31] and fluoropolymers (FP, 2025).[20]

Only one mention of batteries was identified in the two first reports, where the PFPE report mentioned heat transfer fluids for batteries (citing Glüge et al. (2020)).[31]

However, in the FP report, the OECD included an overview of the sector “Energy” in which batteries were discussed.[20] The report primarily referenced one comment to the Annex XV public consultation, namely Battery Association of Japan (BAJ; #4331) (Table S11). In addition, two other Annex XV consultation attachments were reported, including Chemours (#7427) which provide a high-level illustration of the use of fluoropolymers in e.g. energy industry, and the study by Korzeniowski et al. (2023) in which a table present the use of “PVDF homopolymer, PVDF copolymer, THV = TFE-HFP-VF2, FKM, amorphous, ionomer” in “Renewable energy / Energy storage”.

Table S11. Uses of PFAS in batteries listed in the OECD Synthesis report 2025 report within the scope of our study. [a]Refering to a “filler needed for battery binder”

Use (category/ subcategory)	Description	Substance	Reference
Energy/ lithium-ion batteries	module parts	PTFE materials	BAJ
Energy/ lithium-ion batteries	gaskets	PFA copolymers	
Energy/ nickel metal hydride battery and nickel cadmium battery	positive electrode binder	PTFE and TFE-PP polymers (poly(propylene-co-tetrafluoroethylene), CASRN 27029-05-6)	BAJ
Energy/ nickel metal hydride battery and nickel cadmium battery	negative electrode surfaces	powders of fluoropolymers such as PTFE, FEP and PFA	BAJ
Energy/ lithium primary batteries (CR/BR type, cylindrical and coin shapes)	positive electrode binder	PTFE, ETFE and FEP	BAJ
Energy/ lithium primary batteries (ER type)	positive electrode binder	PTFE materials	BAJ
Energy/ coin-shaped lithium rechargeable battery	positive electrode binders	PTFE, ETFE, FEP and PVDF	

Energy/ coin-shaped lithium rechargeable battery	gaskets	PFA copolymers	BAJ
Energy/ lithium-ion batteries	separator; binder	PVDF materials	BAJ
Energy/ [not described]	polymer electrolytes	PVDF polymers	BAJ
Energy/ alkaline button battery (LR and SR)	positive electrode binder	PTFE materials	BAJ
Electrical and electronics/ lithium battery	cases	ETFE copolymers	Kälin et al. (2012)
Not specified / battery [a]	Binder and additives for drip prevention	PTFE (Cas: 9002-84-0; Polyflon D210C)	Daikin (n.d.)
Not specified /lithium-ion batteries	Binder in graphite electrodes	PVDF materials	(Marshall et al., 2021)

3.1.15. Figuière et al. 2025

In 2025, Figuière et al. published a study including a database that documented all known uses of PFASs and described the functions provided by PFASs in these uses. The study also listed potential alternatives and evaluated the suitability of the identified alternatives to replace PFASs.[12]

In this database, a total of 325 PFAS uses were identified and listed. The study list the sub-use "Lithium-ion batteries" the use in the "Energy sector" (51 uses), in the "Electronics and semiconductor industry" and as sub-use (application) in the "Electronics industry". Further duplicates are identified in the use "Transport" in the sub-use "Electrical engineering and industry", in applications "Lithium-ion batteries" and in the sub-use "Sealing applications" ("E.g. Valves, gaskets, piston, electronic devices, battery electrodes"). Substances are repeated for all uses/sub-uses/applications except with the addition of the substance "Perfluoro(methyl vinyl ether)-tetrafluoroethene copolymer" in the sub-use in "Sealing applications".

Listed alternatives are based on the assessment in the Annex XV report and not further elaborated here.

3.2. Input from battery stakeholders

A total of 12 stakeholders along the battery value chain were interviewed. The summary of these interviews, after approval from the organizations, are listed in alphabetical order below. Additional information, such as answers via e-mail as well as anonymous responses, are also included.

3.2.1. Ashland

Ashland is a global manufacturer of products within the additives and specialty ingredients sector, selling to markets that include architectural coatings, construction, energy, food and beverage, personal care and pharmaceutical with customers in more than 100 countries.[32]

An interview was conducted on December 16, 2025, with representatives from *Ashland*. They stated that *Ashland* do not provide any PFAS-based materials and noted that, in the energy sector, they provide materials for the anode, separator and cathode, of various technology readiness level (TRL). Their binder for anode applications is based on carboxy methyl

cellulose (CMC) (brand name bondwell™ for battery applications). The same CMC chemistry can be used for dispersion of conductive additives used in aqueous formulations. CMC is a seminatural product that *Ashland* has modified and is provided in a wide range of grades and viscosities. The representatives noted that the products can be provided with varying molecular weight (Mw) and degree of substitution (DS) to "play around with critical properties". For example, *Ashland* can provide a CMC with a high DS but low Mw providing low viscosity, for hard to disperse carbon nanotubes. In addition to CMC also available is their polyvinylpyrrolidone (PVP) material which can be used as a dispersant in aqueous and solvent based formulations. Dispersants therefore cover both anode and cathode formulations.

Ashland also provides binders for ceramic-coated separators, including the PVP material. This material has "a high molecular weight providing good cohesion within the coating formulation", according to *Ashland*. In addition, they provide the product soteras™ CCS-V, which is non-fluorinated binder. All of *Ashland's* separator-related products are for aqueous processing of the coating of ceramic particles to separators where good thermal stability is required. Furthermore, *Ashland* can provide wetting agents and dispersants for the battery separator coating.

When discussing the timeline for adoption, *Ashland* note that the bondwell™ CMC binders are an existing established technology, with CMC used in the majority of gigafactories in Europe. Development of new binders for the next generation batteries continues however it will take some years before reaching appreciable commercial scale."[33]

3.2.2. Ateios

Ateios was founded 2018[34] and in 2021, they have the exclusive license of a technology from Oak Ridge National Laboratory which innovates the battery manufacturing based on faster production and cheaper products.[35]

An interview was conducted with a representative from *Ateios* on February 19, 2026. They described their solution of a PFAS-free electrode, which is provided to battery manufacturers and OEMs. According to *Ateios*, the electrode manufacturing and material could make up 85% of the total battery cost and performance. In common battery manufacturing processes that use a fluorinated binder called PVDF and NMP solvent, the high cost is largely due to the need for drying after mixing.

Therefore, as *Ateios* described, their RaiCure™ electrode technology uses a readily available short monomer that is cross-linked via electron curing, resulting in a bound matrix of the active material and conductive additives with a cured, high-molecular-weight polymer. As a result, the process does not require a drying step, reducing cost and energy usage. Still, it differs from dry coating, as it can be implemented in current battery production lines using the same wet mixers and coaters, without the need for heat. The representative also noted that this process could provide thicker electrodes, which increase energy density, use cheaper materials that can be bought off the shelf, and avoid fluorinated thermoplastics. Furthermore, recycling could be achieved by using an electron beam at higher energy to reversibly break the polymer back into monomers.

The company has produced electrodes for LFP, LCO, NMC and NCA batteries and their binder is stable for higher voltages (e.g., 4.53 V in LCO cell), according to *Ateios*. The LCO chemistry has been produced in pouch configuration and applied in consumer electronics such as gaming controllers and smartwatches while the LFP chemistry in both cylindrical and prismatic for defense applications and grid energy storage. The NMC batteries have been applied in high-energy or high-power applications and in a variety of configurations depending on application.[36]

Ateios was awarded an National Science Foundation grant for their solvent-free production. In 2025 it was announced that their technology has been demonstrated in a project between *Ateios* and *Kodak*, at coating speeds up to 80 meters per minute.[37]

3.2.3. Celanese

Celanese is a global chemical and specialty materials company, started in Switzerland in 1912.[38] As of early 2026, they have a wide range of products applicable in adhesives, consumer electronics, fluid handling, medical devices, etc.[39]

An interview was conducted November 20 in 2025, with one representative who noted that in the energy sector, *Celanese* provide raw materials (GUR® UHMWPE) for the separators. They are also developing products that could be used for battery electrode binder.[40] This thermoplastic is available in the powder, ranging from "00 to 150 microns", but also in other grades.[41]

In addition, *Celanese* provides a product titled Vamac® AEM. A polar acrylic monomer provides oil and lubricant resistance, while ethylene provides resistance to hydrolysis. Thermal and electrochemical stability of 4.2 to 4.5 V vs Li/Li+ is obtained from the saturated polymer chain, while crosslinking agents are included to adjust the flexibility.

The two products from *Celanese* are not yet implemented in batteries. However, they can reduce the content of PFASs and perhaps in the future replace the PFASs. *Celanese*'s representative noted that there could be battery components that might not have PFAS-free alternatives within a 13.5 years' timeline.[40]

3.2.4. E-Lyte

E-Lyte develops and supplies high-performance electrolyte solutions for advanced energy storage systems, including Li-ion and Na-ion batteries. The company provides customer-specific formulations optimized for defined cell chemistries and performance requirements, supporting reliable integration into large-scale series production.[42]

An interview conducted on December 4, 2025, with a representative of *E-Lyte* highlighted that a majority of the electrolyte formulations developed by the company are PFAS-free. As a customer-driven development partner, the representative noted that *E-Lyte* designs formulations based on specific application and performance requirements. While the commonly used PFAS-based salt LiTFSI offers excellent high-temperature stability, it is frequently not selected for volume applications due to its significantly higher cost (approximately 10 times compared to $LiPF_6$).

Accordingly, *E-Lyte* claimed to focus on delivering technically optimized and commercially viable electrolyte solutions that meet both performance targets and cost expectations of the intended end use. One exception is the need for TFSI in Li-S batteries or in combination with lithium metal electrodes as well as in drilling or electrochromic glasses. The representative noted that LiTFSI is difficult to substitute in these applications. *E-Lyte* currently only provides liquid electrolytes on a commercial scale, but note that some companies look into polymeric electrolytes (e.g. Blue Solutions). For these technologies, LiTFSI might be necessary as they are incorporated within the polymeric electrolyte providing long term stability when operated at elevated temperatures.

When discussing time-line for a PFAS phase-out*, E-Lyte* believes that a transition time of 13,5 years is suitable.[43]

3.2.5. Eneos Materials

Eneos Materials provide a large set of products, ranging from synthetic rubber, thermoplastic elastomers and emulsions. [44] They also provide materials for Li-ion batteries.[45]

An interview was conducted on March 27, 2026, with representatives from *Eneos Materials*. They described that *Eneos Materials* provide styrene butadiene rubber (SBR) to their customers, which are major electric vehicle and battery manufacturers. This material can be combined with carboxymethyl cellulose (CMC) to form a PFAS-free anode.

In addition, the representatives described that some of their customers also use products from *Eneos Materials* in the cathode. Depending on the voltage, *Eneos Materials* provide different solutions for the cathode binder. The SBR material can be used in cathodes of low voltage such as LFP. In addition, *Eneos Materials* also provide special acrylate binders for higher voltages. The representatives recognized larger challenges for high nickel cathodes.

Eneos Materials' products are all water-based binders which not only can be used to substitute PFASs but also to remove the need of NMP in the battery production lines, according to the representatives. As a result, production lines could be much simpler as the NMP collection step would not be necessary. However, it would also be possible to use the water-based binders in the already established production lines as a drop-in solution with minor modifications. For example, the mixing stage would require some optimization.

Overall, the representatives from *Eneos Materials* note that, apart from the anode which is already PFAS-free on the market, several battery chemistries could work with their PFAS-free binders.[46]

3.2.6. GRST

GRST designs and manufactures binder materials for electrodes and market these being PFAS-free and water-recyclable. These binders have been adopted through joint venture and partner OEMs into applications within electric mobility, consumer devices and stationary storage. The binder factory produces at a "ton level (GWh level) scale".[47]

An interview was conducted on December 4 in 2025 with representatives from *GRST*. Their representatives explained that they provide PFAS-free binder solutions at a GWh-scale for battery manufacturers, tailoring the product to their customers' requests. The *GRST* binder material is a functionalized PAA (poly(acrylic acid))-based copolymer with additives to modify its physical and mechanical properties. The material was stated to be compatible with LCO, NMC, NCA and LFP chemistries. Particularly, they stated that their binder is compatible with up to 80% Ni (NMC811) if NMP solvent is used, and up to 60% Ni (NMC622) if water-based process. *GRST* has noted that in some cases, battery manufacturers want to keep the NMP-based process (no change to production line). For both NMP-based and water-based electrode manufacturing process, their binder is PFAS-free and water recyclable.

In general, the representatives noted that integration of a new binder into a battery technology is based on the performance of the battery and the processability of the binder, related to rheology, viscosity and dispersiveness to decrease the risks of peeling, crack or no adhesion. *GRST* claimed their product matches PVDF-binder in terms of capacity, lifecycle, energy density, operating temperature, among other things. In addition, the copolymer binder matches the PVDF in price.

GRST noted that they have been market ready for years now, with a GWh factory. In late 2025, they were in contact with eight OEMs that themselves strive to become PFAS-free. At the time of the interview, *GRST*-binder batteries made by partner OEMs are in the market for electric two-wheelers, and *GRST* binder is in the integration process with the OEMs, which takes 1-2 years.

Overall, the *GRST* representatives stated that the company have experienced "some consumer traction connected to the PFAS-free label".[48] *GRST* received distinguished recognition in Logitech Internationals "Future Positive Challenge", a "global call to identify disruptive sustainable solutions in electronics" resulting in a collaboration between the companies.[49]

3.2.7. HP (in written)

In response to a request for information, *HP* noted via e-mail on January 28, 2026 that:[50]

> "
>
> The definition of PFAS that is being used in many of the developing regulations encompasses fluoropolymers that are widely used in electronic products, including those sold by HP. HP is currently working with our product development teams and suppliers to understand where these types of materials are used, and find potential alternatives. For more information on where PFAS may be found in electronics, ChemSec offers a useful guide: https://chemsec.org/check-your-tech-new-report-helps-businesses-find-pfas-in-electronics/.
>
> The use of some PFAS is already restricted in HP products, including PFOS, PFOA, C9-C14 PFCA, PFHpA, and PFHxS. For more details, see HP's General Specification for the Environment, Substance and Material Requirements at HP GSE [https://h20195.www2.hp.com/v2/GetDocument.aspx?docname=c05998906]
>
> Fluorinated organics are used in the cathodes of Li-ion batteries. Specifically, PVDF (polyvinylidene difluoride) is used in this application. This material has superior thermal and electrochemical stability, strong adhesion to the electrode, and strong dielectric properties. It is one of the materials that provides the high reliability associated with Li-ion batteries today and is key to making them safe enough for general use. It is the inherent strength of the carbon-fluorine bond that makes this chemical ideal for this application and why it is very difficult to substitute non-fluorinated alternatives.
>
> "

3.2.8. IKEA

IKEA was founded in 1943 in Sweden selling furniture.[51] As of 2026, they sell products such as kitchenware, kitchen appliances, bathroom products, sofas, beds, home electronics, among others.[52]

On February 18, 2026, an interview was conducted with representatives from *IKEA* and a e-mail exchange on March 3, 2026, included brief updates. They stated that *IKEA* have mapped the uses of PFASs across their products, including batteries. NiMH and Li-ion batteries are the only battery technologies used in their products, in rechargeable applications such as portable speakers and solar-powered outdoor lighting. Earlier, *IKEA* also had products containing alkaline batteries, which are now no longer used.

In their mapping, *IKEA* identified two typical uses of PFASs across their products, which is the PTFE binder in electrodes for Ni-MH batteries and PVDF for adhesion in the coating of electrodes for Li-ion batteries. Information about PFAS content in electrical and electronic articles including batteries has been collected from *IKEA* suppliers in order to prepare for the reporting according to US TSCA. According to *IKEA*, the NiMH batteries have a very low content of PFASs.

The *IKEA* representatives noted that they work closely with their suppliers when selecting battery technology. An important requirement from *IKEA* is that the batteries will fulfill the safety standards, both for the battery themselves as well as for the applications in which the batteries are included. These standards are IEC/EN 62133-1 and IEC/EN 62133-2.

Performance criteria are also considered, depending on their application. For example, NiMH batteries, which can be in either AA or AAA format, are sold as stand-alone articles. The Li-ion batteries often have requirements of cycle life, e.g. 8 hours for outdoor lighting.[53]

IKEA further clarified that the company has been working on phasing out PFASs in their supply chain since 2009. For example, in 2016 they phased-out PFASs in textiles, and in 2025 PTFE in non-stick coatings on cookware and bakeware. *IKEA* mentioned that they recognize that intensive work is required to identify remaining uses of PFASs in *IKEA* products and production processes. This information is needed to identify further restrictions and set phase-out plans. According to *IKEA*, a phase-out from all applications will be complex and take time. This is especially the case where technically feasible alternatives are not yet available and alternatives need to be developed and performance of alternative solutions evaluated, as for example in electronic and electrical components.[54]

3.2.9. KURZ

LEONARD KURZ (or in short *KURZ*) work in the field of coatings and has been since the 1930s. Today, they provide roll to roll coating products to a variety of industries such as automotive, consumer, construction, among others. They also provide coating products in the battery space, such as high-performance coated separator films.[55]

An interview was conducted with a representative from *KURZ* on February 13 in 2026. The representative stated that separators consist of a base film which could be PE or PP, but PP is likely only used for low-cost large-scale storage where safety is not a critical aspect. Therefore, coating is not used for PP, but for the better-performing PE. The representative claimed that in early Li-ion battery development, PVDF was used as the binder for ceramic coating, because it was known to be stable in this electrochemical environment. However, polyacrylate-based binders were equally performing, but cheaper (PVDF from 3 to 8 times expensive) and it is likely that the separators produced today (and since about 10 years) are not made with PVDF. Today, most battery types use the same separator technology, according to *KURZ* representative.

KURZ provides two types of products for batteries: separator films and carbon-coated current collector films for Li-ion batteries, Na-ion batteries, and other types, according to the representative. *KURZ* separators (wet-type PE films) are coated with ceramic particles using a polyacrylate-based binder. None of *KURZ*'s products involve PFASs in the ceramic-coated separators.

While coatings of particles to the separators are less common, coatings of a separator to the cathode, so called adhesive coating, are often adopted. This is offered by *KURZ* in their joint venture together with Chinese-based *HORIZON*. According to the representative, this type of separator coating is needed because PE has a low thermal stability and would shrink at temperatures above 100°C. While batteries seldom reach operational temperatures above 60°C, heat temperature testing might be required, particularly in automotive testing with testing temperatures of more than 105°C. If only PE was used, heat shrinkage could be up to 60%.

With PE used as the base film in wet-type separators, the *HORIZON-KURZ* joint venture provides many coating types tailored towards customer's requirements. One example provided in the interview was the use of PVDF applied with spray-, roller- or dot-coating techniques of a 1-3 µm thickness used for pouch and prismatic cells. Another example was the use of a co-coating between ceramic and PMMA materials with a 2-5 µm thickness. The latter was claimed to be completely PFAS-free and only require one coating step. The anti-heat shrinkage function is obtained through the ceramic particles and the PMMA provides the adhesion of ceramic particles to the base film. The PFAS-free type is also possible to obtain with all coating techniques. When *HORIZON-KURZ* compared PMMA to PVDF, with the same a coating technology, the results showed that PMMA had better adhesion compared to PVDF (assessed for peel strength). The ceramic/PMMA product is used in China for two years where its development was initiated by European customers requesting PFAS-free separators. *KURZ* also has ongoing discussions regarding their PFAS-free separators with customers in Europe.

Overall, the adhesive coating depends on process parameters, according to the representative. It can be applied in both lamination and Z-folding production lines, where Z-folding requires adhesive coating. In addition, the battery format would also influence the need for adhesive coating. For example, in cylindrical cells, the electrodes are tightly wound around the separator providing mechanical strength and reduces the need of adhesive coating. However, for long cycle life, even cylindrical batteries would include adhesive coating.[56]

3.2.10. Leclanché

Leclanché was founded in 1909 in Switzerland, producing so-called dry cells (well known as a Leclanché cell). Over their 115-year history, they have produced different cell technologies, such as cadmium-nickel batteries (1935), lead acid batteries (1950) and mercury button cells (1964), among others. Starting 2006, they focused on Li-ion batteries, with a full production of lithium titanate cells in 2012 followed by a ramp up of graphite/NMC111 production in the around 2015. They provide full battery solutions, exemplified with their Li-ion battery module for transport applications which have been implemented in the "world's largest electric ferry" between two islands in Southern Denmark since 2019.[57]

An interview was conducted on December 8 in 2025 with a representative from *Leclanché*. They noted that their Li-ion batteries today are based on the NMC622 chemistry and high Ni-content NMC (and NMC with low Al content, so-called NMCA) with graphite anode. They stated that Leclanché have removed the need for the NMP solvent for more than 13 years, already switching then from PVDF binder dissolved in NMP to water-based binder electrode manufacturing. The representative stated that the goal was to switch to a water-based binder solution. At that time, however, Leclanché were not focusing on PFAS-free technologies but rather looking for more environmentally-friendly water-based binder solutions to avoid the hazardous and harmful effects of using NMP as a solvent with PVDF binder. Furthermore, safety regarding one parameter was improved, since no HF can be generated in these systems through binder decomposition, as the binder used does not contain fluorine.

Instead *Leclanché* uses a water-based binder that is based on a mixture of water-based binders and surfactants.[58] The representative also presented their patent, which state that the binder "contains a combination of carboxymethyl cellulose (CMC) and at least another binder compound chosen from the group comprising, polyacrylic acid (PAA), styrene-butadiene rubber (SBR), modified styrene-butadiene rubber (SBR), acrylate-based rubber (ABR), and other acrylate binders." In addition, it is noted that "the binder may comprise pH and/or rheology-control additives such as polyethylene-co-acrylic acid (PEAA), phosphoric acid (H3PO4), citric acid (C6H8O7), formic acid (HCOOH), boric acid (H3BO3), Lithium dihydrogen sulfate (LiH2SO4), Lithium dihydrogen phosphate (LiH2PO4), or ammonia" with some additional details of the combination of these.[59]

According to the *Leclanché* representative, their battery (NMC622 chemistry with graphite anode) can work for more than 5 000 cycles with operational voltages up to 4.35 V. The battery has been on the market since 2019 and the products that were placed on the market then are still in use, indicating high stability. Leclanché's batteries are also used in the marine and energy storage.

Leclanché noted that they are not yet PFAS-free but use a small amount (<1%) PFAS in their batteries because their production consists of a lamination step of the separator to the cathode, in which they use PVDF. This lamination material for the PE separator improves adhesion that is required for lamination which results in a long cell cycle life. However, the representative noted that there are no technical barriers to remove the PVDF from this process, as another technique (Z-folding) would be possible to manufacture cells using stacking technology without lamination. Leclanché's limitation is that changing process requires investment of another production line or use of a subcontractor who could provide this today. The representative noted that the Z-folding process is already used by many companies today. In

comparison to the lamination of separator to the electrodes, which the representative notes would be possible substitute, they note that ceramic coating of separator is necessary for larger format cells to enhance the cell safety.

Leclanché's solution is now patented and has been granted project on an EU-level to set up a 2 GW factory, moving from TRL 6/7 to TRL 9. Leclanché states that their challenge is not linked to the performance of the battery, but in scaling up production.[58]

3.2.11. Marshall

Marshall is a company producing music equipment and consumer electronics with products such as amplifiers, headphones, speakers and drums.[60] In 2026, they had around 800 employees at 9 locations.[61]

On January 28, 2026, an interview was conducted with a representative from *Marshall*. They noted that, as a downstream user, *Marshall* have tried to obtain PFAS-free batteries for several years in collaboration with their supplier based in China. While *Marshall* earlier had been informed that it was possible to find PFAS-free solutions for small-scale products (coin cell batteries), recently (autumn 2025) they were notified that these products were no longer available by their suppliers (with the exception for one type that was not relevant for *Marshall*). *Marshall* stated that one reason for this recent development could be linked to regulatory uncertainty. As such, the representative argued that there are no technical barriers to PFAS-free coin cell batteries. Instead, the availability is dependent on the demand from the downstream users, particularly those that can procure in large quantities.

In large, cylindrical batteries, *Marshall* representative noted that PFASs are used in the electrolyte and separator. They have not been able to find any PFAS-free cylindrical cells. Still, *Marshall* stated that they are working to connect their suppliers with *Westra Materials,* which also may reduce the need for NMP or activated carbon.

Apart from technical requirements, the parameters of cost and timeline were discussed by *Marshall*. In terms of the former, they mentioned that it is not a given that the material itself is costly, but that the cost per customer would possibly be higher if only few are sharing costs for certification and testing. Regarding the latter, the time to have a market-ready PFAS-free product was estimated by *Marshall's* representative to be about 3-4 years, including evaluation, testing (cycling) and certification. *Marshall* notes however that if there is a time-indication for when PFASs in batteries would be restricted, then this would already push for companies initiating the phase-out.[62]

3.2.12. RECHARGE

An interview was conducted on February 2, 2026, in which representatives from *RECHARGE* were present.[63] On February 27, 2026, minor clarifications were received via e-mail.[64]

In general, the representatives noted that their submission to the Annex XV public consultation contained the most detailed information, which had been gathered from their members (#8680). Overall, they noted the uses of PFASs in batteries involve the binder (e.g. PVDF in Li-ion and Na-ion batteries, and solid-state batteries), electrolyte (e.g. electrolyte additives in Li-ion batteries but also solvents and co-solvents) and sealing applications including gaskets, valves, washers, O-rings, among others. Since *RECHARGE's* Annex XV submission, *RECHARGE* stated that they would like to emphasize two additional uses: 1. Electrode edge coatings and 2. Electrical insulation components in cells and batteries.

RECHARGE representatives provided a high level, general overview of key performance indicators as they are typically considered from an OEM perspective. Many of these indicators can conflict with one another in practice, and OEMs often face the challenge of finding the most appropriate compromises depending on the specific application. *RECHARGE* stated that these include (weight in parenthesis as explained by *RECHARGE*, out of 5):

- Safety (5)
- Energy density (4)
- Charge time (3)
- Cost (5)
- Lifetime (3)
- Sustainability (3)

They argued that there is a “fundamental tradeoff” between technical requirements and that these must be “customized application per application”, where a best compromise is different between different end-uses. Three examples, which referred to a report from Volta Foundation,[65] mentioned included:

- Safety and lifetime, in which PFASs is used in chemically aggressive environments, as leakage would decrease the performance within these indicators.
- The energy (density), where NMC (and also NCA) cells have the highest packing efficiency, which can be used in EVs, and while LFP are not as energy dense, it provides benefits in terms of cost, lifetime and safety. It was noted that an 8.3 kWh pack could be of different sizes:
    - LFP (81 kg, providing 125 Wh/kg),
    - NMC (41 kg, 200 Wh/kg) and
    - NMC (43 kg, 230 Wh/kg).
- The form factor, where
    - Pouch cells provide both high volumetric energy density (energy per volume) and gravimetric energy density (energy per weight), however, it is fragile and therefore lower safety, and requires thermal and mechanical management on a pack level.
    - Prismatic cells provide high volumetric energy (due to excellent packing efficiency) but medium gravimetric energy and it is also susceptible to swelling and has thermal non-uniformity. It could also be expensive.
    - Cylindrical cells provide both low volumetric and gravimetric energy density due to low packing efficiency and heavy casing; however, it provides thermal uniformity, mechanical robustness, fast winding speed during manufacturing and better safety than pouch. It has the lowest cost per energy unit.

RECHARGE explained that evolving regulation and rising customer expectations are increasing the pressure to improve a wide range of technical requirements simultaneously, even when these requirements can conflict with one another. Representatives emphasised that battery cells developed for automotive applications already operate extremely close to the boundaries of what current materials, manufacturing processes, and engineering methods can reliably deliver. That is, according to *RECHARGE*, designing battery cells for electric vehicles differs fundamentally from designing cells for consumer electronics or stationary storage. Automakers expect high performance across many dimensions at once, and those expectations often pull in opposing directions. As a result, automotive cell development consistently operates at the limits of what current technology can deliver. They noted that solutions demonstrated at laboratory or pilot scale often look promising, but can differ significantly from what is feasible in large-scale, market-ready production, where safety, durability, and cost constraints are far more stringent.

RECHARGE stated that they must comply with EU competition law and work through antitrust[c]. Therefore, they are not permitted to discuss company-specific alternatives being

used or developed by members However, *RECHARGE* was open to discuss alternatives at a technical level. Here, the representatives noted that literature, conference papers and products from companies show that cathode binders based on acrylic acid have shown promising results, although these require optimization in terms of performance also at lower volume percentage. In terms of electrolyte, they noted that a large majority of Li-ion batteries rely on $LiPF_6$ (which is not a PFAS) as a lithium electrolyte salt however, some batteries require PFAS-based salt additives (such as LiTFSI) for lithium-metal-polymer (LMP) solid-state batteries as well as high voltage batteries.

Overall, the timeline for a new development to be market ready was referred to Figure 9 in their Annex XV consultation comment (#8680), which reports the different stages for alternatives development. *RECHARGE* states that industry experience demonstrates that 8 years or more would be required from the identification of a new alternative, through the development and combination of other components and integration into product designs and manufacturing processes.

Lastly, the interview included a few clarifying questions to the (#8680)submission:

- Page 12, Table 1: the positive electrode for Ni-based rechargeable batteries is applicable to both the NiCd and NiMH battery
- Page 12, Table 1: a typo noted for the primary Li-SOCl2 and primary Li-SO2 positive electrode, should be "PTFE with carbon black".
- Pages 21-22: PFAS are not only used in solid-state batteries with LMP chemistry but also for e.g., lithium sulfur batteries.
- Page 23, Table 2: Metal air batteries also include Zinc air batteries
- Pages 12-14 and page 26: PVDF provide the same functions in Na-ion batteries as in Li-ion batteries.

During the interview, two questions were raised for which *RECHARGE* requested additional time to answer. The written questions and responses were as follows:

1. Which battery technologies are the 'lowest hanging fruit' to make fully or partially (i.e. one component) PFAS-free? If so, what applications are these used in?

"RECHARGE members are aware of researchers looking at alternatives but this is not lowest hanging fruit as it is not already commercial. There may be possibilities for alternatives for low voltage type batteries in the coming years however, this does not look possible for high voltage. We recommend reading the FIPRA report which provides a good discussion on this topic."

2. What are the functions provided by PFASs in electrolytes?

"The main function of PFAS in electrolytes is to maintain stability at high voltage. There is an emerging research area which looks at localised high concentrated electrolytes (LHCE) using fluorinated ethers (a type of PFAS) which provides better stability of interfaces inside the cells."[64]

3.2.13. Westra Materials

On March 18, 2026, an interview was conducted with a representative from *Westra Materials*. The representative noted that PFASs are found in electrolyte and cathode binder on the battery

---

[c] Addition from authors: The EU competition Law involves restrictions on sharing commercially sensitive information between competing parties, either directly or indirectly, and market coordination practices (pricing, behavior, etc.) are prohibited.

cell level. In terms of the former, which is a mixture of salts, it would be possible to find non-fluorinated alternatives and accommodate the formulation depending on the chosen cell chemistry. In the anode, the representative noted that on the market, CMC/SBR is the gold standard.

However, the *Westra Materials* representative claimed that it is more difficult to substitute PFASs in the cathode, for which PVDF and PTFE could be applied as a binder. Instead, PVDF is being the benchmark binder material for current Li-ion batteries. Although many alternatives are being investigated on lab scale, the representative argued that it does not guarantee successful transfer towards industrial applications. PTFE on the other hand is preferred for dry battery processing, an up-and-coming technique to achieve higher energy density Li-ion batteries and solid-state batteries. Within dry processing, it would be difficult to find a substitute.

According to the representative, *Westra Materials* offer a binder which is based on a conductive polymer and is successfully validated in terms of processing and performance for the current state-of-the-art battery manufacturing infrastructure. The representative reported that most often, the binder should possess properties such as dispersing, binding, adhesion and coating ability to be suitable for the cathode. By providing a conductive binder, Westra can offer a solution that would enable substituting two components, both the binder and the conductive additive. This would directly decrease the inert/inactive material of the battery and thus increase the energy density.[66]

3.2.14. Anonymous responses

A few other interviews were also conducted but requested to remain anonymous. To keep confidentiality, the results are aggregated in a short summary, as follows.

3.2.14.1. Alternatives

In addition to being present in the cell, polymers could also be used in batteries as a casing. In certain cases, e.g. pouch cells, it is a requirement to be flexible. It is possible that the polymers (non-PFAS) for casing could be applied as binder inside as well, fulfilling similar functions. For example, one respondent noted that a functional polymer with high content of maleic anhydride could perform “quite well in particular harsh conditions” and it is possible to adjust the proportions to provide reactivity, adhesion and chemical bonding between different substrates.

3.2.14.2. Timelines

Several representatives of actors along the battery value chain note that battery manufacturers would not change their production line unless there is a regulatory push[d], because, as one representative noted, it would require some downtime on the production line.

3.2.14.3. Other

In written, one respondent highlighted the report “Information exchange on PFAS in lithium-ion batteries“ organized by FIPRA, with the participation of ZSW and RECHARGE, published October 2024.[67] The report “discuss the complex lithium-ion battery chemistries which are used to achieve very different performance requirements in the automotive, power tools and consumer electronics sectors.”

An example includes the PVDF binder in Li-ion battery cathodes (specifically noting the LCO, NMC, NCA, LMNO, LMO, and LFP chemistries). In a figure, the report lists the functions

[d] “Solution has to be driven from policy”; “A [regulatory] indication is enough [for downstream users to require PFAS-free solutions]”; “Finite dates means targeted work”; “Regulation necessary for sustainability”;

and properties linked to this use, which are identical to those listed RECHARGE's consultation comment (#8680), with the addition that the polymeric binder also "withstands active material expansion/contraction and high/low voltages" and "withstands high temperatures". The reports states that "[t]oday there are no commercially available alternatives which can meet all these functional requirements in all applications."

The range of applications of Li-ion batteries is noted to be large. Here, the report state that "cell vendors use PFAS cathode binder materials to manufacture all battery cell chemistries to meet these very different performance requirements in different applications" including technical requirements and key performance indicators for three examples:

- Automotive
  - Capacity of 50 – 250 Ah,
  - Cycle life of 2 000 – 10 000, and
  - Customer demands in safety, fast charge time, among others.
- power tools
  - Capacity of 2 – 10 Ah,
  - Cycle life of 500 – 2 500), and
  - Customer demands of high power and compatibility (standardized cell dimensions), among others.
- consumer electronics
  - Capacity of 0.3 Ah – 10 Ah,
  - Cycle life of ~1 000), and
  - Customer demands of high volumetric energy (i.e. high cell charge voltage and capacity) and high charge/discharge rate, among others.

The report claims that PVDF binder is the only option based on performance for these applications but note that "some companies have adopted non-PFAS polymers as cathode binder in some battery applications". They also include Figure 9 from RECHARGE (#8680) to describe the timeline.

3.3. Detection of PFAS by teardown-analysis studies

Teardown-analysis studies were identified via database searches. Although this research is mainly focused on the electrochemically active material and/or the design of the cells, some information was related to the uses of PFASs in batteries, mostly related to the binder. An overview of these results can be found in Table S12.

Table S12. Results from teardown-analysis studies of commercial batteries. *As specified by supplier, not measured in study. List of abbreviations: Lithium iron phosphate (LFP), Lithium cobalt oxide (LCO), Lithium nickel manganese cobalt (NMC), Lithium nickel manganese cobalt aluminum oxide (NMCA), styrene-butadiene rubber (SBR), carboxymethyl cellulose (CMC), polyvinylidene fluoride (PVDF), polytetrafluoroethylene (PTFE), polyacrylic acid (PAA), polyethylene oxide (PEO), scanning electron microscopy (SEM), thermogravimetric analysis (TGA), energy-dispersive X-ray spectroscopy (EDS, EDX), inductively coupled plasma optical emission spectroscopy (ICP-OES), X-ray diffraction (XRD), differential thermal analysis (DTA), X-ray photoelectron spectroscopy (XPS), and Fourier transform infrared spectroscopy (FTIR).

**Company Model Format** (Configuration)	**Cathode**			**Anode**			**Other components, e.g., electrolyte and separator**	**Reference**
	**Chemistry**	**Binder**	**Method for material characterization**	**Chemistry**	**Binder**	**Method for material characterization**		
Tesla Motors **Model 3 Prismatic (003, E6R)**	LFP, carbon-coated	n/a	Not tested	Graphite, no Si	No conclusion	EDX: “90.8% carbon and 7.2% oxide were detected, while all other elements – including fluorine residues from the solid-electrolyte – were below 1%.”	ICP-OES: “conducted at E-Lyte revealed that only $LiPF_6$ was used as a conducting salt, as no traces of sulfide or bromine were detected (see Table A.1 in the appendix), which could hint towards another conducting salt like lithium bis(fluorosulfonyl)imide (LiFSI), lithium bis(trifluoromethane-sulfonyl) imide (LiTFSI), or lithium tetrafluoroborate ($LiBF_4$).”	Stock et al. (2023) [68]
BYD **Cylindrical (4680)**	LFP, Ti-doped	n/a (slurry-based method)	Not quantified<br>SEM: “No fiber-like morphology was seen for either the cathode or anode, suggesting the conventional slurry-based processing of the electrodes, in contrast to next-generation solvent-free electrode processing techniques.”	Graphite, no Si	CMC (slurry-based method)	EDS: "A sodium atomic ratio of 1.8% suggest the possible use of sodium carboxymethyl cellulose (CMC) as a binder"		Liu et al. (2025) [69]

***BYD* Blade Prismatic**	LFP	PVDF	TGA: "a slight mass loss occurs ... between 400° C and 500 °C. The loss can be attributed to the thermal decomposition of the polyvinylidene fluoride or polyvinylidene difluoride (PVdF) binder at this temperature range."	Carbon, no Si	CMC and SBR (water-processing)	TGA: "presents two peaks, which can be identified with the decomposition of carboxymethyl cellulose binder (CMC) between 200°C and 300°C and the decomposition of styrene butadiene rubber (SBR) at temperatures between 300 C and 450 C."		Gorsch et al. (2025) [70]
***Tesla Motors* Model Y Cylindrical (4680)**	NMC811	CMC and SBR	TGA: "a slight mass decrease is observed between 200 C and 300 C and a steeper one between 300 C and 450 C. These two may correspond to the decomposition reactions of CMC and SBR. This would suggest that the Tesla cathode has followed an alternative fabrication process that differs from typical PVdF-Nmethyl-2-pyrrolidone-based cathode processing"	Carbon, no Si	PAA and PEO	TGA: "different binders can be distinguished. The first mass decrease, between 300 °C and 350 °C, may correspond to the chain-splitting reaction of polyacrylic acid (PAA) polymer chains. In comparison, the weight loss around 450 °C would fit the degradation of PAA polymer chains into monomers. The more substantial peak at about 380 °C is consistent with the degradation of polyethylene oxide (PEO)."		
	NMC811	PVDF	EDX: "The EDX analysis of fluorine showed accumulations between the active material particles and a total amount of 7.9 %. This led to the conclusion that polyvinylidene fluoride (PVdF) was used as a binder as it is one of the most commonly used binders in cathodes."	Graphite, no Si	PVDF or PTFE (solvent-free)	EDX: Furthermore, fluorine is present at a significant quantity of 7.9 %, which may suggest the presence of a binder containing fluorine, such as PVdF as found in the cathode, or polytetrafluoro-ethylene (PTFE). The utilization of PTFE could indicate a solvent-free anode fabrication method." "Although copper, as a heavy metal element used in the current collector foil, is evident throughout the coating	"Small traces (<0.5 % each) of phosphorus and sulfur were also detected by the EDX which are residues of lithium salts used in the electrolyte. Examples for commonly used lithium salts containing phosphorus and sulfur are lithium hexafluorophosphate (LiPF6) and lithium bis(fluorosulfonyl)imide (LiTFSI).; "Similarly to the cathode, trace amounts of	Ank et al. (2023) [71]

						and appears in the EDX measurements, its quantity is minimal, constituting less than 1 %. Between the coating and the copper collector, a black primer coating was observable. It exhibited a notably distinct structure from the actual anode coating and extended approximately 1.5 mm beneath the coating. EDX analysis revealed that it primarily comprises carbon and fluorine, leading to the inference that the same binder as in the coating, along with carbon black for electrical conductivity, was used. This primer coating strongly suggests the utilization of a solvent-free coating process.”	phosphorous and sulfur are detected (<0.5% each), supporting the hypothesis that they originate from lithium salts in the electrolyte.”	
***Volkswagen* ID.3 Pouch**	Between NMC622 and NMC811	n/a	Not tested	Graphite, no Si	No conclusion	EDX: F (5.7 w%)		Günther and Wass-iliadis (2022) [72]
***Samsung* 50G Cylindrical (21700)**	NCA	n/a	Not tested	Graphite with Si	No conclusion	EDX: F (2.83 +- 0.03) mass percent		Ank et al. (2025) [73]
***LG* M50G, M50T, M50LT Cylindrical (21700)**	NMC811	unknown	EDS: “Besides small quantities of fluorine and phosphor below 0.1 mol-% no other elements were detected. These quantities are assigned to the cathode electrolyte interphase (CEI)”	Graphite, no Si (M50LT) Graphite, with Si (M50T)	PVDF	XRD: “The peak at 2Θ ≈ 24° can be referred to the binder (e.g. PVDF).”; EDS: “Furthermore, phosphor and fluorine were detected in the electrode which can be interpreted as part of the SEI and are therefore also not displayed in the EDS images.”		Stapf et al. (2025) [74]

***LG* M50 Cylindrical (27100)**	NMCA	n/a	Not tested	Graphite, with Si	Water-based	"The negative electrode coating was delaminated easily using water, which indicates a presence of a water based binder, such as CMC-SBR"	SEM: "The separator was ascertained to be a ceramic coated polymer with a thickness ca.12 µm, this approximation was due to a non-uniform thickness at different parts of the separator"; "The density of the separator corroborated that it was ceramic-coated rather than a normal polyolefin (CnH2n) membrane."	Chen et al. (2020) [75]
**Unknown, commercial pouch**	NMC811	Not specified	TGA: "TGA the cathode material initiates gradual decomposition and exothermic reactions upon reaching around 200 °C. At around 800 °C, approximately 9 mass% of the material underwent physical and/or chemical reactions, leaving about 91 mass% remains unreacted. This is attributed to the thermal decomposition and evaporation of residual binders, conductive agents, and electrolyte mixtures during the initial stage of cathode powder treatment. In contrast, the bulk of the cathode material remains undecomposed."	Graphite	Water-soluble	TGA: "The anode material demonstrates abrupt decomposition and exothermic behavior commencing at around 500 °C and persisting until around 700 °C."; "After a drying period of 12 h, the anode was immersed in deionized water to dissociate the anode material from the copper foil."	"The separator material undergoes rapid thermal decomposition and heat release commencing at around 210 °C and persisting up to around 500 °C. The reaction culminates at a peak temperature of 430 °C. It is inferred that the residue might be ceramic. This thermal behavior is likely attributable to the oxidative degradation of polymeric components, such as polypropylene (PP) and polyethene (PE), upon heating, yielding oxidation by-products."	Guo et al. (2025) [76]

**Unknown commercial Pouch (stacked)**	LCO	PVDF	EDX, “Findings revealed that cathode ... were combination[s] of active cathode material (lithium cobalt oxide; O, Co elements), binder (F element, PVDF), and carbon black (C element).”	Graphite	unknown, F content attributed to SEI	EDX F=5.26 wt; “In addition to the major elements, small amounts (<10%) of oxygen, sulfur, fluorine, phosphorus elements were also detected. The corresponding signals could be generated from the electrode binder, and residual electrolyte attached to the electrode”	FTIR, "the material for ... separator[s] was PE based on the characteristic absorbance bands. The manufacturer specification does not reveal the separator material, identification of separator material was expected by the device manufacturer.”	Kong et al. (2022) [77]
**Unknown commercial Pouch (jelly-rolled)**	LCO/ NMC*	PVDF	EDX, “Findings revealed that cathode ... were combination[s] of active cathode material (lithium cobalt oxide; O, Co elements), binder (F element, PVDF), and carbon black (C element).”	Graphite	unknown, F content attributed to SEI	EDX F=5.64 wt%; “In addition to the major elements, small amounts (<10%) of oxygen, sulfur, fluorine, phosphorus elements were also detected. The corresponding signals could be generated from the electrode binder, and residual electrolyte attached to the electrode”	FTIR, "the material for ... separator[s] was PE based on the characteristic absorbance bands. The manufacturer specification does not reveal the separator material, identification of separator material was expected by the device manufacturer.”	
**Unknown commercial Pouch**	LCO*	n/a	Not tested	Graphite*	n/a	Not tested	FE-SEM/EDS. “The surface of the normal anode (graphite) electrode is mostly composed of P, F and O, and these materials likely originated from side reactions of electrolytes such as LiPF6”	Lee and Ko (2018) [78]
**Unknown, commercial pouch**	NMC111	n/a	EDX: Peak of F, unknown intensity (cathode)	Graphite	No conclusion	EDX: Peak of F, unknown intensity (anode)	EDX: Peak of F, unknown intensity (separator)	Hamed et al. (2024) [79]

From these results, PVDF is identified as the binder in several cases, such as by Gorsch et al. (2025) in the cathode of *BYD* Blade prismatic cell of LFP chemistry.[70] However, some results are contradictory. Although Ank et al. (2023) measure F in the cathode to 7.9 wt% and links this to the use of PVDF,[71] Gorsch et al. (2025) reports TGA peaks at a different temperature than expected for PVDF.[70]In most cases, there is no mention of cathode binder identity. However, for the anode, most works provide information about the binder, either by providing a suggesting the identity (CMC and possibly SBR) in *BYD* Blade,[69,70] or through the weight percentage of F through EDX/S measurements.[72,73] In some cases, the F content is around 5-8%, which Ank et al. (2023) links to the use of PVDF or PTFE.[71] In other cases, the percent is cited to be lower, often connected to the use of F in electrolyte, which degrades into a solid-electrolyte interface on the anode.

3.4. Selected case studies: Survey of the academic literature

3.4.1. Case 1: Li-ion/LFP chemistry/Cathode (potential PFAS use no. 6; technical function: "binder")

In the literature review, a total of 25 studies were eligible and fully examined for insight into the use of cathode binders in Li-ion batteries of LFP chemistry. Although five reviews were not relevant for the research question formulated in this work and therefore not mentioned further, many gave critical insights into the technical requirements for a cathode binder in Li-ion batteries of LFP chemistry.

3.4.1.1. LFP chemistry: A case of battery development optimization

The reviews provided some background information of Li-ion batteries of LFP chemistry, in which the cathode is made of a lithium iron phosphate (LiFePO4) material. This battery chemistry was first developed in 1997 and has a theoretical capacity of 170 mAh/g and voltage around 3.5 V.[80–82] It was considered a promising material at the time, with advantages such as cycling and thermal stability, increasing safety[81–85] as well as elemental abundance, low toxicity and low cost.[81–84]

On the other hand, LFP has significant drawbacks, including poor electronic conductivity slow Li-ion diffusion and relatively low operating voltage.[81,83,84] Therefore, it have not been the preference by all battery developers. Instead, the cathode material made of lithium nickel manganese cobalt oxide (NMC), with a theoretical capacity around 160 – 220 mAh/g and voltage around 3.6 V (depending on the relative proportions of the three transition metals), gained wide interest in state-of-the-art research and development in academia and among for example electric vehicle OEMs.[82,86]

Overall, apart from the capacity and voltage, which determine the energy density, many parameters are important for battery producers and users, such as low cost, long cycle life (i.e. the number times it is able to recharge), (dis)charge capacity (speed at which a battery can be charged or provide energy), safety (risk of thermal runaway and, in extent, fire), among others. As a result, in most recent years, the use of LFP in electric vehicles has increased yet again, linked to its safety reputation and low-cost production in connection to recent engineering developments to improve energy density.[81–83,85,87,88]

3.4.1.2. Commercial use of PFAS-based cathode binders in Li-ion battery of LFP chemistry

Most commonly, a Li-ion battery cathode is produced in a so-called wet process, in which a slurry containing a solvent and the active material (e.g., LFP) and a conductive additive coated on the current collector. The slurry also contains a binder which provides structural stability of the final electrode.[81,83,84] The binder makes up about 2-5% of commercial cells.[80] An important factor is the rheology of the slurry, which affects the quality of the final product.[89] After the coating, the electrodes are dried and pressed (with so-called calendaring).[81,90]

A total of eight literature reviews reported the use of PVDF as being the most common in Li-ion batteries[80,82–85,89,91] of which three reported this particularly for commercial use,[80,83,89] and three for commercial LFP chemistry specifically[80,83,84] As PVDF only can be dissolved by a few solvents, NMP was reported to be very common commercial production and the conductive agent was often carbon black (about 1-5 %).[88,90,92,93]

Researchers have also investigated if co-polymers of PVDF could yield good results, including PVDF-HFP and PVDF-TrFE[84] as well as co-polymers with more than two monomers.[80,83]

The included works stated that PVDF has beneficial properties for its use as a binder, including high mechanical strength, good wear resistance, excellent weather resistance, good electrochemical stability, relevant electrolyte absorption, good wettability, thermal stability, chemical stability, among others.[80,84,85,89] The electrochemical stability is exemplified by Lai et al. (2026), which report that it has a so-called broad electrochemical window between 0 and 5 V, in which it is resistant to oxidation by the cathode.[85] In the cathode, PVDF molecules bind the active materials among themselves and the active materials to the current collector while ensuring the uniformity,safety of active materials and facilitating the solid electrolyte interface formation on the graphite surface. As a result, the Li-ion battery would possess an improved cycle performance, fast charge and discharge, and reduced internal resistance.[80,85]

However, these characteristics are dependent on the conditions of the cell. At certain stresses, such as long cycling, higher mass loading, high voltages and high temperatures PVDF loses adhesion capability due to weak van der Waals forces and low chemical stability, resulting in loss of performance.[82,84,85,89,92] At temperatures of 160°C, PVDF melts and at 350°C it decomposes, risking thermal runaway which affect the battery safety.[85] In addition, Choi et al. (2025) report that "the weak mechanical strength of PVDF leads to electrode delamination and volume changes, indicating the need for the development of new binders for high-performance LFP cathodes"[83] and according to Zhang et al. (2023), this could be due to swelling of electrolyte at elevated temperatures.[89] Furthermore, Hwang et al. (2024) report that the absorption of electrolyte by PVDF "weakens the adhesive strength and mechanical strength of the binder and may cause potential instability in the integrity of the electrode."[92]

In addition, PVDF was reported to have other disadvantages, such as being electronically insulating, resulting in the need of adding conductive additives into the active material matrix.[85,89,94] The binder and conductive additive is considered "dead weight" which overall reduce the energy density of a battery,.[81,95] particularly for LFP with one-dimensional channels for Li-ion insertion when undergoing redox reactions.[84,85]

Another disadvantages include using NMP which produce HF gas and toxic in itself.[80,83–85]. In addition, in the cathode preparation, the solvent must be recovered which consumes energy. In comparison to water, the process with NMP is twice as costly.[85] Furthermore, recycling experience similar issues when solvent-dissolution stage is implemented with NMP.[84,85]

The disadvantages with wet manufacturing of LFP cathodes have pushed for development into dry processes (also known as solvent-free), in which PTFE is the most common.[80,85,90] The PTFE provides a binding function by forming a net-like structure of PTFE fibres around the active material "forming cotton candy-like powders".[90] As a result of the low surface energy of PTFE and its hydrophobic properties, it can obtain a high contact angle with the electrolyte leading to reduced interactions between the electrode and liquid electrolyte (i.e., low wettability).[90] On the other hand, research has been conducted on water-borne PTFE binder in LFP to replace the NMP-dissolved PVDF.[84]

3.4.1.3. *Potential alternatives to PFAS use in Li-ion battery of LFP chemistry based on academic research*

As explained above, the limited solubility of PVDF in non-hazardous solvents has caused issues during e.g., recycling, initiating research into binders that would be soluble in non-toxic

solvents, preferably water.[82,85,95–98] When manufactured, these water-soluble cathode binders provide advantages such as low cost, less environmental concern (particularly from the solvent) and faster drying speed, among others.[82,85,97] This is already enabled in the anode fabrication.[97] Non-PFAS examples of cathode binders in Li-ion battery with LFP chemistry include carboxy-methylcellulose CMC, used in polymer blends, possibly with styrene-butadiene rubber (SBR) as well as polyacrylic acid (PAA) and poly(vinyl alcohol) PVA.[80,85,96] Many water-soluble polymers contain polar functional groups, which allow for not only physical adhesion but also chemical bonding.[84,85,89]

Water-soluble binders, often composed of organic polymers have reduced thermal stability at temperatures above 400°C.[85] Therefore, other techniques have gained interest, such as conductive binders or binders with multifunctionality (e.g., self-healing, resistant-reducing or change in conductivity depending on voltage).[83,89,94] While coating of LFP electroactive material results in the material to both be conductive and electroactive already today,[88,95,99] advanced materials could include a coating with an added benefit of binding function.[80,81,92,94] In addition, so-called binder-free technology, in which the cathodes are thin enough to keep the electrochemical performance have been suggested.[99] These use nanostructures or nanocomposites but require modification, and are only available for certain lightweight devices.[99,100]

3.4.1.4. Notes on the commercial use of cathode binders in Li-ion batteries of other chemistries

As discussed above, water-based binders are considered potential alternatives to PVDF and PTFE binders in LFP cathodes. This is because LFP has a low sensitivity towards water, being compatible with water-based processing[85,97,101] which is due to the strong Fe-P-O bonding.[101]

In the identified works, which were obtained because they review the use of cathode binders in LFP cathodes specifically, information about those for other chemistries was also included. For example, compared to LFP, other cathode materials, such as transition metal oxide cathodes (e.g., NMC, NCA etc.) are more sensitive to water as this could result in dissolution of Li-ions and transition metal ions from the surface, eventually leading to corrosion of the current collector (made of aluminium) and reaction with water resulting in reduced energy, cycle and safety performance.[85,97,101]. Still, some water-soluble binders for NMC have been reported in the literature. Lai et al. (2026) report that a mechanism to protect the transition metals would be needed and describe the use of polyionic liquids for NMC811 to stabilize the cathode structure.[85] In addition, Park et al. (2025) reported that both LFP and “some NCM-based” cathodes are manufactured with water-soluble binders, although scaling was considered a challenge.[90]

As with LFP, the most commonly referred to binder for NMC chemistry in commercial use was PVDF and in research, dry coating with PTFE.[80] Still, both are based on physical adhesion and researchers have investigated other materials, including fluorinated polyimide in which the polymer backbone is polarize with -CF3.[80,89] In addition, Zhang et al. (2023) report that researchers showed that CMC/SA could protect a (lithium nickel manganese oxide (LNMO) cathode at high temperature cycling. At high voltages, the formation of a cathode electrolyte interface (CEI) is required, which can be obtained with selecting e.g., a lignin as the binder. [89]

3.4.1.5. Notes on the commercial use of anode binders in Li-ion battery of LFP chemistry

Although the review articles all discussed cathode binders, some also reported uses of anode binders. Most commonly, CMC and SBR are used as anode binders in Li-ion batteries.[89,91,98] as well as other water-based polymers such as alginates and polyacrylic acids, among others.[91] As water-processing is possible for the anode, these binders are preferred because of their low cost and minimized hazards[91] and increased lifetime compared to PVDF.[82] In short, CMC is

used to obtain a desirable viscosity in the slurry, while SBR functions as an emulsifier complementing each other.[89,91]

3.4.2. Case 2: Li-ion/Liquid electrolyte/Separator, coating (potential PFAS use no. 17)

A total of 23 literature reviews were eligible in the case of separator coatings of Li-ion batteries of which 15 included information relevant to our study.

3.4.2.1. Separator function and requirements

The function of the separator is to prevent physical contact between the anode and cathode while allowing ion transport.[102–108] Because it plays a critical role in preventing internal short circuit and thermal runaway, the separator is a major contributor to overall battery safety and therefore a key component of the cell.[102,104,107,109–111] Although an inactive part of the battery, it also influences the performance over the full lifetime, by e.g., providing storage space for electrolyte and influence ionic transport.[103–105,108,110,112]

In Li-ion batteries, the separator pores should be smaller than the electrode particles, around or less than 1 µm to allow for ionic transport while avoiding direct contact between electrodes[102,103,105,108,110,113,114] and the thickness of the separator should be uniform around 25 µm to balance safety and performance,[103–106,108,113] although these properties depend on the material and battery technology.[103,106] Specific technical requirements are listed e.g., in Zhu et al. (2021) and Costa et al. (2019).[103,114]

The characteristics that are required for a separator are listed by many records including (electro)chemical stability, high ionic conductivity, limited thickness, small pores, uniform permeability, high mechanical strength, high electrolyte affinity and uptake, low thermal shrinkage, shutdown effect, low cost, suppress dendrite growth via high tensile and puncture strength, among others.[102–104,107–114] However, there is no separator that possesses all desired properties, and the component or battery developer must consider trade-offs balanced for the specific battery technology (such as chemistry, configuration etc.).[102,103] For example, a thin separator allows for ionic conductivity but decreases its temperature resistance.[105] Thus, commercially available 10 µm separators increases the battery energy density but increases the risk of failure.[107–109,113] In commercialization, the cost of the battery, its materials and the production, partly related to e.g. scalability and material volume, is one of the main deciding factors in selecting the separator type.[102,103,113,115] Most often, it is a balance between safety and performance (notably the energy density).[108,112]

3.4.2.2. Commercial separator materials

The separator of secondary batteries (e.g., Li-ion batteries, Na-ion batteries) is often composed of a porous membrane that is soaked in electrolyte and today, the polyolefins polyethylene (PE) and/or polypropylene (PP) dominate the market.[102–105,107–109,112–116] A list of marketed polyolefin products can be seen in e.g., Lingappan et al. (2023) and Waqas et al. (2019).[104,112]

A key advantage of PE and PP separators is their low cost, which is related to the manufacturing simplicity and low material cost.[102,103,107,108,113,115] They also possess high porosity, good insulation properties, mechanical strength and (electro)chemical stability.[102,108,112–115] However, they have low thermal stability resulting in compromised safety and low wettability due to its hydrophobic nature.[102,103,107–109,111–113,115,116]

By combining the polymers into a tri-layer structure (PE-PP or PP-PE-PP, e.g., Celgard 2325), thermal shrinkage can be reduced, and a shutdown function can be obtained based on the difference in the polymers' melting points.[102,107–109,112] Although polyethylene (PE) and polypropylene (PP) have relatively low thermal stability, with melting points of about 135 °C and 165 °C respectively, this melting behavior provides an important safety function in Li-ion batteries.[107,111] As the temperature rises, the lower-melting PE layer softens first and closes its pores, creating a clear shutdown effect by blocking ion flow and slowing the battery's internal

reactions. When the polymers eventually melt, they fully disconnect the anode and cathode from one another, while the higher-melting PP layer helps maintain structural integrity for longer, allowing multilayer PE–PP separators to use their coordinated melting behavior as a built-in thermal protection mechanism.[102,104,106,107,111,116] However, if the temperature continues to rise, the PP layer may eventually lose its mechanical integrity, allowing the electrodes to come into direct contact and thereby initiating an internal short circuit.[107,111,114,116]

3.4.2.3. Separator coatings – ceramic coating for increased thermal stability

As noted above, increasing the overall energy density of a battery can be achieved by minimizing the volume allocated to the separator's inactive material.[116] In addition, low thermal stability may be a safety hazard. Therefore, although the "PP-PE-PP sandwich" is common, it cannot be used for all types of batteries.[107,115]

To increase the performance of the separator, a ceramic coating of the main separator (i.e., base film) is considered promising[102,103,106–108,111–113,115,116] and it is already implemented in some industrial applications.[103,109] In this coating, micro- and nano-sized ceramic particles are incorporated into the polymer membrane.[102] The coating can be applied on the cathode or anode side, or both.[109,116] Coating with other ceramic (non-inorganic) or metal coatings is considered to have limited scalability.[115] Examples of ceramics coated on a base film separator include Al_2O_3, TiO_2, ZrO_2, AlOOH and SiO_2.[102,104,107,111,114–116] These ceramic oxides have high melting temperatures and excellent electrolyte affinity.[102]

The use of ceramic coating increases the thermal stability and higher electrolyte uptake, as well as the mechanical strength avoiding the risk of short circuit due to Li metal build-up at the anode (so-called dendrites).[102–104,106,109,114] However, a separator requires uniformity, with even distribution of ceramic particles which may be difficult to achieve during manufacturing.[116] In addition, coating with ceramic particles could increase the thickness, obstruct the porosity, and increase the ionic resistance.[102,104,108] The adhesion between the ceramic particles and base film could be lost at high temperatures.[108,115,116] A ceramic-coated separator is also more costly than single or tri-layer microporous membranes considering the manufacturing is more complex, where scale-up or higher mass loading (cylindrical compared to coin cells) could be an issue.[111] However, when comparing to other separator types (see below) it could still be considered a reasonable trade-off for improved performance.[102,111]

To facilitate coating, a binder (e.g. PVDF, its co-polymers or PVA) and additives can be used for mixing and coating,[102,104,107,110,111,114,116] but other processes are also possible e.g., so-called grafting.[102,111–113,115] Binder-free ceramic-coated separators are also reported.[112]

3.4.2.4. Other separator materials and designs

In the identified works, several alternative material and designs were mentioned. The review articles adopted a variety of terms to describe them (apart from the polyolefin/microporous membranes), where categorization was conducted based on the chemical composition of the base film, the fabrication process, the number of layers, or function provided, among others. In this work, we will not discuss these other types in detail, considering that they are mostly relevant only on research level.

Apart from the PE and PP single layer or base film material, some alternatives were reported in the literature. These included cellulose and co-polymers,[102,115] PVDF,[102–104,110,112,115] PVDF co-polymers,[103,110,112] PTFE,[102,104,115] PEO,[102,103,112] PE,[102,112] PVC,[102] PAN, [102,103,110,112], PEEK,[115] PVA,[115] PMMA, [102,103,110] among others.

Of these, PVDF and its co-polymers were reported to have been researched intensely over the years. This material possess has good (electro)chemical stability and electrolyte wettability, avoiding the additional challenges of a coated polymer.[106,110,112] Depending on the final application, it has been considered both thermally stable[104,115] as well as having low thermal

stability.[111,113] However, mainly due to the low mechanical strength as well as its high cost., PVDF and its co-polymers have rarely been used as single film separator.[106,110,112,113,115]

PVDF can also be used in combination with composites or blends.[110,112] In composites, it uses e.g. ionic liquids such as TFSI or metal organic frameworks or ceramic particles as fillers.[103,110] As stated above, it is mostly used as a binder for modification of traditionally used separators[112] and can be considered a coating on commercial separators to improve the wettability.[110]

General categories of separators apart from the simple microporous membrane include, but are not limited to, nonwoven mats,[102–104,106,107,110], modified microporous and other porous membrane separators,[102,112] composite membranes,[102–104,106,107,110] surface-modified membranes,[103,104,107,109–111] polymer blends,[103,107,110] among others. Some consider gel or solid-state electrolytes as a third phase separator.[102,103,106,109] Function-specified categories include e.g. heat-resistant substrate separators[111] and processed-based categories could include electrospun membranes.[103,110] Of these, many have limited commercial application. For example, studies are inconclusive whether nonwoven mats can be scaled.[102,103]

3.4.2.5. Separators in battery applications

Requirements for separators, and the subsequent choice in materials and processes, are set for each individual application.[108] For some applications, a monolayer of polyolefin is considered a sufficient separator, but it has limited use in high-energy and high-power applications, where advanced separators might be necessary.[109,112] For electric vehicles, fast charging together with thermal and mechanical stability is required, combining the needs of high safety and excellent performance.[112,115] Separators in EV applications must therefore have high wettability and low thermal shrinkage/high thermal and mechanical stability [106,112] On the other hand, flexible devices set other requirements for separators.[108] In consumer electronics, energy density is crucial which can be obtained with a thinner separator.[107] Luiso and Fedkiw (2020) noted that it has been reported that *Panasonic* and *Tesla Motors* use ceramic-coated separators with aromatic polyamide of *Sumitomo* brand.[106]

3.4.2.6. Notes on separator coatings in Na-ion batteries

Babiker et al. (2023) summarized not only the requirements for separators in Li-ion batteries, but also in Na-ion batteries. They stated that overall, microporous membrane separators "are at the heart" of Na-ion batteries and list the same technical requirements for Li-ion batteries as for Na-ion batteries. Therefore, the conclusions listed above that are relevant to Li-ion batteries could be extended to also relate to Na-ion batteries.

However, apart from the different cathode materials, some slight differences exist. For example, Na-ion batteries usually use a hard carbon as anode and a polypropylene carbonate electrolyte. This electrolyte has a high viscosity, which results in a poor wettability for the commercial PE and PP separators.[102]

3.4.2.7. Notes on separators in other battery technologies

As described above, some studies noted that solid or semi-solid electrolytes can function in a way that replaces the traditional separator in a battery.[102,103,106,109] Of the identified academic works (SI2), many were specifically discussing the use of separators in Li-S batteries, indicating that these have special requirements. However, this battery technology was not qualified as a potential PFAS use in our study.

3.4.3. Case 3: Li-SOCl2/Li-SOCl2/Cathode (potential PFAS use no. 3)

A total of 18 studies were identified as eligible of which 14 were included for the third case of survey of academic literature. As described above, this also includes reports from Google Scholar, thus extending into grey literature.

3.4.3.1. The history of the Li-SOCl2 battery

In literature reviews published between 1979-1988, the Li-SOCl2 battery was described as a "major research development" and "significant electrochemical power sources".[117,118] The battery was first developed with SOCl2 as a solvent in 1969 and scale-up of this primary battery was enabled a few years later.[118,119] At the time, most relevant applications of Li-SOCl2 batteries were in the military and medical sectors, taking advantage of high safety, high energy density, high specific capacity and large working temperature range as shortcomings of irreversible side reactions and energy loss was considered less critical.[118–120]

3.4.3.2. Li-SOCl2 battery technology and chemistry

The Li-SOCl2 battery consists of a lithium metal anode, a porous carbon cathode containing the thionyl chloride solution which forms associate pairs with tetrachloroaluminate.[117–119,121,122] Electrical energy is harvested when the SOCl2 is reduced into S and SO2 in a two-electron process, enabling the high energy density.[119,123] In this battery, the electrolyte also behaves as cathode reactant.[117,118,120] In addition, a separator is included to isolate the electrodes while conducting ions.[118]

3.4.3.3. Li-SOCl2 battery technology today

Up until today, the Li-SOCl2 battery has been superior in providing very high energy per unit weight and volume, operating even in extreme temperatures and offering a long shelf life of more than 10 years and high safety.[117,119,121–125] However, the reduction of this solvent cannot occur at high rates, limiting fast discharge.[121,123,126] It is a primary (non-rechargeable) battery.[119,121] As a result, the applications of these batteries are for those that demand maintenance-free operation and/or controlled environments, such as implantable medical devices, deep-sea applications, aerospace and utility metering.[119,120,122,124,127–129] Furthermore, they remain in the military sector which now also include cells that can discharge faster.[120,127,130] They are not very common in consumer applications.[121,122,124,125]

3.4.3.4. Cathode binders in the Li-SOCl2 battery

In the literature review, a cathode binder made of fluorinated material is commonly discussed. Overall, the use of carbon cathodes is neatly summarized by Gao et al. (2024) which state that "generally, the widely used carbon cathode substrate is a combination of carbon powder and polymer binders through different mixing methods" although they do not specifically mention any fluoropolymers.[120] Gangadharan et al. (1979) state that "colloidal Teflon is used as the binding material for fabricating the carbon electrode" which provides insulation to the electrode.[117] Hills and Hampson (1988) mention that the "inert electrode is usually made from amorphous carbon powder with a PTFE binder, the filamentary nature of which minimises blockage of the active carbon surface" with the purpose of giving mechanical stability obtained either by pasting through hot pressing or by rolling.[118] Dampier in a report of the US Air Force report that Teflon is present in the thionyl chloride battery, which can be presumed to be the binder.[126] Amabile et al. report that "porous teflonated carbon pads" meaning an "emulsion with a powdered material, adding methanol to coagulate the Teflon®, followed by cleaning and drying" are used as cathodes for the Li-SOCl2 batteries.[127] In addition, Lu et al. (2023) state that "porous carbon cathode is usually prepared by coating the mixed slurry of carbon powder and polytetrafluoroethylene (PTFE) binder on the current collector".[123]

On the other hand, two records mention that there are alternatives to PTFE binders in Li-SOCl2 batteries, namely Gangadharan et al. (1979) which report that polyethylene, paper pulp and others are used[117] and Hills and Hampson (1988) describing chlorinated hydrocarbons which have low contact angles and good chemical stability,[118] although these works could be considered outdated for the purpose of this study.

3.4.3.5. Notes on separators in the Li-SOCl2 battery

Furthermore, no works mentioned uses of fluorinated materials as separators. Instead, Gangdharan et al. (1979) described that the separators could be made of nonwoven glass sheet,

nonwoven rayon, glass filter paper or porous polyethylene sheet.[117] A military report from the US Air force written 1985 lists the use of glass separators in Li-SOCl2 batteries. Here, they describe that there are glass fiber separators with organic binder materials such as polyvinyl alcohol. However, they state that binder-less separators (Lydall No 991) are available and could be implemented.[126] Although these works were written before 1990 and developments are expected to occur since, more recent works concur. Loupe at al. (2017) report the use of a nonwoven glass fiber separator.[125] Similarly, Roth et al. (2024) describe that “a glass fiber material that acts similar to a separator, preventing the carbon cathode from shorting.[122]

3.5. Potential alternatives as stated in Annex XV consultation comments

The stakeholders not only submitted information on uses of PFAS; they also provided evidence of potential alternatives to PFASs in batteries, which we aim to summarize in this section. Apart from direct evidence, we extrapolate in several cases that alternative options may exist for a given application even when stakeholders do not mention them explicitly. For example, if a stakeholder reports that a particular alternative is unsuitable for the high voltages associated with a specific chemistry, we assume that the same substance or material could still be viable at lower voltages. Likewise, when stakeholders argue that fluorine-based substances are necessary, but those substances fall outside the PFAS definition, we assume that these fluorine-based substances can provide the required function and therefore represent a PFAS-free alternative.

In our work, we define an alternative for batteries as a PFAS-free substance, material, design, process or technology on the market. Substance and material alternatives are related to the Battery Use Level 1, i.e. the component, in which the technical function for the PFAS can be fulfilled by the PFAS-free alternative. Furthermore, it is possible to alter the design (e.g., configuration) or (manufacturing) process to obtain the same technical function. We include information on configuration and manufacturing process in the database in parenthesis under Battery Use Level 2. A PFAS-free alternative could also be obtained by substituting a battery technology using PFAS to a battery technology using other-than-PFAS substances as long as the function and technical requirements are fulfilled.

3.5.1. Alternative substances and materials – comparison on Battery Use Level 1

3.5.1.1. Cathodes

The Fluoropolymers Product Group (FPG) reported that “Several polymers, including polyacrylic acid, sodium alginate, polyurethane, and catechol-bearing polymers have been assessed as cathode binders for Li-ion batteries”, but it is unknown to which chemistry they referred. (#6150) In addition, Chemsec notes that *Nanoramic* provides a binder with 3D carbon binding structure, that is both conductive and binding (#8863) and *Innovationsallianz* describe the use of Ni as a metallic binder (#9076).

VARTA reports that Li-ion batteries of LFP chemistry would be compatible with water-processed binders, particularly at low voltages.(#9410) Water-based binders are also possible in other chemistries, illustrated by the project DigiBatt Pro (funded by the German government) which successfully produced Li-ion batteries of NMC chemistry cells at a smaller scale, although the results were limited to high Ni content.(#8680, #9410, #8897) Another project, ÖkoMatBatt, produced Li-ion batteries of unknown chemistries with PAA and other polymers as binder, which provided promising results with high binder volumes. However, these results were not transferable to chemistries with high Ni content. (#8680, #9410) Furthermore, *Eurelectric* report that “For processing requirements, cost and environmental issues, battery manufacturers are gradually moving from using PVDF to hydrophilic binder materials such as SBR copolymers, that can be used for practically all Li-ion cell chemistries – for both the anode and cathode electrodes, without change of electrochemical properties. Although the replacement of PVDF for aqueous binders is desirable and at the core of the

efforts of several cell manufacturers, to date this goal has not been achieved and it is not possible to find suppliers of batteries without PVDF."(#6443) Thus, there is conflicting evidence from the stakeholders regarding the feasibility of substituting PVDF in Li-ion battery cathodes.

Northvolt argues that Na-ion batteries of PBA chemistry are not sensitive to water and could function with CMC/SBR and carbon. (#6133, #8801) However, *RECHARGE* reports that CMC/SBR is used in Na-ion batteries on research level. (#8680) Meanwhile, Na-ion batteries of layered metal oxide chemistry would require PVDF based on the same requirements as for Li-ion batteries based on the same chemistry. (#8801)

3.5.1.2. Anodes

Several stakeholders mention the anode binder today consists of a mixture between CMC and SBR for Li-ion batteries, particularly with graphite anode chemistry. (#8897, #8680, #8010) Batteries that use a metallic anode, both primary (Li-SOCl2, Li-SO2, Li-MnO2) and secondary (lithium metal batteries, either solid state or with liquid electrolyte) do not require a binder.(#8680)

Several stakeholders state that PFASs are used in e.g., Li-ion battery (dry or semi-dry process), Li battery (primary), NiCd battery, NiMH battery, Zinc oxide battery, Metal air battery, Silver oxide battery, Zinc-ion battery (secondary), Lithium metal (secondary) and Solid-state battery *except for* the graphite anode.(#8632, #8215, #8871, #9483, #8680) Therefore, we conclude that the anode material in Li-ion batteries is PFAS-free according to stakeholders.

3.5.1.3. Electrolytes

Several fluorinated electrolyte salt and solvents were claimed to not be possible to substitute, including fluorobenzene (#4331), FEC (#6957, #6100, #4346, #4370), $LiPF_6$ (#9078, #7761, #9246), and $NaPF_6$ (#7761). Although the specific function of each substance is unknown, as is the requirements for the electrolytes in the various battery technologies and chemistries, this list points to the possibility of using non-PFAS fluorinated substances.

One stakeholder described that solid-state batteries could use the ceramic polyethylene oxide (PEO) as electrolyte.(#8680)

3.5.1.4. Separators and separator coatings

According to *RECHARGE*, separators are commonly based on PE, PP and/or cellulose, in one or several layers without a coating.(#8680) However, coatings are sometimes used, in which PVDF is used for the ceramic coating.(#8897) Several stakeholders report that alternatives for this PFAS use are available, though the expected timelines differ.(#8215, #9410, #8828) One example mentioned for adhesion is PMMA.(#8680) Separator coatings may still be needed in applications or manufacturing processes that involve high temperatures according to stakeholders,(#9410, #8680, #4331) where adhesion could otherwise be lost. The *Battery Association of Japan* explains that in cylindrical cells, the winding process can provide sufficient adhesion without PVDF, whereas in lamination-based manufacturing, PVDF remains the only viable material.(#4331)

Innovationsallianz stated that they were developing an acrylate-based separator for Li-S batteries, although at the time of submission it was in early stages.(#9076)

3.5.1.5. Gaskets and other non-cell-specific components

Several non-fluorinated polymers were listed as potential alternatives to fluoropolymer-based gaskets (#4331). PP, PPS, PEEK and FKM may be performing better than PFA (#4574, #8563). *RECHARGE* reports that PTFE are not used as gaskets in Li-MnO2 and that PBT and PEI would "provide an adequate sealing performance" in applications of mild temperature ranges (#8680). Furthermore, PP is stated as possible material, except for high-rate discharge applications (#4331) or in aerospace applications (#6150). *EPTA* mention that some alternatives are in development, but do not state which materials (#8828) and *ZVEI* report that "PTFE, FEP, PFA, VDF, HFP, FKM in valves, gaskets and washers of batteries might be

replaceable in the long-term, as alternatives are development – However, more time is required for substitution." (#8215) Here, we note that VDF and HFP most likely refers to the polymeric types of these monomers, either as the homopolymers or a co-polymer.

3.5.2. Alternative design, process and technology – comparison on Battery Use Level 2

3.5.2.1. Primary batteries

One stakeholder indicated that alkaline batteries and carbon-zinc batteries were considered PFAS-free alternative technologies instead of Li-SOCl2 batteries. However, as these differ significantly in terms of temperature and long-term stability as well as energy density, they would not be suitable alternatives for all applications.(#4651)

3.5.2.2. Secondary batteries

Several stakeholders mentioned that lead-acid batteries do not use fluoropolymers and that they therefore could be considered as alternatives to Li-ion batteries. However, they also report that lead acid batteries could be considered regrettable substitution due to the hazardous properties of lead, currently facing regulatory scrutiny. In addition, they reported that lead acid batteries would not fulfill the requirements posed to Li-ion batteries in terms of energy density.(#7499, #9188, #4331, #8680, #8372, #6603, #7427, #6050, #6181). Another technology suggested as alternative to Li-ion batteries, implied to have low content of PFASs, is that of NiMH batteries; however, the voltages (~1.2V) are much lower than what is provided by Li-ion batteries (~3.6V) and thus not provide the same function at the technical requirements for the in the application of interest (#4331).

However, a change of cathode materials could enable PFAS-free Li-ion batteries. While several stakeholders report that NMC, LCO, and NCA are sensitive to water(#7052, #8680, #8897), some projects showcased successful results.(#8680, #9410, #8897). LFP was stated to be compatible with water-based binders.(#9410)

Similarly, as reported above, the cathodes of Na-ion batteries could be PFAS-free if using PBA cathode chemistry. For example, *Northvolt* state that "PBA-based sodium-ion batteries are expected not to use PFAS-based components in their electrode"(8801). As a result, the Na-ion battery of PBA chemistry could be an alternative technology to Na-ion battery with layered metal oxide chemistry. In addition, Na-ion batteries could be used in many of the applications commonly adopting Li-ion batteries, and thus PFAS-free Na-ion batteries could be considered potential alternatives to Li-ion batteries for certain applications such as energy storage or short-range mobility applications with cost as deciding factor.(#8801)

VARTA also states that Li-ion batteries in power tools, hearing aid, and energy storage have alternatives available on TRL 3-4. Although their cathode chemistry is unknown, this indicates that alternatives for end-uses of high-performance requirements (e.g. power tools) could have PFAS-free cathodes in the future.(#9410)

4. INTEGRATION OF LINES OF EVIDENCE

4.1. Li-MnO2 battery

Two potential PFAS uses were listed for Li-MnO2 batteries: Li-MnO2/Li-MnO2 chemistry/cathode based on sufficient information from one record and Li-MnO2/Liquid electrolyte/Electrolyte based on sufficient information available in five records(Table 4; potential PFAS uses no. 1-2).

Li-MnO2 batteries are sometimes linked to the "CR" terminology. Although they contain both manganese and lithium, they are different from e.g. alkaline manganese batteries (primary) or other lithium batteries (primary or secondary). For example, some reports of the use of PFASs in batteries state a MnO2 battery treated with fluorinated surfactant[4,9,14] or short-chain PFSAs in the form of their lithium salts in battery electrolytes.[25] Mentions of PFASs in "lithium batteries" was particularly prevalent without a further explanation of the battery technology or

battery type.[4,9,13,23] In addition, one study reported the use of primary lithium batteries, e.g., CFx as lubrication in primary lithium batteries.[15,22] The Background Document lists uses of PFASs in primary lithium batteries and also outlines specific primary battery types, such as "alkaline button (LR)."[11] Furthermore, the OECD fluoropolymer report[20] includes one Annex XV consultation comment (#4331), and therefore lists similar uses.

In sum, many of the results from previous use-mapping studies cannot be applied in this work to conclude on the use and potential for alternatives in primary batteries in general or in Li-MnO2 batteries in particular. As a result, we conclude that…

- The (lack of) relevant supporting evidence could not confirm or contradict the listed potential PFAS uses of Li-MnO2/Li-MnO2 chemistry/Cathode and Li-MnO2/Liquid electrolyte/Electrolyte preventing also a conclusion on the availability of potential alternatives.

4.2. Li-SOCl2 battery

One potential PFAS use was listed for Li-SOCl2 batteries: Li-SOCl2/Li-SOCl2 chemistry/Cathode based on sufficient information available in two records(Table 4; potential PFAS use no. 3).

Similar to other listed potential uses of primary batteries, no information was available except from the Background Document. However, this potential use was selected as a case study for the survey of academic literature and based on the literature review, see section 3.4.3.. The Li-SOCl2 battery consists of a lithium metal anode, porous carbon cathode and a thionyl chloride solution. Because it is a two-electron process, where the SOCl2 is reduced to S and SO2, it has high energy. However, this reaction is not reversible. Several studies and technical reports, both from early stages of battery development (around 1970-1980) and current, identify that PTFE (or Teflon) is used as the binder in the carbon cathode.[117,118,120,123,126,127]

Information was only available from one of the four supporting evidence types, namely the academic literature search, strengthening the weight of the evidence. As a result, we conclude that…

- it is *very likely* that Li-SOCl2/Li-SOCl2 chemistry/Cathode is a **PFAS use** and overall, *no potential alternatives* were identified for the confirmed use.

4.3. Li-SO2 battery

One potential PFAS use was listed for Li-SO2 batteries: Li-SOCl2/Li-SO2 chemistry/Cathode based on sufficient information available in one record (Table 4; potential PFAS use no. 4).

Li-SO2 batteries are not described in any of the supporting evidence, except for the Background Document listing it briefly, which most likely stems from the same Annex XV consultation comment as included in this work. As a result, we conclude that…

- The (lack of) relevant supporting evidence could not confirm or contradict the listed potential PFAS use of Li-SOCl2/Li-SOCl2 chemistry/Cathode preventing also a conclusion on the availability of potential alternatives.

4.4. AgO2 battery

One potential PFAS use was listed for AgO2 batteries: AgO2/AgO2 chemistry/Cathode based on sufficient information available in one record (Table 4; potential PFAS use no. 5).

Apart from the Background Document noting the use of electrode binder in in AgO2 batteries, none of the previous use-mapping studies or other supporting information provide insights to conclude on the likelihood of PFAS use or availability of alternatives. As a result, we conclude that…

- The (lack of) relevant supporting evidence could not confirm or contradict the listed potential PFAS use of AgO2/AgO2 chemistry/Cathode preventing also a conclusion on the availability of potential alternatives.

4.5. Li-ion battery

15 potential PFAS uses were listed for Li-ion batteries: a total of six related to the cathode, two each describing the anode, electrolyte and separator as well as three related to non-cell-specific components (Table 4; potential PFAS uses no. 6-20).

4.5.1. Cathode

Six potential PFAS uses were listed for Li-ion battery cathodes: the five in Li-ion/LFP, LCO, LMO, NMC, or NCA chemistries/Cathode based on sufficient information available in five, two, two, four and two records, respectively, and Li-ion/Metal oxides/Cathode, coating based on sufficient information from one record (Table 4: potential PFAS uses no. 6-10).

The binder in Li-ion batteries was often reported in the supporting evidence. In 2013, Ebnesajjad stated that "PVDF homopolymers and copolymers are used in the battery industry as binders for cathodes and anodes in Li-ion batteries."[15] Thereafter, Glüge et al. (2020) described the use of PVDF and FEP in both anode and cathodes, also identified by Rensmo et al. (2023).[4,16] Recent use-mapping studies also reported the use of binder in the Li-ion battery electrode.[18,20,21] In addition, several studies mentioned the use of binders in cathodes citing older sources.[9,12,19] The Annex XV report and the Background Document listed the use of cathode and anode binders in Li-ion batteries.[10,11] In addition, some stakeholders along the battery value chain reported the use of PVDF and PTFE in Li-ion cathode binders.[36,40,50,58,63,66]

Apart from the Background Document noting the use of electrode binder in LFP, none of the use mapping studies include information about the Li-ion battery chemistry (i.e., cathode composition). Complementing evidence arises from the teardown studies, in which peer-reviewed studies provide insights into the material composition of on-the-market batteries. In these studies, electric vehicle batteries (*Tesla Motors*, *BYD*, *Volkswagen*), and small batteries (*Samsung*, *LG*, unknown) were taken apart and analyzed for their contents. However, these often focused on the inorganic substances, and nine of the included studies did not indicate the material in the cathode binder. Still, PVDF was identified by Gorsch et al. (2025) in LFP prismatic batteries from the Blade model from *BYD* (via TGA)[70] and Kong et al. (2022) in LCO commercial pouch cells of unknown manufacturer (via EDX).[77] On the other hand, while Gorsch et al. (2025) characterized carboxymethyl cellulose (CMC) and styrene butadiene rubber (SBR, via TGA),[70] Ank et al. (2023) detected PVDF in the cylindrical NMC with 80% Ni cathode of *Tesla Motors* Model Y.[71] Furthermore, "no fiber-like morphology" was identified by Liu et al. (2025) that the (Ti-doped) cylindrical LFP batteries from BYD, indicating that PTFE is not used.[69] In sum, the teardown-analysis studies were inconclusive, with certain studies indicating PFAS use while others noting substances other than PFAS for the same component in the same model of a commercial battery.

Considering the large amount of available information describing the binder as a function in Li-ion battery cathodes, both from the database and additional information, it was selected as one of the case studies for surveying the academic literature. However, only one potential use and function was investigated (Li-ion/LFP chemistry/Cathode, technical function: "binder"), described in section 3.4.1. Following these results, our interpretation is that PVDF is most used in Li-ion batteries in general, as well as Li-ion batteries of LFP chemistry in particular. These studies described that PVDF has a high mechanical strength, good electrochemical stability and good wettability, resulting in a uniform matrix for the electroactive material which influences performance criteria such as cycle life and charging.

However, PVDF is not electronically conducting which necessitate the use of a carbon additive in the cathodes, adding "dead weight" to the battery and reduces the overall energy density. In

addition, PVDF has limitations in high-performance batteries, resulting in loss of adhesion. It also requires the use of NMP (a teratogenic solvent), which impose strict conditions for handling as well as large infrastructure for removing the solvent. Therefore, multifunctional binders and alternative manufacturing techniques are investigated, where dry processing, is one option. The latter often involves PTFE, which provides a binding function by forming a net-like structure of fibers, or binder-free processes with thin cathodes and/or nanoparticles.

Another solution proposed in the academic literature was the use of water-based processing. Rolandi et al. (2024) described that LFP has low sensitivity towards water due to the strong Fe-P-O bonding.[131] This was compared to the NMC and NCA chemistry, in which the transition metals could dissolve leading to reduced performance. Stabilization is required to protect the transition metals. Still, Park et al. (2025) reported that both LFP and "some NCM-based" cathodes are manufactured with water-soluble binders, although scaling was considered a challenge.[90]

During the interviews with stakeholders along the battery value chain, the cathode binder was mentioned as a common use of PVDF. PTFE was briefly mentioned, but reported to be difficult to commercialize, partially since this would involve completely new production lines.[36]

Several battery stakeholders in our study provided alternatives to the use of PFASs in batteries. *Ateios*, founded in 2018, provides a technology that does not use fluoropolymers and removes the need for a solvent (e.g., NMP) in which a "readily available" monomer is crosslinked into a matrix with the active material. This binder has been applied in LFP, LCO, NMC and NCA batteries, up to the standard-LCO voltage at 4.53.[36] *Celanese*, a global chemical and specialty materials company, provides a product with polar acrylic monomer and ethylene backbone, which has a thermal and electrochemical stability up to 4.2 – 4.5 V. However, they stated that as of 2026, this can only partially substitute the use of PVDF.[40] *GRST*, a producer of binders for battery electrodes, has a factory at a GWh scale. Their material is water-soluble and compatible with LCO, NCA and LFP cathodes. It can also be used in water-based processing of NMC with up to 60% Ni as well as NMP-based processing of NMC with up to 80% Ni.[48] *Leclanché*, founded in 1909 and focusing on Li-ion batteries since 2006, has produced batteries without PFASs in the cathode binder since 2012. Their batteries are based on the NMC chemistry with 60% Ni as well as the NMCA (NMC + low concentration of Al) up to 4.34 V.[58] The binder is based on CMC in a mixture of other binders, such as PAA and SBR together with a set of surfactants.[59] Furthermore, *RECHARGE* reported that cathode binders based on acrylic acid have shown promising results.[63]

The availability of information from supporting evidence, both in terms of overall results as well as information available from several source types, was considered high for the five potential PFAS uses of cathodes in Li-ion batteries. However, in terms of the coating of cathodes metal oxide chemistries, no information about this was identified in the second stage of this work. As a result, we conclude that…

- it is *very likely* that Li-ion/LFP chemistry/Cathode, Li-ion/LCO chemistry/Cathode, Li-ion/LMO chemistry/Cathode, Li-ion/ NMC chemistry /Cathode, and Li-ion/ NCA chemistry /Cathode are **PFAS uses,**
- *several potential alternatives* were identified for the use Li-ion/LFP chemistry/Cathode while *some potential alternatives* were identified for the other four potential PFAS uses other potential PFAS,
- the (lack of) relevant supporting evidence could not confirm or contradict the listed potential use of Li-ion/Metal oxides/Cathode, coating preventing also a conclusion on the availability of potential alternatives.

4.5.2. Anode

Two potential PFAS uses were listed for Li-ion battery anodes: Li-ion/Graphite anode in LFP, LMO, NMC, and NCA chemistries/Anode and Li-ion/NTO anode in LTO chemistry/Anode based on sufficient information available in one record each (Table 4; potential PFAS uses no. 12-13).

Some of previous use-mapping studies briefly mention the use of PFASs in the binder of both anodes and cathodes.[4,9–11,15,18,21] However, following the inquiry for information on market use of PFASs, *Ashland* stated that CMC is used in the majority of gigafactories in Europe, most likely linked to the common battery chemistries (LFP, LCO, NMC, NCA and possibly LMO). This company, a global manufacturer of additives and specialty ingredients, provides materials for anodes. Their CMC-based product used for dispersion of conductive additives in water-based formulations can be modified for a range of conditions.[33] Other battery stakeholders also noted the use of CMC/SBR in the anode, including Westra Materials.[66]

A total of five teardown-analysis studies characterize the anode binder material. The *BYD* Blade cylindrical and prismatic LFP batteries were found to contain a slurry-based CMC and water-processed CMC with SBR, respectively.[69,70] Both anodes in a NMCA cylindrical battery from *LG* and a NMC with 80% Ni pouch cell from an unknown producer were separated in the teardown procedure using water-based processes, indicating a non-PFAS based binder.[75,76] However, Stapf et al. (2025) concluded the use of PVDF in the anode binder of *LG* M50T and M50LT, both of NMC with 80% Ni (via XRD and EDS).[74] As reported in the section on Li-ion battery cathodes, the *Tesla Motors* Model Y cylindrical NMC with 80% Ni battery was analyzed by both Gorsch et al. (2025)[70] and Ank et al. (2023),[71] who concluded on two different anode binders, PAA and PEO (via TGA) and PVDF or PTFE (via EDX), respectively. All anodes in the teardown studies were based on a graphite (or carbon, which is considered the same) anode, of which a few included a low amount of Si.

In the survey of academic literature, investigating the use of cathode binders in Li-ion batteries of LFP chemistry, some literature reviews also mentioned the use of anode binders in Li-ion batteries, where all described that CMC and SBR are most prevalent (section 3.4.1.5). CMC is used to obtain a desirable viscosity in the slurry, while SBR functions as an emulsifier. This is because the anode is not sensitive to water and the water-based processes are preferred due to low cost and minimized hazards.

The downstream users who have mapped their uses of PFASs in their products did not mention anodes, indicating that Li-ion battery anodes do not contain PFASs.[50,53,62] Although LTO was not listed as a battery chemistry that was used by Marshall or IKEA, and it is unclear if it is used by HP, we consider it unlikely that this would change the conclusion. Thus, when assessing all lines of evidence, weighing that only one record resulted in the listing of graphite anode as a potential PFAS use and a few mentions in previous use-mapping studies, against the large basis of evidence both from industry actors directly involved in anode material production as well as teardown-analysis studies showcasing the use of CMC/SBR in commercial batteries, we conclude that…

- it is *very unlikely* that Li-ion/Graphite anode in LFP, LMO, NMC, and NCA chemistries/Anode is a **PFAS use**, and in extension, *potential alternatives are available in large scale*,
- the (lack of) relevant supporting evidence could not confirm or contradict the listed potential use of Li-ion/NTO anode in LTO chemistry/Anode preventing also a conclusion on the availability of potential alternatives.

4.5.3. Electrolyte

Two potential PFAS uses were listed for Li-ion battery electrolytes: Li-ion/Liquid electrolyte/Electrolyte based on sufficient information available in 23 records and Li-

ion/Polymer electrolyte/Electrolyte based on sufficient information available in three records (Table 4; potential PFAS uses no. 14-15). The database included information about 18 individual PFASs, excluding derivatives, in Li-ion battery electrolytes. It also included corresponding functions, such as antioxidant, solvent, corrosion inhibitor, conductive agent and other ("allow exchange of ions between electrons" and "suppress HF generation").

Most often, Li-ion batteries have a liquid electrolyte, which is overall not dependent on the choice of cathode and anode materials. Li-ion batteries can also have a polymer electrolyte to increase the energy density. As described in Rensmo et al. (2023), a liquid electrolyte often consists of a solvent and a main salt, e.g., $LiPF_6$, as well as additives.[16] Several of the consultation comments listed $LiPF_6$ as a substance present in the electrolyte.(#6443, #7761) In parallel, they noted that there were no alternatives for $LiPF_6$ in the electrolyte and that this should be excluded from the universal PFAS restriction proposal (which it already is as $LiPF_6$ is not a PFAS, but this might not be fully understood by the stakeholders). Thus, this could indicate that Li-ion electrolytes provide acceptable level of performance for certain applications even without PFASs such as LiTFSI.

The Annex XV report and the Background Document list electrolyte additives in Li-ion batteries, as does the report from the Danish EPA.[10,11,19] Rensmo et al. (2023) identified several lithium salts included in the OECD PFAS definition, namely those of triflate, TFSI, BETI, FTFSI. In addition, they also listed some relevant for laboratory scale experiments such as a perfluorinated analogue of $LiPF_6$, but it is uncertain if these have been commercialized.[16]

The anode surface investigated in teardown-analysis studies may provide insights into the content of the electrolyte. This is because in Li-ion batteries, a solid-electrolyte interface (SEI) is formed by-design at the anode surface from degraded electrolyte to provide a layer which protects the anode from further degradation while still allowing ionic transport. [16] Some of the teardown-analysis studies characterized this layer, including e.g., Stock et al. (2023) who reported that the fluorine residues were below 1%.[68] Kong et al. (2022), on the other hand, reported a fluorine composition of more than 5%.[77] Still, these results are inconclusive in terms of classifying the electrolyte, as non-PFAS fluorinated salts are expected to be present in the liquid electrolyte.

The composition of an electrolyte is highly dependent on the application of the battery and its requirements. This was explained by the *E-Lyte* representative who noted that the electrolyte formulation that they provide to their customers is tailored to each customer's needs.[43] In addition, *RECHARGE* also emphasized that technical requirements set for each application direct the choice of battery technology and composition.[63] For high-temperature stability, LiTFSI is applied as an additive. This could be in, for example, application of power tools.[43,63] However, also cost influences the selection of salts, in which LiTFSI is about ten times more expensive than $LiPF_6$, limiting its use in commodity applications.[43]

The polymer electrolyte was discussed briefly in the US DOE report stating that "gel polymer electrolytes" including PVDF and PVDF-HFP were used in Li-ion batteries.[21] It was also noted by *E-Lyte* that polymeric electrolytes could add LiTFSI to provide long term stability when operated at elevated temperatures.[43] In addition, the database record 278 (#4208) and database record 294 (#4340) list two PFASs (hexafluoroisopropanol, HFIP and lithium cyano(trifluoromethanesulfonyl)imide, LiCTFSI) that were added when a voltage above 4 V was required.

Overall, PFAS use in Li-ion battery electrolytes is described in detail by battery stakeholders, as well as previous use-mapping studies. Meanwhile, the teardown-analysis studies can only conclude on the presence of fluorinated substances in the electrolyte, but not that of PFAS. Although evidence of TFSI use could be perceived as conflicting (e.g. in some cases it is used while in others not) we have shown that this is due to the requirements for the application (i.e. Battery Use Level 3). As a result, we conclude that…

- it is *only likely for certain applications* that Li-ion/Liquid electrolyte/Electrolyte is a **PFAS use**, and *potential alternatives* were identified for *only for certain application(s) of use*
- the (lack of) relevant supporting evidence could not confirm or contradict the listed potential use of Li-ion/Polymer electrolyte/Electrolyte preventing also a conclusion on the availability of potential alternatives.

4.5.4. Separator

Two potential PFAS uses were listed for Li-ion battery separators: Li-ion/Liquid electrolyte/Separator based on sufficient information available in ten records and Li-ion/Liquid electrolyte/Separator, coating based on sufficient information available in seven records (Table 4; potential PFAS uses no. 16-17).

PFASs in Li-ion battery separators was reported in previous use-mapping studies, including Ameduri (2018), Ameduri (2020), Chemsec (2023) and OECD Synthesis report on fluoropolymers (2025).[13,18,20,23] However, in these studies, it is not reported if it relates to the base film or the coating. Meanwhile, Rensmo et al. (2023) and the US DOE report (2024) specify the use of PFASs in separators to the base film (i.e., a single material component).[16,21] Furthermore, the Annex XV report and the Background Document notesthe use of "separator films/coatings" in Li-ion batteries, as does Danish EPA report, basing its findings in the Annex XV report.[10,11,19]

Information related to the identity of the separator was given in four teardown-analysis studies. Chen et al. (2020) report that the separator was ascertained to be "ceramic-coated rather than a normal polyolefin (CnH2n) membrane" in a cylindrical battery from *LG* (via SEM and density analysis).[75] Guo et al. (2025) stated that it was likely that PP or PE was the separator in an unknown commercial NMC with 80% Ni pouch battery (via TGA).[76] Kong et al. (2022) concluded that the separator in unknown commercial pouch cells of LCO and NMC chemistries, stacked and jelly-rolled, respectively, contained a PP separator (via FTIR).[77] Lastly, Hamed et al. (2024) reported a fluorine peak in EDX for the separator, although did not conclude on the identity of this material.[79]

The use of separators in Li-ion batteries with liquid electrolytes was selected as one of the case studies for in-depth survey of academic literature. According to these studies, a separator is used in these battery systems to prevent physical contact between the anode and cathode while allowing ion transport, which is primarily linked to the safety of the battery. It must be electrochemically stable, have uniform permeability and low thermal shrinkage, among other aspects, and the choice of separator is highly dependent on cost. According to many of the included literature reviews, PP and PE dominate the market, due to their low cost which is closely linked to the ease of manufacturing and low cost of materials. In the interviews, Celanese reported that they provide an ultrahigh molecular polyethylene material that could be used in separators.[40]

However, PP and PE have melting points around 150°C, which is a limitation in batteries in high-performance applications. One solution has been to combine PE and PP in a tri-layer structure ("PP-PE-PE sandwich"), which at high temperatures enables a safety mechanism that shuts down the battery. However, if the temperature continues to increase, this mechanism fails. Another solution reported in the academic literature is to coat the separator with thermally resistant coating, most often ceramic particles such as AlOH, SiO_2 and Al_2O_3, which may require a binder. The literature reviews reported the use of PVDF as the most common material to obtain the binding function between ceramic particles and the (polyolefin) base film. This increases the thermal stability, but could increase the separator thickness, which decreases the energy density of the battery overall.

The use of other polymers (non-PFAS) as binders in separator coatings, such as PVA, as well as other processes, such as grafting was reported in the academic literature. In addition, battery

stakeholders also listed examples of potential alternatives in this use with this function. *KURZ*, involved in roll-to-roll coating products, stated that PVDF is not used as a binder in ceramic-coated separators, but rather polyacrylate-based binders today. This is mainly because of the large price difference between the two, where PVDF could be three to eight times more expensive.[56] For example, *Ashland* provides several non-PFAS binders for ceramic-coated separators, which they reported are required for thermal stability.[33] Luiso and Fedkiw (2020) noted that it has been reported that *Panasonic* and *Tesla Motors* use ceramic-coated separators with an aromatic polyamide binder (*Sumitomo*).[106]

When listing separator coating in Li-ion batteries as a use, some Annex XV stakeholders not only referred to the binding, but also linked it to the adhesion ("adhesion/cohesion promoter", mostly in records which report the use of a separator "layer", "sheet" or "interface between the electrode films and collectors"). In interviews with stakeholders from the battery value chain, adhesion between the separator and the cathode during the manufacturing of batteries was mentioned as another function needed for the use of PFASs in separator coatings in Li-ion batteries. That is, the binder would not be binding the ceramic particles to the separator but provide adhesion of the separator to the cathode.

The *KURZ* representative described this as adhesion coating.[56]. Adhesion coating may be required particularly in automotive applications because in these testing protocols, temperatures exceed that of significant PE shrinkage.[56] For example, Leclanché reported that they use PVDF in the lamination step of their production line.[58] Marshall, as a downstream user, reported that their separator contained PFASs.[62] Similarly, Chemsec describe the use of "perfluorocarbon" separators in batteries requiring high voltages. Although it can be assumed that these are linked to the use in coating of separators, it is not known if it provides the function of adhesion or binder.[18]

KURZ described the use of a co-coating between ceramic and PMMA materials with a thickness of about 2-5 µm. They provide this solution to their customers, and it has already been commercialized by their joint venture collaborator for two years.

On the other hand, the need for adhesion coating can be eliminated by different manufacturing process according to *KURZ*. In cylindrical cells, the electrodes are tightly wound already, providing necessary mechanical strength, unless the technical requirements specify a particularly long cycle life. In addition, they state that Z-folding would require adhesive coating.[56] Leclanché, on the other hand, stated that substituting their Z-folding process step, they would removing the need for the adhesion coating but still produce batteries with sufficiently long cycle life.[58]

Overall, previous use-mapping studies provided some evidence to link the use of PFASs in a separator either to the microporous membrane or to the coating. On the other hand, teardown-analysis studies provided strong evidence that the separator is made of PE and/or PP. Meanwhile, battery stakeholders emphasize that the base film on the market is not PFAS-based. These sources, together with academic literature, indicate that the separator coating is used for certain requirements, although determining these requirements is beyond the scope of this work.

As a result, we conclude that…

- it is *very unlikely* that Li-ion/Liquid electrolyte/Separator is a **PFAS use** and, in extension, *potential alternatives in large scale*
- it is *only likely for certain applications* that Li-ion/Liquid electrolyte/Separator, coating is a **PFAS use** for which *some potential alternatives* are identified.

4.5.5. Non-cell-specific components

Three potential uses were listed for non-cell-specific components in Li-ion batteries: Li-ion/Cell/Seal gasket, Li-ion/Cell (sub-assembly)/Lubricant and Li-ion/Pack (outer packaging)/Vent based on sufficient information available in one, two, and one record(s), respectively (Table 4; potential PFAS uses no. 18-20).

No supporting evidence was identified for these three potential uses. As a result, we conclude that…

- The (lack of) relevant supporting evidence could not confirm or contradict the listed potential use of Li-ion/Cell/Seal gasket, Li-ion/Cell (sub-assembly)/ Lubricant and Li-ion/Pack (outer packaging)/Vent preventing a conclusion on the availability of potential alternatives.

4.6. Na-ion battery

Overall, the Na-ion battery technology is similar to that of a Li-ion battery, being developed as an alternative with the advantage of low cost, closely connected to the non-use of transition metals with geopolitical and abundancy limitations. The Annex XV consultation comments reported that the Na-ion battery technology is similar to the Li-ion battery technology, with the sodium salt of PF_6 being used as the main salt. (#7761, #8001) In addition, one Annex XV consultation comment stated that the substance Na3V2(PO4)2F3 offers a high reversible capacity at 4.2 V.(#7761)One of the Annex XV consultation comments, *Northvolt,* described that the Na-ion batteries are suitable in energy storage systems and short-range mobility applications when low cost is preferred over high energy density.(#8801)

4.6.1. Cathode

Three potential uses were listed for Na-ion battery cathodes: the three Na-ion/Layered metal oxide, PBA, or Phosphate-based chemistries/Cathode based on sufficient information available in two, one and one record(s), respectively (Table 4; potential PFAS uses no. 21-23).

Northvolt reported in the consultation that "sodium layered metal oxide /…/ is dependent on PFAS substances in the same way as Li-ion batteries because [layered metal oxide] uses the same manufacturing technology as Li-ion." (#8801) As no other information was available regarding PFASs in this use, it can be assumed that the conclusions for the Li-ion battery related to metal oxides could be extended to the Na-ion battery with layered metal oxides.

Otherwise, information about use of PFASs was only identified for PBA in the second stage, by Annex XV consultation comments referring to alternatives. *RECHARGE* stated in their Annex XV consultation comment that "PVDF is preferred with some other PBA materials and with hard carbon" and argued that "some research is ongoing regarding non-PFAS SBR/CMC binder materials for some hard carbon/PBA cells but this research work has not yet been scaled up."(#8680) However, *Northvolt* argued that "The electrode of batteries using PBA cathodes on the other hand can be designed without PFAS-based components, since the electrode can be produced using water, carboxymethyl cellulose, styrene-butadiene rubber and carbon." They also stated that PBA should not be considered a drop-in solution considering that the common battery production lines would have to be adjusted. (#6133, #8801)

Thus, we extrapolate the conclusions regarding alternatives for the use Li-ion/LFP chemistry/Cathode to the use Na-ion/PBA chemistry/Cathode. Overall, no information was available for the phosphate-based chemistry. However, there were indications that the layered metal oxide chemistry for Na-ion batteries are similar to those of L-ion batteries which also is consistent with our expert knowledge.

While the Annex XV consultation comments described that PBA technology has not yet been commercialized, we note that this was based on available information in year 2023. (#8680, #8801). As a result, we conclude that…

- (through extrapolation) it is *very likely* that Na-ion/Layered metal oxide/Cathode is a **PFAS use**
- (through extrapolation) some potential alternatives were identified for Na-ion/Layered metal oxide/Cathode
- it is *only likely for certain applications* that Na-ion/PBA chemistry/Cathode is a **PFAS use**,
- several potential alternatives were identified for the use Li-ion/PBA chemistry/Cathode
- The (lack of) relevant supporting evidence could not confirm or contradict the listed potential use of Na-ion/Phosphate based chemistry/Cathode preventing a conclusion on the availability of potential alternatives.

4.6.2. Anode

One potential use was listed for Na-ion battery anodes: Na-ion/Hard carbon anode in PBA chemistry/Anode based on sufficient information available in one record (Table 4; potential PFAS use no. 24).

Overall, there was no supporting evidence describing this use. As a result, we conclude that…

- The (lack of) relevant supporting evidence could not confirm or contradict the listed potential use of Na-ion/Hard carbon anode in PBA chemistry/Anode preventing a conclusion on the availability of potential alternatives.

4.6.3. Electrolyte

One potential use was listed for Na-ion battery electrolytes: Na-ion/Liquid electrolyte/Electrolyte based on sufficient information available in one record listing the substance NaTFSI (Table 4; potential PFAS use no. 25).

NaTFSI was listed by the Background Document, most likely linked to the use of electrolytes in “sodium-ion rechargeable batteries” stemming from the same consultation comment as in our work.[11]

Overall, as with Li-ion batteries, in which the lithium salt of PF_6 is included as the main salt, we consider it possible to extrapolate the conclusions for Li-ion electrolytes to those of Na-ion batteries. Thus, we conclude that only for certain applications it is likely that the liquid electrolyte of Na-ion batteries is a PFAS use. However, when extrapolating the results for availability of potential alternatives, we note that Na-ion batteries are less often used in high performance applications in which high temperature stability is required. Thus, we consider it possible that several potential alternatives are identified (i.e., formulations in which the TFSI additive is not required). As a result, we conclude that…

- (through extrapolation) it is *only likely for certain applications* that Na-ion/Liquid electrolyte/Electrolyte is a **PFAS use**,
- as a direct result potential alternatives were identified for uses where these requirements are not set. In addition, when considering the application, it is unlikely that high requirements are set for Na-ion batteries, thus indicating that several potential alternatives are identified.

4.6.4. Separator

One potential use was listed for Na-ion battery separator: Na-ion/Liquid electrolyte/Separator based on sufficient information available in one record (Table 4; potential PFAS use no. 26).

Na-ion battery separators were only described by one battery stakeholder and in one of the literature reviews. First, *KURZ* stated that battery separators are very similar across battery technologies, indicating that the interpretation of use and availability of alternatives for Li-ion battery separators in liquid electrolytes could be extended to Na-ion battery separators in liquid

electrolytes.[56] However, Babiker et al. (2023) reported that Li-ion batteries and Na-ion batteries have different liquid electrolytes, which impose different conditions for the separator. According to the study, the commercially available PP and/or PE separators exhibit poor wettability in Na-ion batteries.[102] Still, there was no indication that PFAS-based separators are used in Na-ion batteries with liquid electrolytes.

Overall, we combine the limited supporting evidence with an extrapolation of the available evidence for separators in Li-ion batteries. There is some evidence that other than PP and/or PE separators are used in Na-ion batteries, compared to it being very likely that PP and/or PE separators are used in Li-ion batteries. The extrapolation increases the uncertainty in our conclusion, reducing the rating by one point, and as a result, we conclude that …

- (through partial extrapolation) it is *unlikely* that Na-ion/Liquid electrolyte /Separator is a **PFAS use,**
- overall, several potential alternatives were identified.

4.7. Lithium metal and solid-state batteries

Nine potential uses were connected either to the lithium metal technology (referring to the lithium metal anode) and solid-state technology (referring to the non-liquid electrolyte), either separately (five and three potential uses, respectively) or simultaneously (1 potential use). Overall battery technology categories are not always mutually exclusive, as illustrated with the solid-state battery which could in theory refer to many different battery technologies, using a non-liquid electrolyte with a combination of cathode and anode chemistries. Still, considering that these are often considered their own category based on specific requirements, we list them as individual potential uses. Future work could investigate the extent of the overlaps (e.g., between solid-state and lithium metal batteries).

4.7.1. Lithium metal batteries cathodes

Five potential uses were connected directly to lithium metal batteries: the five Lithium metal/LFP, LCO, LMO, NMC, or NCA chemistries/Cathode based on sufficient information available in one record (Table 4; potential PFAS use no. 27-31). Substances listed within the latter use include e.g., 1,1,2,2- tetrafluoroethyl, 2,2,3,3-tetrafluoropropyl ether, 3,3,3-trifluoropropylene carbonate and perfluorinated ethers.

Lithium metal batteries are briefly described by the Background Document, which most likely stems from the same Annex XV consultation comments as included in this work, where the "binders in active material masses" under the section "use/application" (denoted as "lithium metal rechargeable") is grouped with many other primary and secondary batteries.[11] However, there is no further discussion linked to this use.

Overall, lithium metal batteries refer to the use of lithium metal as the anode instead of graphite, which is used in Li-ion batteries. Thus, we consider it possible to extrapolate the conclusions for Li-ion batteries to these corresponding uses. Lithium metal batteries are used for high-performance batteries, as the metal electrode results in an increase of energy density, and therefore, it is likely that the requirements would be higher for this battery technology. Connecting the likelihood of higher performance requirements in lithium metal batteries as well as increased uncertainty linked to extrapolation, we decrease the certainty of alternatives rating from the extrapolated conclusions from Li-ion batteries by one point. As a result, we conclude that …

- (through extrapolation) it is *likely* that Lithium metal/LFP, LCO, NMC, NCA or LMO chemistry/Cathode is a **PFAS use** and overall, *potential alternatives* were identified *only for certain applications* within these five confirmed uses, except for LFP chemistry for which some potential alternatives were identified.

4.7.2. Lithium metal anode with solid-state electrolyte

One potential use was linked to both a lithium metal and solid-state technology: Lithium metal/Polymer electrolyte/Electrolyte based on sufficient information available in one record (Table 4; potential PFAS use no. 32). This describes a battery that has a lithium metal anode and a solid-state (polymeric) electrolyte.

The OECD reports a polymer electrolyte, but does not link it to a specific battery technology or chemistry.[20] *E-Lyte* stated that TFSI is required as additives in electrolytes of lithium metal batteries.[43] This potential PFAS use highlights the large variety of battery technologies, where the cathode, anode and electrolyte are possible to be modified by battery manufacturers. As a result, it is difficult to overall find conclusions from the supporting evidence, as there is no information available for all three components simultaneously.

As there was no information available from supporting evidence, we had sought to extrapolate the results from similar battery technologies. However, there was also a lack of supporting evidence for Li-ion batteries. As a result, we conclude that…

- (through partial extrapolation) the (lack of) relevant supporting evidence could not confirm or contradict the listed potential use of Lithium metal/Polymer electrolyte/Electrolyte preventing also a conclusion on the availability of potential alternatives.

4.7.3. Solid-state battery

Three potential uses were linked directly to solid state batteries: Solid state/Solid, Gel or Polymer/Electrolyte based on sufficient information available in three, three and two records, respectively (Table 4; potential PFAS uses no. 33-35).

The use of solid polymer electrolytes is described by previous use-mapping studies by Ameduri (2018; 2020)[13,23] On the other hand, the "Check Your Tech" report published by Chemsec describes a novel battery technology using fluoropolymers as electrolytes, which indicate that they identified a polymer electrolyte for solid-state batteries.[18] A "gel polymer" electrolyte is reported by Ameduri (2018), Glüge et al. (2020) and the US DOE report (2024).[4,13,21]

As there was no information available from supporting evidence, we sought to extrapolate the results from similar battery technologies. However, there was also a lack of supporting evidence for Li-ion batteries with polymer electrolytes. As a result, we conclude that…

- (through partial extrapolation) the (lack of) relevant supporting evidence could not confirm or contradict the three listed potential PFAS uses of Solid state/Solid electrolyte/Electrolyte, Solid state/Gel electrolyte/Electrolyte, and Solid state/ Polymer electrolyte/Electrolyte preventing also a conclusion on the availability of potential alternatives.

4.8. Lead acid battery

One potential use was listed for Lead acid batteries: Lead acid/BMS, catalytic device/Vent based on sufficient information available in one record (Table 4; potential PFAS use no. 36).

The Annex XV report state that lead acid batteries are suitable alternatives to Li-ion batteries.[10] However, many stakeholders to the Annex XV public consultation express disagreement, considering that these batteries have different performances and thus are used in different applications (section 3.5.2). However, the stakeholders still recognize that lead acid batteries do not use fluoropolymers. Still, in our assessment, it is not clear if these comments also include the vent of the lead acid battery or only describe the cell-specific components.

Overall, we consider there is some evidence that lead acid batteries do not use PFASs, but this only stems from one type of source (Annex XV consultation comments) and not to any large

extent. While we also recognize that negative results (i.e. providing evidence of non-PFAS use) are less likely to be submitted than positive result (i.e. providing evidence of PFAS use), we conclude that…

- The (lack of) relevant supporting evidence could not confirm or contradict the listed potential use of Lead acid/BMS, catalytic device/Vent preventing also a conclusion on the availability of potential alternatives.

REFERENCES

Automatic citation updates are disabled. To see the bibliography, click Refresh in the Zotero tab.
